# Cryo-EM structure of human telomerase dimer reveals H/ACA RNP-mediated dimerization

**DOI:** 10.1126/science.adr5817

**Published:** 2025-07-10

**Authors:** Sebastian Balch, Zala Sekne, Elsa Franco-Echevarría, Patryk Ludzia, Rachael C Kretsch, Wenqing Sun, Haopeng Yu, George E Ghanim, Sigurdur Thorkelsson, Yiliang Ding, Rhiju Das, Thi Hoang Duong Nguyen

**Affiliations:** 1 MRC Laboratory of Molecular Biology; Cambridge, CB2 0QH, UK; 2 Biophysics Program, Stanford University; Stanford, CA 94305, USA; 3 Key Laboratory of Molecular Epigenetics of the Ministry of Education, Northeast Normal University, Changchun 130024, China; 4 Department of Cell and Developmental Biology, John Innes Centre, Norwich Research Park, Norwich NR4 7UH, UK; 5 Department of Biochemistry, Stanford University; Stanford, CA 94305, USA; 6 HHMI, Stanford University; Stanford, CA 94305, USA

## Abstract

Telomerase ribonucleoprotein (RNP) synthesizes telomeric repeats at chromosome ends using a telomerase reverse transcriptase (TERT) and a telomerase RNA (hTR for humans). Previous structural work showed that human telomerase is typically monomeric, containing a single copy of TERT and hTR. Evidence for dimeric complexes exists, although the composition, high-resolution structure and function remain elusive. Here, we report the cryo-electron microscopy structure of a human telomerase dimer bound to telomeric DNA. The structure reveals a 26-subunit assembly and a dimerization interface, to which premature aging disease mutations map. Disrupting dimer formation affects RNP assembly, bulk telomerase activity, and telomere maintenance in cells. Our findings address a longstanding enigma surrounding the telomerase dimer and suggest a role for the dimer in telomerase assembly.

Telomeres, situated at the ends of linear eukaryotic chromosomes, undergo shortening during each cell cycle due to incomplete genome replication (1). Telomerase is a ribonucleoprotein (RNP), which preserves telomere length by adding telomeric repeats (GGTTAG in humans) to the chromosome 3’ termini (2, 3). Up to 90% of cancer cells confer cellular immortalization through telomerase activation (4, 5). Conversely, mutations that compromise telomerase function contribute to a diverse range of premature aging diseases, such as dyskeratosis congenita (6, 7).

The enzymatic activity of telomerase relies on a telomerase reverse transcriptase (TERT) and an internal RNA template contained in the telomerase RNA (TER or hTR in humans) (2). The human telomerase holoenzyme contains 11 accessory subunits, including histone H2A-H2B dimer, two sets of the H/ACA heterotetramers (dyskerin, NOP10, NHP2 and GAR1) and telomerase Cajal body localization factor, TCAB1 (8, 9). Recent cryo-electron microscopy (cryo-EM) structures revealed the segregation of human telomerase subunits into two lobes flexibly connected by hTR (8–10). In one lobe, TERT and histone H2A-H2B bind two activity-essential domains of hTR: the pseudoknot/template domain (PK/t) and conserved regions 4 and 5 (CR4/5) (Fig. 1, A and B). This lobe, known as the catalytic core, is the site where telomeric DNA synthesis occurs. In the other lobe, the H/ACA proteins and TCAB1 associate with the H/ACA domain of hTR to form the H/ACA RNP (Fig. 1, A and B), which plays a crucial role for telomerase biogenesis (11–13).

There have been reports suggesting the existence of a dimeric form of human telomerase. Size fractionation experiments indicated that the molecular weight of human telomerase was in the range of 550–670 kDa (14–17). Human telomerase was, thus, proposed to be dimer of TERT, hTR and dyskerin (16, 17). However, extensive evidence suggests that human telomerase functions as a monomer of TERT and hTR (8–10, 18–22). A monomeric telomerase holoenzyme, consistent with the composition revealed by recent structural studies (578 kDa), would exhibit a comparable size fractionation pattern (8, 9, 21). Therefore, relying on size alone does not definitively distinguish between the proposed dimeric composition and the monomeric composition observed by cryo-EM.

Additional support for dimeric human telomerase comes from purification of telomerase with two differently tagged TERT or two different constructs of hTR template sequence, and single-molecule experiments (15, 17, 19). These studies demonstrated the presence of telomerase RNPs with two copies of either TERT or hTR in addition to monomeric telomerase RNPs. However, it remains unclear from these studies whether both TERT and hTR simultaneously exist in duplicate and whether other telomerase accessory subunits are also present. Without a comprehensive understanding of the structure, composition and molecular interactions that drive dimerization, the functional role of such dimeric telomerase remains unknown.

## A population of human telomerase forms dimers

**Fig. 1:**
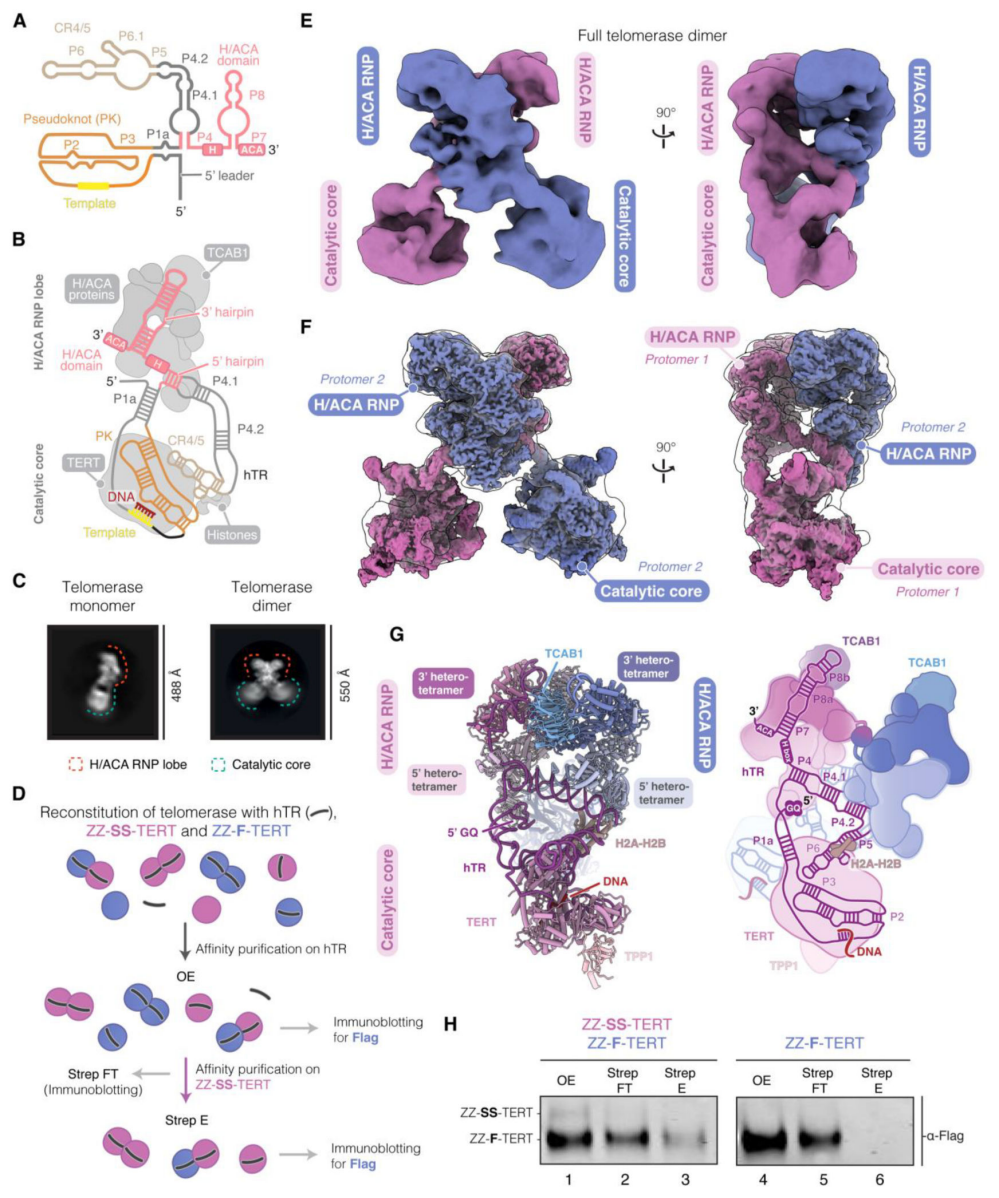
Structure of the human telomerase dimer. **(A)** Schematic showing secondary structure of hTR. **(B)** Schematic of human telomerase monomer. The domains of hTR are colored as shown in (A). **(C)** Representative cryo-EM 2D class averages of the telomerase monomer and the telomerase dimer. **(D)** Schematic of the purification strategy to probe for the human telomerase dimer using ZZ-SS-TERT and ZZ-F-TERT. ZZ, protein A; SS, twin-Strep tag; F, Flag tag; OE, oligonucleotide elution; Strep FT, Strep-Tactin flow-through; Strep E, Strep-Tactin elution. **(E)** 6.2 Å consensus cryo-EM map of the full human telomerase dimer. The two protomers are colored in magenta and blue. **(F)** The consensus cryo-EM map of the full telomerase dimer with fitted two copies of the 3.0 Å cryo-EM reconstruction of the human telomerase H/ACA RNP protomer and the 3.3 Å cryo-EM reconstruction of the human telomerase catalytic core. These composite maps were obtained from focused classification and refinement on the H/ACA RNP and the catalytic cores separately. **(G)** Atomic model of the full telomerase dimer (left) as shown in (F) and the schematic of the structure (right) as shown in (F). The model was obtained through a combination of fitting published models, manual building and DRRAFTER modeling (23, 26, 27). **(H)** Immunoblots of the telomerase sample with ZZ-SS-TERT and ZZ-F-TERT (left), and control telomerase with ZZ-F-TERT (right) obtained by purification strategy depicted in (D). An anti-Flag antibody was used.

We initially collected cryo-EM data of human telomerase with the telomerase recruitment complex, TPP1-POT1-TIN2 (TPT), and telomeric DNA to understand telomerase interactions with the DNA substrate (fig. S1, A and B). Close inspection of two-dimensional (2D) class averages revealed a small subset of particles featuring two attached telomerase molecules (Fig. 1C and fig. S1C). We devised a data processing strategy to enrich for such particles across four independent cryo-EM datasets and obtained a 6.2 Å consensus map (see Materials and methods; fig. S2; fig S3, A to D; Table S1). By unambiguously fitting the map with published structures of human telomerase (9, 23), we verified the identity of these particles as telomerase homodimer (Fig. 1, E to G).

To test whether the telomerase dimer is formed during telomerase reconstitution in human cells, we used a split nano-luciferase assay. In this assay, cells were co-transfected with hTR and TERT containing split nano-luciferase constructs, named LgBiT and SmBiT (fig. S4A) (24). Bioluminescence is obtained when the LgBiT and SmBiT fragments are in close proximity. Indeed, we detected bioluminescence only in cells transfected with two TERT constructs, one carrying a LgBiT and the other carrying SmBiT (fig. S4B). This suggests that telomerase complexes with two TERT molecules in close proximity form in cells.

To detect the presence of the human telomerase dimer biochemically, we prepared telomerase with a combination of two differentially tagged TERTs: ZZ-3xFlag-TERT (ZZ-F-TERT) and ZZ-twin-Strep-SUMO*-TERT (ZZ-SS-TERT). We then employed a two-step purification strategy involving oligonucleotide-affinity purification (O-purification) on hTR followed by Strep-Tactin pulldown, a strategy similar to that used for cryo-EM sample preparation (Materials and methods, Fig. 1D). Immunoblots of the resulting Strep-Tactin elution with an anti-Flag antibody revealed the co-purification of ZZ-F-TERT (Fig. 1H, lane 3). No non-specific binding of ZZ-F-TERT to the Strep-Tactin resin was detected (Fig. 1H, lane 6). This demonstrates the existence of telomerase RNP containing both tagged versions of TERT simultaneously. Furthermore, the intensity of the Flag-tagged TERT band in the Strep-Tactin elution was weak relative to that in the oligonucleotide elution (OE) fraction (Fig. 1H, lanes 1 and 3). Hence, the dual-tagged complexes constitute only a small fraction of our reconstituted complexes, consistent with the low particle distribution of the dimer in our cryo-EM data (fig. S2).

To further confirm that the dual-tagged telomerase is the telomerase dimer, we isolated these particles using a double pulldown on both the Flag and Strep tags following the O-purification and analyzed them using negative stain EM (fig. S5, A and C). The 2D class averages of these particles resemble those of the dimeric particles observed in the cryo-EM data (fig. S5, D to F), confirming their identity as telomerase dimers.

## The telomerase dimer structure reveals its composition and an unexpected dimerization interface

**Fig. 2:**
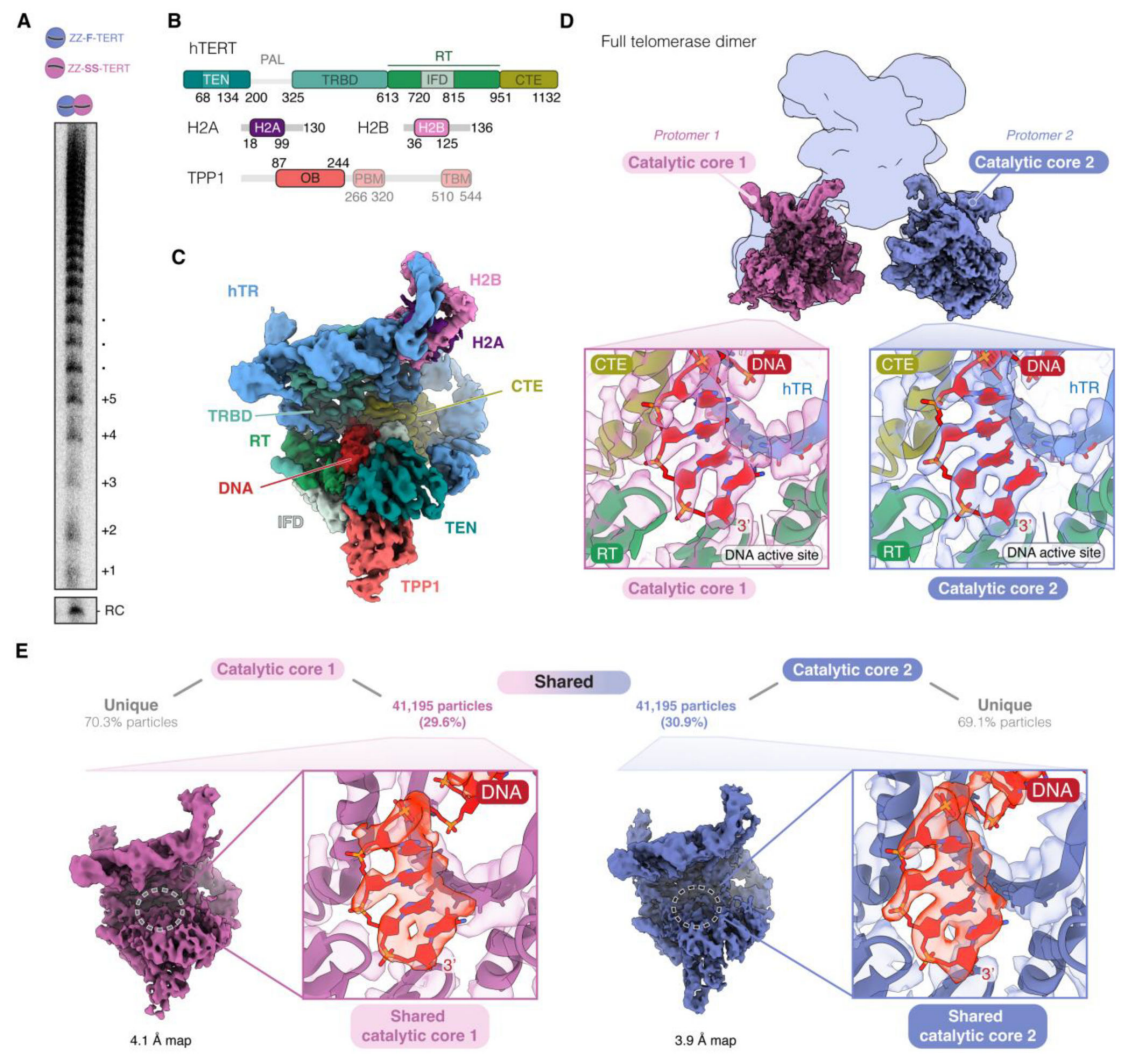
The human telomerase dimer is catalytically active. **(A)** Telomerase activity assay of the purified dual-tagged human telomerase dimer following the procedure depicted in Fig. S5A. RC, recovery control. **(B)** Domain organization of human TERT (hTERT), histones H2A and H2B, and TPP1. TEN, telomerase essential N-terminal; PAL, proline/arginine/glycine-rich linker; TRBD, telomerase RNA binding domain; RT reverse transcriptase; IFD, insertion in the fingers domain; CTE, C-terminal extension; OB, oligonucleotide/oligosaccharide binding; PBM, POT1 binding motif; TBM, TIN2 binding motif. **(C)** 3.3 Å cryo-EM map of the human telomerase catalytic core using C2-expanded particles. Domains are labelled and colored according to the color scheme in (B). **(D)** The consensus cryo-EM map of the full human telomerase dimer with fitted 3.8 Å and 3.6 Å cryo-EM reconstructions of the human telomerase catalytic core 1 and 2, respectively. The maps were generated without applying symmetry functions. Insets show the close-up views of the DNA-bound telomerase active site of the catalytic core 1 (left) and catalytic core 2 (right). **(E)** Data processing strategy used to obtain the human telomerase catalytic cores from the shared particles, which originated from the subsets that gave the reconstructions of the two catalytic cores shown in (D). The shared particles are those found in both particle stacks used to generate catalytic core 1 and catalytic core 2 maps. The maps of telomerase catalytic cores 1 and 2, obtained from only the shared particles, are shown with the DNA in the active sites.

The human telomerase dimer contains twice the number of subunits present in the telomerase holoenzyme (Fig. 1, B, E to G) (9). In the dimer structure, the two protomers intertwine in an X-shaped architecture, showcasing a near two-fold symmetry axis passing through the dimer interface (Fig. 1, E and F). Various regions of TERT within the catalytic core have been suggested to mediate dimer or multimer formation (19, 25). However, our cryo-EM structure reveals that dimerization is primarily facilitated by interactions between the two H/ACA RNPs, with the two catalytic cores positioned away from the dimerization interface (Fig. 1E, 1F and 2D).

To resolve the dimerization interface, we performed signal subtraction and focused classification on the two H/ACA RNPs within the structure. We obtained a 3.0 Å resolution map for a single H/ACA RNP protomer and a 3.9 Å resolution map for the two H/ACA RNPs containing most of the dimerization interface (Fig. 1F; fig. S1D; fig. S2; fig. S3, E to L; fig. S6; Table S1). Henceforth, we refer to the latter reconstruction as the H/ACA RNP dimer. The improved resolution enabled model building, particularly of regions involved in dimerization (fig. S6; Table S1). We then docked atomic models of the catalytic cores bound to the recruitment factor TPP1 and the H/ACA RNP dimer into the consensus map, combined with RNA modeling by DRRAFTER, to obtain a complete atomic model for the dimeric telomerase complex (Fig. 1G; fig. S7; Data S1) (23, 26, 27).

## The telomerase holoenzyme dimer is catalytically active

We asked whether dimerization impacts the ability of TERT to bind and extend telomeric DNA. Using telomerase activity assay, we confirmed that the purified dual-tagged dimer can extend telomeric DNA (Fig. 2A; fig. S5, A and B). This finding aligns with a previous study (15) but contrasts with another report that found no activity following tandem affinity purification of differentially tagged TERT (20). However, it is worth noting that the input lysate used by Errington et al. (20) was approximately 480-fold less than what we used (Materials and methods, fig. S5A). Given the low abundance of the dimer, the activity in this previous study (20) was likely below the detection threshold of their telomerase activity assay, which explains the observed discrepancy with our results.

We next examined the DNA binding state of the catalytic core in the dimer structure. By applying symmetry, we obtained a reconstruction at 3.3 Å resolution for a catalytic core protomer in the context of the telomerase dimer (Fig. 2, B and C; fig. S1E; fig. S2; fig. S8, A to D). Consistent with our activity assay, a telomeric DNA substrate is bound in the active site of TERT (Fig. 2C; fig. S9, A and B; Table S2). Hence, our structure represents a functionally active dimer.

## The proline/arginine/glycine-rich region of TERT is not involved in the dimerization of the telomerase holoenzyme

TERT consists of four domains: telomerase essential N-terminal (TEN) domain, telomerase RNA binding domain (TRBD), reverse transcriptase (RT) and C-terminal extension (CTE) (Fig. 2B). The TEN domain and TRBD are connected by a disordered proline/arginine/glycine-rich linker (PAL) (Fig. 2B). Previous studies showed that disrupting the PAL reduced dimerization/oligomerization of overexpressed TERT. However, once assembled into the telomerase RNP, the PAL mutant showed no defects in telomerase activity (19). In our dimer structure, we did not observe direct interactions between the two catalytic cores. The PAL regions of the two TERTs were unresolved due to flexibility. To investigate whether the two PAL regions in our dimer structure may be interacting with each other, we tested the role of the PAL in telomerase dimer formation.

We reconstituted telomerase with ZZ-F-TERT and ZZ-SS-TERT, both lacking the PAL (ΔPAL). Following O-purification and Strep-Tactin pulldown, we assessed the co-purification of ZZ-F-TERT in the Strep-Tactin elution (Fig. 1D and fig. S10A). We found that the level of ZZ-F-TERT in the ΔPAL mutant was slightly greater than that in the wild-type telomerase (fig. S10, B and C), suggesting that telomerase with the ΔPAL TERT can still dimerize. We also observed a notable increase in dyskerin levels in the ΔPAL mutant telomerase compared to the WT (fig. S10, B and D). Dimerization of TERT protein via the PAL has been proposed to occur in hTR-free TERT or in inactive RNP (19). Another study described such inactive RNP with a morphology distinct from the dimer observed in this study (fig. S5G) (8). Removing the PAL may reduce this unproductive dimerization of TERT, thereby improving RNP assembly and explaining the increased levels of both ZZ-F-TERT and dyskerin observed in the ΔPAL mutant.

We also isolated the ΔPAL dimer using a three-step purification process (fig. S10E). The purified ΔPAL dimer remained catalytically active, consistent with previous findings that PAL is dispensable for telomerase activity (fig. S10F) (19). Negative stain EM 2D class averages of the ΔPAL dimer were similar to those of the WT dimer (fig. S10, G to I), further confirming that PAL deletion does not affect telomerase dimerization. Our results demonstrate that the PAL-mediated TERT dimerization is distinct from the dimerization of telomerase holoenzyme reported in this study and may occur at an earlier stage of telomerase assembly.

## The two catalytic cores operate independently

Previous biochemical and kinetic studies proposed that the two active sites in a telomerase dimer act cooperatively or in tandem (15, 28). To determine whether both catalytic cores can simultaneously bind to a DNA substrate, we performed signal subtraction to focus on each of the two catalytic cores individually (fig. S2). The best subsets of particles for the two catalytic cores yielded reconstructions at 3.6 Å and 3.8 Å resolution, showing clear densities for bound DNA substrates and TPP1 (Fig. 2D; fig. S8, E to L; fig. S9, C and D; Table S2).

A substantial proportion of particles in these two independently processed subsets originated from the same non-subtracted dimeric particles. Reconstructions of the two catalytic cores from this common subset of particles showed clear density for the DNA in the active site of TERT (Fig. 2E). This demonstrates the presence of dimeric telomerase with both catalytic cores bound to separate telomeric DNA substrates. Given the independent engagement of DNA and the lack of interactions between the two catalytic cores, our structure does not explain the previously observed cooperativity between the two active sites.

To assess the potential cooperativity between the two catalytic cores in our telomerase dimer, we purified a dimer containing one WT TERT and one catalytically inactive TERT mutant (D712A/D868A/D869A) and compared its activity to that of a dimer with two WT TERTs (fig. S11, A and B). Telomerase activity was proportional to the amount of active TERT present, indicating no cooperativity between the catalytic cores (fig. S11, C and D). We obtained similar results in an independent experiment, where we used glycerol gradient centrifugation to separate the monomeric telomerase complex from the dimeric complex (fig. S12A). Telomerase activity of the gradient fractions correlated with TERT levels; and the dimer peak fraction did not show increased processivity compared to the monomeric fraction (fig. S12, B to D). Although additional factors may coordinate the two catalytic cores in cells, our structural and biochemical data suggest that the two catalytic cores function independently *in vitro*.

## The P4.2 stem of hTR is critical for dimerization

The H/ACA domain of hTR exhibits a dual-hairpin structure, characteristic of RNAs within the H/ACA RNP family (Fig. 1, A and B) (21, 29). Each RNA hairpin associates with four H/ACA proteins including dyskerin, NHP2, NOP10 and GAR1 (30). We refer to the H/ACA proteins as either the 5’ or 3’ heterotetramers, depending on whether they are bound to the 5’ or 3’ hairpin of hTR, respectively.

**Fig. 3:**
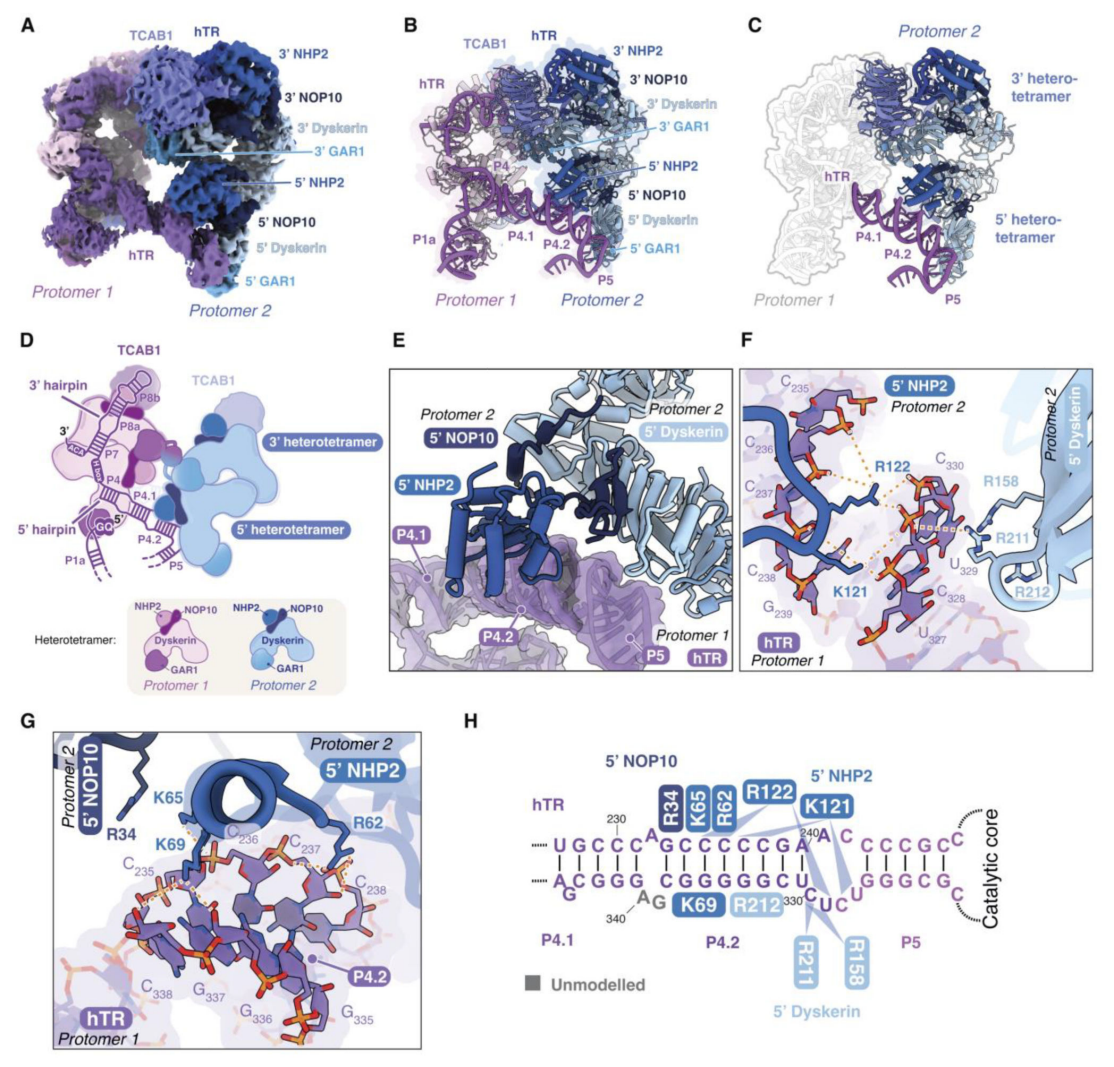
Dimerization relies on interactions between the two H/ACA RNPs. **(A)** 3.9 Å cryo-EM map of the H/ACA RNP dimer with individual protomers colored. Only subunits of protomer 2 (blue) and hTR of protomer 1 are labelled for simplicity. **(B, C)** A cartoon representation of the telomerase H/ACA RNP dimer model. In (C), only protomer 2 and the P4/5 linker of protomer 1 are highlighted in colors. **(D)** Schematic of the H/ACA RNP dimer. **(E)** A close-up view of the inter-protomer interactions between the hTR of protomer 1 and the 5’ H/ACA heterotetramer of protomer 2. The P4/5 linker interacts with the 5’ dyskerin, 5’ NHP2 and 5’ NOP10. **(F)** A close-up view of the interactions between the hTR P4.2/P5 junction of protomer 1 and the 5’ dyskerin and 5’ NHP2 of protomer 2. **(G)** A close-up view of the interactions between the hTR P4.2 stem of protomer 1 and the 5’ NHP2 and 5’ NOP10 of protomer 2. **(H)** Schematic of the interactions between the P4/5 linker of hTR from protomer 1 and the 5’ H/ACA proteins of protomer 2.

In telomerase, the H/ACA RNP is connected to the catalytic core through two linkers in hTR: the P1 linker, formed by the P1a stem, and the P4/5 linker, consisting of the P4.1, P4.2, and P5 stems (Figs. 1, A and B, 3D; fig. S7A). The P1 and P4/5 linkers of hTR were unresolved in previous high-resolution structures of telomerase due to their flexibility (9, 10, 22). In our dimer structure, the two H/ACA RNPs pack against each other through an intricate network of protein-RNA and protein-protein interactions (Fig. 3, A to D), which rigidifies the RNA linkers in hTR. This limits the relative conformations between the lobes of each protomer (Movie S1). Our dimer structure allowed for a detailed visualization of these RNA linkers and unveiled their role in telomerase dimerization, as described below (fig. S6, E, F and K).

The P4/5 linker of hTR from one protomer binds to a cleft formed by the 5’ dyskerin, 5’ NOP10, and 5’ NHP2 of the other protomer (Fig. 3, E to H). The P4.2 stem is anchored by residues Lys121 and Arg122 of the 5’ NHP2 and further stabilized by other residues in the 5’ NHP2 (Arg62, Lys65, and Lys69) and NOP10 (Arg34) (Fig. 3, F to H). The junction between the P4.2 and P5 stems (nucleotides 327–330) also engages in backbone interactions with the 5’ dyskerin, specifically with residues Arg158, Arg211, and Arg212 (Fig. 3, F and H).

**Fig. 4:**
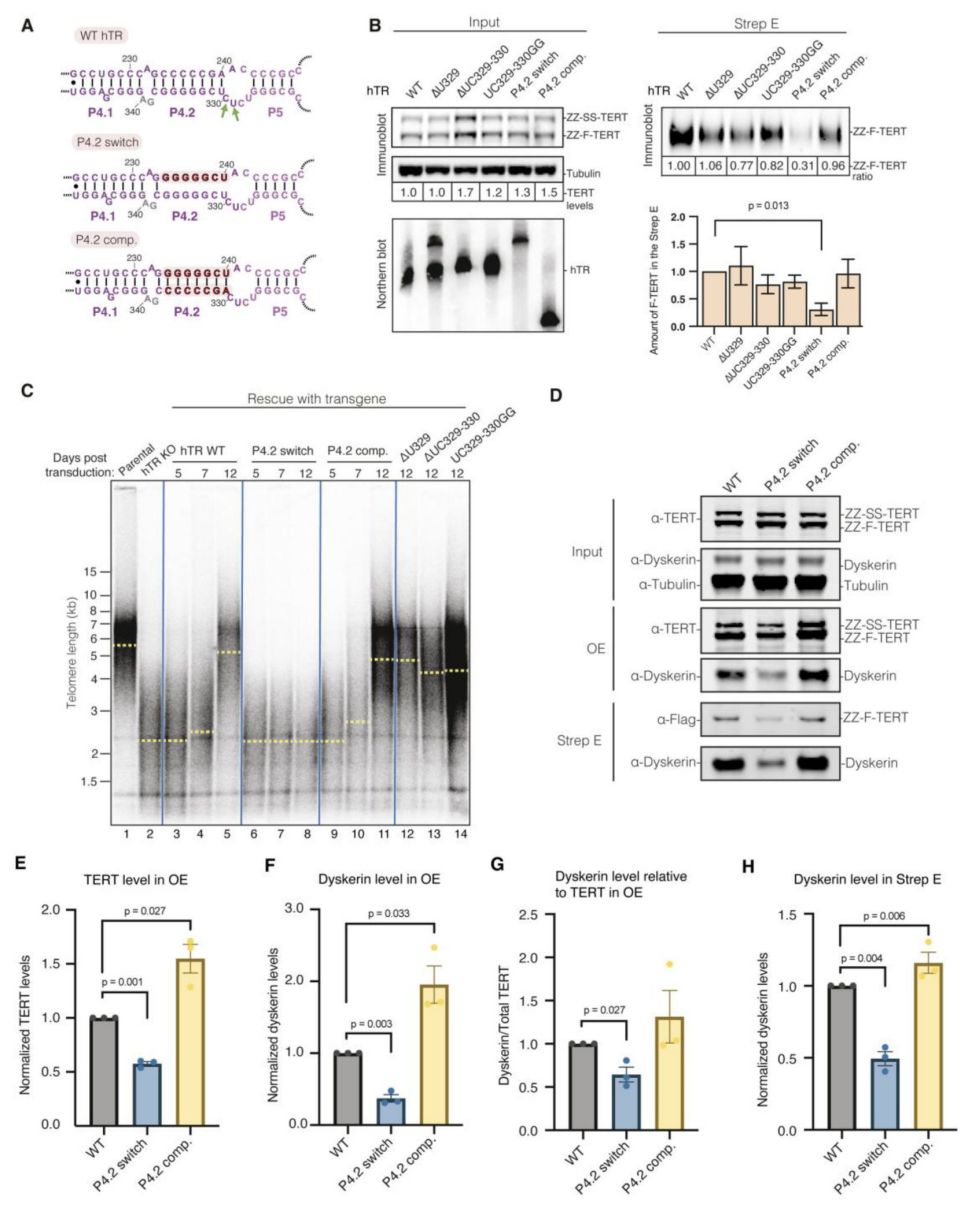
Disrupting the P4.2 stem of hTR affects the binding of dyskerin and telomere maintenance in cells. **(A)** Schematic of WT hTR P4.2 stem, P4.2 switch and P4.2 compensatory (P4.2 comp.) hTR mutants. Nucleotides 329 and 330 of hTR (highlighted in with green arrows) are also mutated in experiments shown in (B) and (C). Nucleotides mutated to create the P4.2 switch and P4.2 comp. mutants are shown in red. **(B)** Immunoblots and northern blots of the human telomerase reconstituted with the ZZ-SS-TERT, ZZ-F-TERT, and the WT or mutant hTR. In the left panel, TERT levels in telomerase lysates (input) are normalized to tubulin levels. Telomerase was purified as described in Fig. 1D. The right panel depicts the immunoblot and corresponding quantification of the levels of the ZZ-F-TERT detected in the Strep elution (Strep E). ZZ-F-TERT levels in the Strep E of mutants are normalized to that in the WT sample. The bar graph represents the amount of ZZ-F-TERT in the Strep E assembled with the mutant hTR compared to the WT hTR. Experiments were performed in triplicates (n = 3). Error bars represent the standard error of the mean (SEM), and significant p value is reported for the P4.2 switch hTR mutant. See fig. S13 for the replicate data. **(C)** Telomere restriction fragment (TRF) assay of HCT116 hTR knockout (KO) cell lines transduced with either WT or mutant hTR from (A). Lanes 1 and 2 show telomere lengths of the parental HCT116 and the KO cell lines, respectively. Lanes 3 to 14 show telomere lengths of KO cells that were rescued with various hTR transgene constructs. Yellow dash lines indicate the mean telomere length for each lane. **(D)** Immunoblots of the input lysates (input), OE, and strep E to dissect the effects of disrupting P4.2 stem of hTR. Each of hTR constructs, either WT or P4.2 switch or P4.2 comp. mutant, was reconstituted with a mixture of ZZ-SS-TERT and ZZ-F-TERT (fig. S14A). Telomerase was purified via the O-purification and Strep-Tactin pulldown, as shown in fig. S14A. Experiments were performed in triplicates (n = 3). See fig. S14B for replicate data. **(E)** Bar graph showing the levels of TERT in the OE of WT hTR, P4.2 switch and P4.2 comp. mutants. **(F)** Bar graph showing the levels of dyskerin in the OE of WT hTR, P4.2 switch and P4.2 comp. mutants. **(G)** Bar graph showing the levels of dyskerin relative to TERT in the OE of WT hTR, P4.2 switch and P4.2 comp. mutants. Together with the graphs shown in (E) and (F), this graph shows that in the OE, dyskerin levels in the P4.2 switch hTR mutant were more severely reduced than TERT compared to the WT hTR. **(H)** Bar graph showing the levels of dyskerin in the Strep E of WT hTR, P4.2 switch and P4.2 comp. mutants. In the quantification shown in (E) to (H), TERT or dyskerin levels of the mutants were normalized to those of the WT hTR. Error bars in (E) to (H) represent the standard error of the mean (SEM), and significant p values are also reported.

To validate these inter-protomer protein-RNA interactions, we generated hTR mutants with disrupted base-pairing in the P4.2 stem or perturbed P4.2/P5 junction (Fig. 4A; fig. S13). We reconstituted these mutants with a 1:1 mixture of ZZ-F-TERT and ZZ-SS-TERT, followed by O-purification and Strep-Tactin pulldown as described above (Fig. 1D). We probed for Flag-tagged TERT in the Strep-Tactin elution as a readout for dimer formation. Consistent with our structure, disrupting the base-pairing of the P4.2 stem (P4.2 switch) severely reduced dimer formation (Fig. 4B and fig. S13). This defect was restored using a compensatory mutant (P4.2 comp.), where the two strands of P4.2 stem were swapped while maintaining base-pairing (Fig. 4, A and B). Mutations at the P4.2/P5 junction (Δ329, ΔC329–330 and UC329–330GG) did not significantly reduce dimer formation (Fig. 4B). Since the P4.2 stem forms most extensive interactions with the 5’ H/ACA proteins (Fig. 3E), these results agree with our structure and demonstrate that the P4.2 stem of hTR is critical for dimer formation.

## Disrupting dimerization impairs telomere maintenance in cells and assembly of active telomerase RNP

To investigate the impact of disrupting dimerization in cells, we utilized a hTR knockout HCT116 cell line, which exhibits critically short telomeres (31), and performed rescue experiments using either WT or mutant hTR (P4.2 switch, P4.2 comp., Δ329, ΔC329–330 and UC329–330GG). WT and mutant hTR were stably expressed in this cell line by transgene integration at the AAVS1 safe-harbour locus (31). Without transgene expression, telomere restriction fragment (TRF) analysis showed that the hTR knockout cells exhibit telomere shortening and eventually undergo cell death (Fig. 4C, lanes 1 and 2). Expression of the WT hTR efficiently rescued telomere shortening, restoring the telomere length to parental levels (Fig. 4C, lanes 1, and 3 to 5), whereas the P4.2 switch mutant did not (Fig. 4C, lanes 6 to 8). In contrast, the P4.2 compensatory mutant restored telomere length to a similar level as the WT hTR (Fig. 4C, lanes 9 to 11). Mutations in the P4.2/P5 junction, which did not substantially affect dimer formation, also rescued telomere shortening but to a lesser extent than WT hTR (Fig. 4C, lanes 12 to 14). These results validate our structural and biochemical work and suggest that human telomerase dimerization is important for telomere maintenance in cells.

We next sought to determine the functional role of telomerase dimerization. We first examined the effect of disrupting dimerization on telomerase activity. Although the P4.2 stem of hTR is distant from the telomerase active site, telomerase activity was severely impaired in the P4.2 switch mutant (fig. S13). Restoring base-pairing within the P4.2 stem rescued the activity defects, as shown in the P4.2 comp. mutant (fig. S13). Given that the telomerase dimer is a minor population in our sample, the bulk activity observed likely corresponds to that of the more abundant monomeric complex. Our data suggest that disrupting dimerization affects the activity of the monomeric complex and imply that the dimer may be a biogenesis precursor of the monomer. Therefore, we hypothesized that dimerization may have a role in telomerase assembly.

To test this hypothesis, we examined the impact of disrupting P4.2 stem on hTR interaction with TERT and dyskerin. We reconstituted either WT hTR or the P4.2 mutant (either switch or comp.) with ZZ-F-TERT and ZZ-SS-TERT as above (Fig. 4, B and D, fig. S14A). Following the O-purification via hTR, we compared the levels of TERT and dyskerin in the OE fractions of the WT hTR and the two P4.2 hTR mutants (Fig. 4D; fig. S14, A and B; OE fractions). The P4.2 switch hTR mutant efficiently pulled down TERT, but with approximately 40% less efficiency compared to WT hTR (Fig. 4, D and E). However, the dyskerin level associated with the P4.2 switch mutant was more significantly reduced than TERT (Fig. 4, D to G). A similar trend was also observed in the second Strep-Tactin purification (Fig. 4, D and H; Strep E fraction). In contrast, the P4.2 comp. hTR mutant rescued the defects in both TERT and dyskerin binding (Fig. 4, D to H). These results are consistent with the cross-protomer interaction between the P4.2 stem of hTR and the 5’ dyskerin in our dimer structure (Fig. 3, A to E).

Given the close proximity of the P4.2 stem to the activity-essential CR4/5 domain of hTR, we examined the folding of the P4.2 stem and the CR4/5 region in the WT hTR and the P4.2 switch and comp. hTR mutants. We reconstituted ZZ-SS-TERT with either P4.2 switch or comp. hTR mutants, followed by O-purification and Strep-Tactin pulldown, which would yield mostly monomeric telomerase RNP (fig. S15A). To assess RNA structural integrity, we conducted RNA dimethylsulfate (DMS) chemical probing experiments on purified P4.2 switch and comp. mutants, comparing their reactivities to WT hTR (fig. S15E). In the WT hTR, the P4.2 stem region exhibited low DMS reactivities, indicating stable base pairing in this region (fig. S15E). In contrast, the P4.2 switch hTR mutant displayed high DMS reactivities across the P4.2 stem region, suggesting stem disruption (fig. S15E). In the P4.2 comp. mutant, DMS reactivities were low across the stem region (fig. S15E), confirming the restoration of base-pairing. Moreover, the P4.2 switch mutant showed relatively similar DMS reactivities across the CR4/5 region compared to the WT hTR, suggesting that disrupting the P4.2 stem did not cause global misfolding of the CR4/5 region (fig. S15E). Therefore, the RNA DMS chemical probing results generally agree with our structural design.

Negative stain EM of the purified P4.2 switch and P4.2 comp. mutants showed that both still formed monomeric telomerase RNPs similar to the WT (figs. S1F, S16, A to F). However, compared to the P4.2 comp. mutant, the P4.2 switch mutant produced fewer assembled telomerase particles relative to the total particle count (fig. S16, A to D). Additionally, the purified P4.2 switch mutant exhibited a marked decrease in telomerase activity per TERT and reduced dyskerin level relative to TERT (fig. S15, B to D). Collectively, these findings indicate that while the P4.2 switch mutant can still bind TERT, many of the resulting TERT/hTR complexes lack dyskerin and are inactive, pointing to an assembly defect.

## Cross-protomer interactions are important for the assembly of H/ACA proteins with hTR

**Fig. 5:**
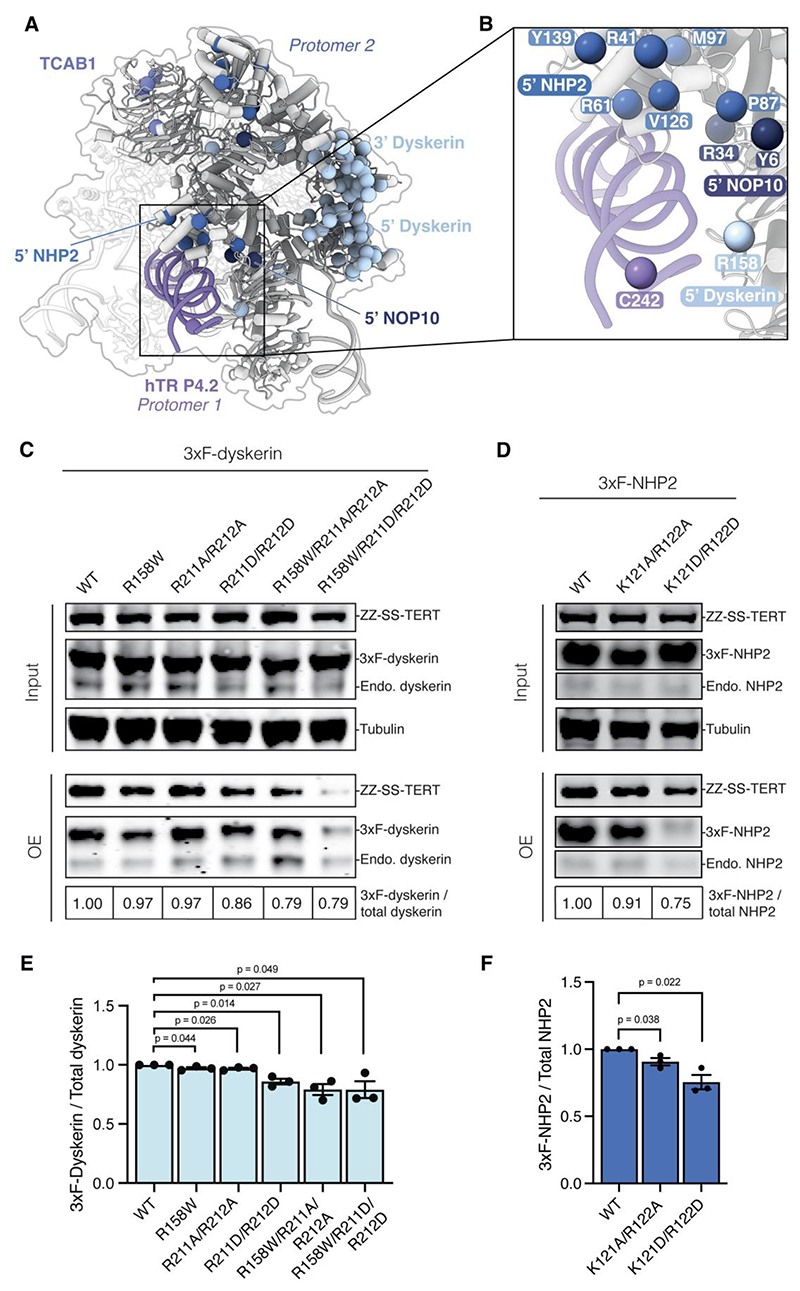
Dimerization plays a role in telomerase assembly. **(A)** Disease mutation mapping of the H/ACA proteins and hTR onto the model of the H/ACA RNP dimer (6, 53). Disease mutations are represented as spheres. **(B)** Inset showing a close-up view of the interface between the hTR P4.2 stem of protomer 1 and the H/ACA proteins of protomer 2 with the disease mutations at this interface. **(C)** Immunoblots of cell lysates (input) and OE from cells transfected with ZZ-SS-TERT, hTR, and WT or mutant 3xF-dyskerin. The total dyskerin levels in the OE were calculated as 3xF-dyskerin plus endogenous dyskerin. Mutant 3xF-dyskerin levels over total dyskerin were normalized to the WT sample. **(D)** Immunoblots of cell lysates and OE from cells transfected with ZZ-SS-TERT, hTR, and either WT or mutant 3xF-NHP2. Analysis and quantification are the same as for (C). **(E)** Bar graph showing the levels of 3xF-dyskerin over the total amount of dyskerin in the OE samples from (C). **(F)** Bar graph showing the levels of 3xF-NHP2 over the total amount of NHP2 in the OE samples from (D). Experiments in (C) and (D) were performed in triplicates (n = 3). Also see fig. S17 for the replicate data. Error bars in (E) and (F) represent standard error of the mean (SEM), and significant p values are reported.

Many premature aging disease mutations in the H/ACA proteins have been shown to affect telomerase assembly (8, 9, 27). We previously mapped most of the disease mutations to the interface between the two dyskerin molecules in the H/ACA RNP of human telomerase (Fig. 5A). The 5’ H/ACA RNA hairpin of hTR is shorter than in canonical H/ACA RNAs, leading to a weaker association with the 5’ H/ACA heterotetramer compared to a canonical H/ACA hairpin (Fig. 1, A and B) (8, 9, 21, 27, 32). The mutations at the dyskerin-dyskerin interface compromise the assembly of the 5’ heterotetramer and consequently telomerase assembly, leading to telomerase deficiency (8, 9, 21, 27). A cluster of previously unaccounted disease mutations in hTR, NOP10, NHP2 and dyskerin is located at the dimerization interface between the 5’ H/ACA proteins and the P4/5 linker of hTR (Fig. 5, A and B). In particular, NOP10 R34 and dyskerin R158 directly interact with the P4/5 linker (Fig. 3, F to H). NOP10 R34W has been shown to impair pre-RNP assembly (33). This suggests that these disease mutations may affect the assembly of the 5’ H/ACA proteins by disrupting their interaction between the P4.2 stem at the dimerization interface.

To test the role of dimerization in telomerase assembly, we mutated key residues in H/ACA proteins that are involved in (Fig. 3, F to H; Fig. 5, C and D; figs. S17, S18, A and C). Our telomerase reconstitution was supplemented with either overexpressed 3xFlag-tagged WT or mutant H/ACA proteins. Our telomerase reconstitution relies on assembly of hTR with the endogenous H/ACA proteins (34). Assembly defects can be assessed by comparing the relative incorporation levels of the overexpressed proteins to those of the endogenous proteins (27). We purified telomerase using O-purification on hTR and performed immunoblots against dyskerin or NHP2. Due to being present in vast excess relative to the endogenous proteins, overexpressed WT dyskerin and NHP2 efficiently outcompeted their endogenous counterparts for assembly into telomerase (Fig. 5, C and D). In contrast, three dyskerin mutants (R211D/R212D, R158W/R211A/R212A and R158W/R211D/R212D) and both NHP2 mutants (K121A/R122A and K121D/R122D) are less efficient in competing with endogenous dyskerin and NHP2, respectively, for incorporation into telomerase (Fig. 5, C to F). The dyskerin R158W and R211A/R212A mutants showed slight but significant defects in their abilities to compete with the endogenous dyskerin for telomerase incorporation (Fig. 5, C and E). Thus, our data indicate that these mutant H/ACA proteins are defective in telomerase incorporation. Notably, the level of TERT being assembled with hTR was greatly reduced for the dyskerin triple mutant R158W/R211D/R212D (Fig. 5C). This suggests that disrupted dimerization can compromise hTR assembly with TERT.

To rule out the possibility that the dyskerin and NHP2 mutations selected above may affect the assembly of the 3’ H/ACA proteins, we examined the equivalent residues in the 3’ dyskerin and 3’ NHP2. R158, R211 and R212 in the 3’ dyskerin are not involved in any protein-protein or protein-RNA interactions (figs. S18A; S19, A and B). Although K121 and R122 in the 3’ NHP2 interact with TCAB1 and hTR backbone, respectively, they are not conserved across archaea and eukaryotes (figs. S18C; S19, C and D). These interactions have been suggested to enhance the affinity of the 3’ H/ACA heterotetramer for the 3’ H/ACA hairpin of hTR but are likely not crucial for the assembly of the 3’ hairpin (8, 9, 32). We did not mutate residues R62, K65 and K69 of NHP2 despite their involvement in dimerization (Fig. 3, G and H). These three residues are part of a highly conserved RNA binding surface essential for the interaction of the 3’ NHP2 with the 3’ RNA hairpin (figs. S18C; S19, C and D). Mutating these residues would impact the assembly of the 3’ heterotetramer and thus telomerase assembly, independent of dimerization. Therefore, the telomerase assembly defects of the dyskerin and NHP2 mutants described above would reflect disruption of dimerization (Fig. 5, C to F). Taken together, our structure and biochemical data not only rationalize a cluster of disease mutations in the H/ACA RNP but also suggest that dimerization has a functional role in telomerase assembly.

## The N-terminal RG domains of the two 3’ GAR1 molecules also promote dimerization

**Fig. 6:**
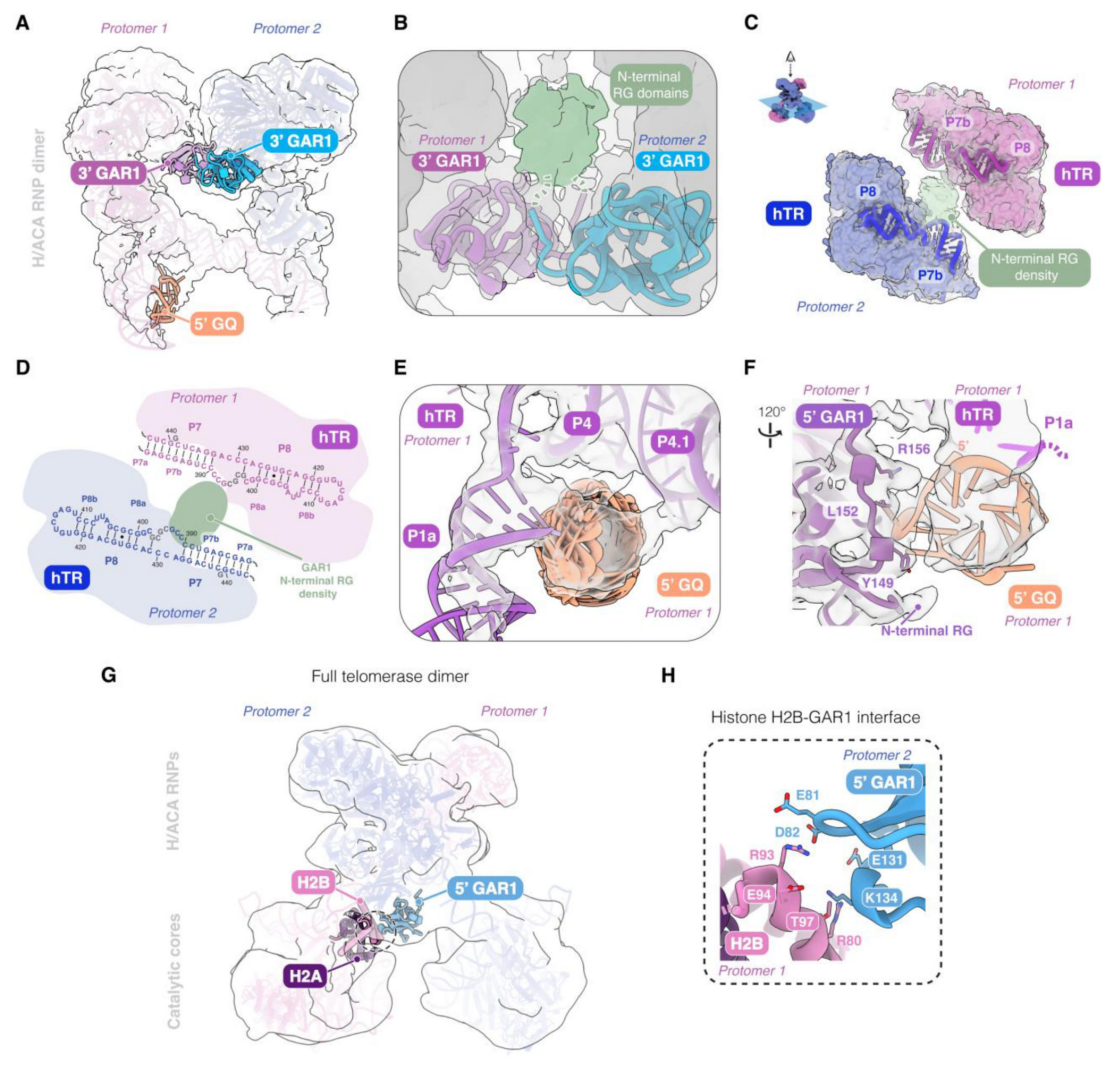
GAR1 role in telomerase dimerization. **(A)** Consensus cryo-EM density of the H/ACA RNP dimer with the 5’ G-quadruplex (GQ), the 3’ GAR1 from protomer 1 and the 3’ GAR1 of protomer 2 fitted into the density. **(B)** Interaction between the 3’ GAR1 subunits of the H/ACA RNP protomers 1 and 2 within the 3.9 Å H/ACA RNP dimer map. Although the density for the N-terminal arginine-glycine (RG) rich domains of both protomers is clear, they do not appear to have defined structures for model building. The dash lines and the green cryo-EM density represent the predicted positioning of the N-terminal RG domains of the 3’ GAR1 subunits. **(C)** The density of the N-terminal RG domains of the two 3’ GAR1 subunits reaching towards the hTR P7b stem of both H/ACA RNP protomers. **(D)** Schematic of the two P7b stems of hTR within the dimer and their contact with the N-terminal RG domains of the 3’ GAR1. **(E)** 3.9 Å cryo-EM H/ACA RNP dimer map with fitted ensemble of the 5’ GQ conformations, modelled by DRRAFTER (26). **(F)** Close-up view of the 5’ GQ interaction with the 5’ GAR1 within protomer 1. The same interaction is formed between the 5’ GQ and the 5’ GAR1 of protomer 2. **(G)** Consensus cryo-EM density of the full telomerase dimer with histone H2A-H2B dimer from protomer 1 and the 5’ GAR1 of protomer 2 fitted into the density. **(H)** A close-up view showing the interface between the histone H2B of protomer 1 and the 5’ GAR1 of protomer 2.

GAR1 is composed of β-barrel domain, flanked on both sides by low complexity arginine-glycine (RG) rich domains (fig. S18B) (9, 10, 27). In our dimer structure, we observe clear density corresponding to the two N-terminal RG domains of the two 3’ GAR1, one from each protomer. Both N-terminal RG domains protrude from the GAR1 β-barrel domain to intertwine with each other (Fig. 6, A to D). However, due to the disordered nature of RG domains, this density does not show high-resolution features sufficient for *de novo* model building (Fig. 6B). RG domains have been implicated in RNA binding and in promoting phase separation in the assembly of membraneless organelles (35). Among the reported telomerase H/ACA RNP structures, only the N-terminal RG domain of the 5’ GAR1 has been partially resolved and found to stabilize an RNA hairpin formed by the 5’ leader sequence of hTR (nucleotides 1–17) (Fig. 1A) (10). In our structure, the intertwined N-terminal RG domains of the two 3’ GAR1 make contacts with the P7b stems of the two hTR (Fig. 6, C and D). Thus, our structure suggests an unexpected contribution for the N-terminal RG domain of the 3’ GAR1 in telomerase dimerization and provides structural evidence affirming its RNA binding capacity.

## The 5’ GAR1 interacts with the 5’ G-quadruplex of hTR and the histone dimer

The 5’ region of hTR (nucleotides 1–31) contains multiple guanosine (G) tracts capable of forming G-quadruplexes, which are four-stranded secondary structures formed by the Hoogsteen base-pairing of four guanine residues (36, 37). By protecting hTR from degradation, G-quadruplex formation is important for telomerase biogenesis and mature hTR accumulation in cells (37–39). In mature telomerase, part of the 5’ G-tracts (nucleotides 18–30) forms the P1a linker (Fig. 1, A and B). The rest of the 5’ region (nucleotides 1–17), also known as the 5’ leader sequence, is assumed to be single-stranded (Fig. 1A) (40). However, it has been demonstrated to form a G-quadruplex *in vitro* (37, 41), but this G-quadruplex structure has not been detected *in vivo* or visualized in any reported telomerase structures (9, 10, 22, 23, 27). Wan et al. observed that this region forms a stem loop instead (10). Moreover, conflicting suggestions have been made regarding whether this 5’ G-quadruplex is mutually exclusive with P1a formation (36, 41).

The stabilization of the P1 linker by dimerization enabled us to resolve the P1a stem and an adjacent density consistent with a 5’ G-quadruplex for the 5’ leader sequence (Materials and methods; fig. S20; Data S2). Thus, our work provides a structural evidence for G-quadruplex formation in human telomerase. Due to the limited local resolution of this region (∼7 Å), we used DRRAFTER to model an ensemble of G-quadruplex structures in the cryo-EM density (Fig. 6E; Data S3). Our structure confirms that the 5’ G-quadruplex structure can co-exist with the P1a stem. This is consistent with a previous finding, which showed a low-level association of the G-quadruplex helicase DHX36 with active telomerase RNP (38). Although we do not observe density for DHX36 in our dimer reconstructions, endogenous DHX36 was consistently detected by mass spectrometry in purified telomerase samples (8, 9).

The 5’ G-quadruplex packs against P4 and P4.1 stems of hTR (Fig. 6E). Consistent with our structure, the interaction between the 5’ leader sequence and P4.1 stem has been previously observed using gel-shift assays (37). The G-quadruplex is also stabilized by the 5’ GAR1 within a single protomer (Fig. 6F), revealing another mode of RNA binding by GAR1. Our observation is supported by a recent study, which demonstrates that GAR1 interacts with G-quadruplex RNAs both in living cells and *in vitro* (42).

Besides interacting with the G-quadruplex, the 5’ GAR1 also contacts the histone H2B in the catalytic core of the other protomer (Fig. 6G). Although the resolution of the dimer consensus map precludes precise determination of these interactions, several residues in histone H2B and GAR1 can potentially form electrostatic interactions at this interface (Fig. 6H). In all previous structures of the telomerase monomer, both the 5’ and 3’ GAR1 associate with the H/ACA RNP solely through well-characterized interactions with dyskerin (9, 10, 22, 23, 27). Hence, our telomerase dimer structure unveils additional interactions between GAR1 and other telomerase components.

## Discussion

Here we uncovered the structure of a low abundant human telomerase dimer by leveraging the large cryo-EM dataset collected on human telomerase. Our work reconciles a longstanding debate regarding the TERT/hTR stoichiometry in human telomerase and provides insights into the molecular interactions crucial for dimerization and the potential roles of the telomerase dimer in telomere maintenance.

The 1.2 MDa human telomerase dimer resolved here is a dimer of the full holoenzyme, rather than a dimer of TERT, hTR and dyskerin as previously suggested (16). The reported 30 Å negative stain EM structure of a 600 kDa human telomerase complex, suggested to be dimeric, strongly resembles the cryo-EM structures of the monomeric telomerase RNP and differs from the telomerase dimer presented in this study (fig. S1F) (8, 9, 17). The previously identified 600 kDa human telomerase complex likely has a monomeric TERT/hTR composition with all the holoenzyme components (14–17). Therefore, our work not only resolves the unambiguity in the composition of the 600 kDa human telomerase complex but also reveals a dimeric telomerase complex, which has not been previously visualized.

Our data present several lines of evidence indicating a role for dimerization in telomerase assembly. First, dimerization is mostly mediated by the H/ACA RNP, which is involved in telomerase biogenesis and accumulation (11, 32). Second, several dyskeratosis congenita disease mutations in the 5’ H/ACA proteins and hTR are located at key dimerization interfaces (Fig. 5, A and B). Third, disruption of dimerization leads to defects in telomere maintenance in cells and incorporation of the H/ACA proteins into telomerase (Fig. 4, C to H; Fig. 5, C to F). We propose that dimerization promotes telomerase assembly by stabilizing the suboptimal binding of the 5’ H/ACA heterotetramer to the 5’ H/ACA hairpin of hTR during assembly (Fig. 3, B to D). Given the presence of all accessory factors, the dimer would represent an assembly intermediate that is close to RNP maturation. This would account for the low abundance of the dimer in our sample and the telomerase activity defects resulting from disrupting dimerization. This is reminiscent of the HIV reverse transcriptase (RT). The homodimer of the catalytic p66 subunit of HIV RT serves as an immediate precursor to the mature p66/p51 heterodimer, in which one p66 protomer is converted into the p51 subunit by proteolytic cleavage (43). Notably, like our telomerase dimer, the p66 homodimer also exhibits enzymatic activity (44, 45).

Although both the P4.2 stem and the P4.2/P5 junction (nucleotides 329 and 330) of hTR are involved in dimerization, disrupting the P4.2/P5 junction did not significantly impact dimer formation (Fig. 4B, fig. S13). Notably, nucleotides 327–329 within this junction are known to participate in triplex formation with the H-box and a region downstream of the mature 3’ end of hTR during early telomerase assembly (46). During this process, the binding of the H/ACA proteins disrupts the triplex, enabling 3’ end processing of hTR and promoting efficient telomerase assembly. The mutations we introduced in the P4.2/P5 junction likely partially destabilize this triplex, potentially enhancing telomerase assembly. This improved assembly could counterbalance the downstream effects of impaired dimerization, which may explain why we did not observe a substantial effect on dimer formation in P4.2/P5 junction mutants.

The interaction between the 5’ GAR1 and the 5’ G-quadruplex of hTR may also play a role in telomerase maturation. GAR1 has been identified as a G-quadruplex ligand sensitizing gene (47). In the absence of GAR1, the G-quadruplex is more susceptible to stabilization by small molecules. This implies that GAR1 interaction might contribute to resolving the G-quadruplex rather than stabilizing it. The unwinding of the G-quadruplex may happen during telomerase maturation, explaining its potential absence in the previous telomerase monomer structure (9).

Residue Lys134 of GAR1, which faces histone H2B in the dimer structure (Fig. 6H), has been reported to be SUMOylated in a previous proteomic study (48). Arginine methylation of the N-terminal RG domain of GAR1 has also been identified (49). Such post-translational modifications would perturb the dimerization interface, potentially providing a mechanism to convert the dimer to the monomer.

Given that the dimer is catalytically active (Fig. 2A), we cannot exclude the possibility that dimerization also has a role in telomere extension. One model suggests that a telomerase dimer, with two active sites, could extend either two telomeres simultaneously or a single telomere cooperatively (15, 28). Our *in vitro* data, which show that the two catalytic cores operate independently, are more consistent with the former mechanism. However, substantial evidence indicates that the catalytic activity of human telomerase requires only a single TERT and hTR (18–20). Although live cell imaging of human telomerase cannot infer its oligomeric state due to the sub-stoichiometric labelling of TERT, the observed diffusion coefficient of TERT-containing particles in cells is consistent with a molecular mass of 600 kDa (50). As discussed earlier, this 600 kDa species likely corresponds to the monomeric holoenzyme. Telomerase holoenzymes from unicellular eukaryotes are also unequivocally monomeric for TERT and TERs (51, 52). Furthermore, the P4.2 stem of hTR, which is critical for dimerization, is highly variable in sequence and length among vertebrates (40). TERs from some fish species lack this stem altogether (53). These observations suggest that dimerization is not a conserved feature of telomerase and, from an evolutionary perspective, would argue against a general role for the dimer in telomere extension.

In conclusion, our work unveils the architectural diversity of human telomerase and provides an invaluable platform for further studies of telomerase modulation, assembly and function in the dimer context. The identification of the dimeric state and its functional role could have broad implications for telomere-related diseases and therapeutic strategies.

## Materials and methods

### Expression of human telomerase

Human telomerase was reconstituted in Expi293F™ cells (ThermoFisher, Cat# A14527) by co-transfection with pcDNA3.1-ZZ-TEV-twin-Strep-SUMO*-TERT and pcDNA3.1-U3-hTR-HDV (8, 32). TEV is the Tobacco Etch Virus protease cleavage site, SUMO* is the SUMOstar protease cleavage site (LifeSensors, Cat# SP4110) and HDV is the hepatitis delta virus ribozyme. Whole-cell extracts were prepared by three hypotonic freeze-thaw cycles, snap-frozen in liquid nitrogen and stored at -70°C until used.

### Reconstitution of human telomerase with DNA and TPP1-POT1-TIN2 (TPT)

Telomerase lysates were incubated with streptavidin agarose resin (Thermo Scientific, Cat# 20361), prebound to a 5’ biotinylated 2’-*O*-methyl oligonucleotide for 3 hours at room temperature. After washing the resin with wash buffer (20 mM HEPES NaOH pH 8.0, 150 mM NaCl, 2 mM MgCl_2_, 0.2 mM EGTA, 10% glycerol, 0.1% IGEPAL CA-630, 1 mM DTT and 0.2 mM PMSF), sample was eluted with a competitor oligonucleotide (sequence: CTAACCCTAACTGATGACAGGTCTAGddC where ddC stands for dideoxycytosine). Next, MagStrepXT resin (IBA LifeSciences, Cat# 2-4090-002) was added to the eluate and incubated for 2 hours at 4 °C. The resulting resin was washed three times with wash buffer and then incubated with 10 μM telomerase DNA primer [either (TTAGGG)_5_ or (TTAGGG)_5_TTA and 100 μM dGpCpp, a non-hydrolyzable analog of dGTP, (Jena Bioscience, Cat# NU-431S)] for 45 minutes at room temperature. After washing, purified TPP1-POT1-TIN2 (TPT) was added to the resin at a final concentration of 0.15 mg mL^-1^ in 1 column volume (CV) of MagStrepXT beads and incubated for 1 hour at 4 °C. TPT was expressed in *S. frugiperda* (Sf9) (Sf9, Oxford Expression Technologies Ltd, Cat# 600100) and purified as previously described (23). Complexes were eluted from the resin by overnight cleavage with SUMOstar protease (LifeSensors, Cat# SP4110) at 4 °C. Fractions were analyzed on the SDS-PAGE gel followed by silver staining (Invitrogen Cat# LC6070).

### Cloning of mutant hTR, TERT, dyskerin and NHP2

Mutant constructs were generated as described previously (23). Briefly, we used NEBaseChanger to design mutagenesis primers (see Table S3). Mutants of pcDNA3.1-U3-hTR-HDV, pcDNA 3.1 ZZ-TEV-twin-Strep-SUMO*-TERTΔPAL, pcDNA3.1-ZZ-TEV-3xFlag-TERTΔPAL, pcDNA3.1-3xFlag-dyskerin (Addgene, Plasmid# 126870), or pcDNA3.1-3xFlag-NHP2 (Addgene, Plasmid# 126871) (21, 32) were generated using NEB Q5 Site-Directed Mutagenesis Kit (NEB, Cat# E0554). Catalytically dead mutants pcDNA3.1-ZZ-TEV-twin-Strep-SUMO*-TERT D712A/D868A/D869A and pcDNA3.1-ZZ-TEV-3xFlag-TERT D712A/D868A/D869A were generated by QuikChange site-directed mutagenesis. SmBiT and LgBiT luciferase fragment constructs were amplified from plasmids kindly provided by Leo James’ laboratory and inserted at the N-terminus of pcDNA3.1-ZZ-TEV-twin Strep-SUMO*-TERT and pcDNA3.1-ZZ-TEV-3xFlag-TERT. Correct mutations and cloned constructs were confirmed by DNA sequencing and plasmid stocks for transfection were prepared using PureLink HiPure plasmid midiprep kit (Invitrogen, Cat# K210005).

### Telomerase activity assays

Telomerase primer extension assays were performed in 50 mM Tris-acetate pH 8.0, 4 mM MgCl_2_, 250 μM dTTP, 250 μM dATP, 5 μM unlabelled dGTP, 0.1 μM α-^32^P-dGTP (3,000 Ci mmol^-1^, 10 mCi mL^-1^) (Hartmann Analytic Gmbh, Cat# FP204), 500 nM (TTAGGG)_5_ and 5 mM DTT. Reactions were carried out at 30 °C for 45 minutes and stopped with 50 mM Tris HCl pH 7.5, 20 mM EDTA and 0.2% SDS. Following nucleic acid extraction with phenol:chloroform:isoamyl alcohol (25:24:1) (Thermo Scientific, Cat# 17908), sample was precipitated using ethanol with a ^32^P-labelled 24 nt-DNA as a recovery control (RC). The primer extension products were run on a 10.5% denaturing polyacrylamide (19:1) TBE gel at 500 V for 2 hours and 15 minutes. The gel was subsequently dried at 80 °C for 60 minutes and exposed to a phosphorimager screen overnight. The screen was scanned by an Amersham Typhoon Biomolecular Imager (Cytiva) and quantified using ImageQuant (Cytiva).

### Expression and purification of the dual-tagged telomerase

Human telomerase with two tags was reconstituted in Expi293F™ cells (ThermoFisher, Cat# A14527) by co-transfection with pcDNA3.1-ZZ-TEV-twin-Strep-SUMO*-TERT, pcDNA3.1-ZZ-TEV-3xFlag-TERT and pcDNA3.1-U3-hTR-HDV. Whole-cell extracts were prepared by three hypotonic freeze-thaw cycles. Telomerase lysates underwent 2’-*O*-methyl oligonucleotide purification to yield oligonucleotide elution (OE) as described above. DYKDDDK Fab-Trap™ Agarose resin (Chromotek, Cat# ffa) was added to the eluate and incubated overnight at 4 °C. After washing with wash buffer, the sample was eluted off the beads with 0.5 mg mL^-1^ Flag peptide (ThermoScientific, Cat# A36805). Next, the Flag eluate was incubated with MagStrepXT resin (IBA LifeSciences, Cat# 2-4090-002) for 2 hours at 4 °C. The resulting resin was washed three times with wash buffer. The sample was eluted from the resin by overnight cleavage with SUMOstar protease (LifeSensors, Cat# SP4110) at 4 °C. Fractions were analyzed by negative staining and telomerase activity assays. Purifications of the dual-tagged WT dimer (168 mL of input lysate from 1 L of cell suspension, fig. S5A), the ΔPAL dimer (168 mL of input lysate from 1.2 L of cell suspension, fig. S10E), and the dimer with one copy of WT TERT and one copy of the catalytically inactive TERT (196 mL of input lysate from 1.5 L of cell suspension, fig. S11A) were performed using the method described in this section.

### Expression and purification of single-tagged human telomerase with the P4.2 switch and P4.2 comp. hTR mutants

Human telomerase was expressed in Expi293F™ cells (ThermoFisher, Cat# A14527) by co-transfection with pcDNA3.1-ZZ-TEV-twin-Strep-SUMO*-TERT and pcDNA3.1-U3-hTR-HDV harboring either the P4.2 switch (P4.2 switch) or compensatory (P4.2 comp.) mutations (see Table S3). Cell lysates were prepared by three hypotonic freeze-thaw cycles, snap-frozen in liquid nitrogen and stored at -70 °C until used. Lysates (84 mL from 500 mL of cell suspension) were subjected to 2’-*O*-methyl oligonucleotide purification by incubation with streptavidin agarose resin (Thermo Scientific, Cat# 20361), prebound to a 5’ biotinylated 2’-*O*-methyl oligonucleotide, as described above. MagStrepXT resin (IBA LifeSciences, Cat# 2-4090-002) was added to the oligo purification eluate and incubated for 2 hours at 4 °C. The resin was washed three times with wash buffer. Telomerase was eluted by overnight cleavage with SUMOstar protease (LifeSensors, Cat# SP4110) at 4 °C. The final elution sample was analyzed by immunoblotting, activity assay, DMS chemical probing and negative stain EM (figs. S15 and S16).

### Split nano-luciferase assay

HEK293T cells (ATCC, Cat# CRL-3216, RRID:CVCL_0063) were grown in DMEM with GlutaMax (ThermoFisher, Cat# 10566016), supplemented with 10% FBS, 100 μg/mL streptomycin, and 100 unit/mL penicillin. Cells were seeded at an approximate density of 20,000 cells per well in a 96 well plate (Greiner, Cat# 655098). After 24 hours, cells were co-transfected with pcDNA3.1-U3-hTR-HDV and two pcDNA3.1-TERT plasmids, one containing the SmBiT and the other containing the LgBiT with a total of 300 ng DNA used per well (fig. S4). In each transfection combination, one TERT was Flag-tagged, and the other was twin-Strep-tagged. Transfection was performed with Lipofectamine 3000 (ThermoFisher, Cat# L3000001) in OptiMem™ reduced serum medium (Gibco, Cat# 31985062) for 24 hours. The Nano-Glo® live cell reagent (Promega, Cat# N2011) was added to the wells, and bioluminescence was measured with the Glomax® Discover plate reader (Promega, Cat# GM3000). Bioluminescence is obtained when the LgBiT and SmBiT fragments of nano-luciferase are in close proximity. Bioluminescence values were normalized to the untransfected control (fig. S4B). Transfection and assays were performed in triplicate.

### Glycerol gradient centrifugation

Human telomerase was reconstituted in Expi293F™ cells (ThermoFisher, Cat# A14527) by co-transfection of pcDNA3.1-ZZ-TEV-3xFlag-TERT, pcDNA3.1-ZZ-TEV-twin-Strep-SUMO*-TERT, and pcDNA3.1-U3-hTR-HDV for 48 hours. Cell extract was prepared through three cycles of hypotonic freeze-thaw lysis, snap-frozen in liquid nitrogen, and stored at -70°C until use. The resulting lysate (112 mL from 630 mL of cell suspension) was subjected to 2’-*O*-methyl oligonucleotide purification as described above. The eluate from this purification was incubated with pre-washed MagStrepXT resin for 2 hours at 4 °C. Telomerase was eluted at 4 °C by overnight cleavage of the ZZ-twin-Strep tag with SUMOstar protease (LifeSensors, Cat# SP4110) at 4 °C.

The eluate (20 μL) from the Strep-Tactin purification was then applied onto a 200-μL 10-30% glycerol gradient (Beckman Coulter, Cat# 343775), pre-equilibrated overnight at 4 °C. The sample was spun at 4 °C and 55,000 rpm for 90 minutes using a TLS55 rotor (Beckman Coulter). Sample was fractionated into 20 μL aliquots. Fractions were analyzed by telomerase activity assays and immunoblotting (fig. S12). For immunoblotting, a 4-12% Bis-Tris NuPAGE gel was used to resolve the fractions; and anti-TERT (1:1000, Abcam, Cat# 32020, lot 1041820-24) was used as primary antibody. Goat anti-rabbit Alexa-Fluor 680 (1:5,000, Abcam, Cat# ab175773, lot GR222353-8) was used as a secondary antibody.

Total TERT levels for each glycerol gradient fraction were calculated for each fraction using ImageJ. The activity in each fraction was quantified with ImageQuant (Cytiva) and normalized against the signal of the recovery control (RC). This allowed the overall activity per TERT to be obtained. These experiments were not performed in triplicates due to the discontinuation of the TERT antibody from Abcam (Cat# 32020, lot 1041820-24). An alternative TERT antibody from Proteintech (Cat# 27586-1-AP, lot 00132796) did not give quantifiable signals for TERT.

Parallel gradients were also run for gel filtration standards (1.8 mg/mL, Bio-Rad, Cat# 1511901) and the anaphase promoting complex (0.5 mg/mL, a kind gift from J. Yang and D. Barford) (fig. S12B).

### Preparation of dual-tagged telomerase with P4.2 stem and P4.2/P5 junction hTR mutants

To test the contribution of hTR in telomerase dimerization, Expi293F™ (ThermoFisher, Cat# A14527) cells were co-transfected with pcDNA 3.1-ZZ-TEV-twin Strep-SUMO*-TERT, pcDNA 3.1-ZZ-TEV-3xFlag-TERT and WT or mutant pcDNA 3.1-U3-hTR-HDV (Fig. 4, A and B). Cells were harvested after 48 hours and lysates were prepared by three freeze-thaw cycles. For each reconstitution, we used 14 mL of input lysate from 150 mL of cell suspension. Lysates were subjected to oligonucleotide purification, as described above, to yield oligonucleotide elution (OE). OE was incubated with MagStrepXT resin (IBA LifeSciences, Cat# 2-4090-002) for 2 hours at 4 °C. The resin was washed with wash buffer three times (see above) and telomerase was cleaved off the resin using SUMOstar protease (LifeSensors, Cat# SP4110) overnight at 4 °C. Elution fractions were analyzed by immunoblotting against Flag-tag antibody (1:1000, Proteintech, Cat# 66008-4-Ig) (see below). Telomerase activity assays, immunoblotting, and northern blotting were also performed for the crude lysates (fig. S13). Expression, lysis and purification for all samples were performed in triplicates.

### Preparation of dyskerin and NHP2 mutants

To test dyskerin and NHP2 contribution in telomerase dimerization, Expi293F™ (ThermoFisher, Cat# A14527) cells were co-transfected with pcDNA3.1-ZZ-TEV-twin Strep-SUMO*-TERT, pcDNA3.1-U3-hTR-HDV and pcDNA 3.1 with WT or mutant 3xFlag-tagged dyskerin or NHP2 construct. Cells were harvested after 48 hours and lysates were prepared by three freeze-thaw cycles. For each reconstitution, we used 14 mL of input lysate from 100 mL of cell suspension. Lysates were subjected to oligonucleotide purification, as described above, to yield oligonucleotide elution (OE). After normalizing the final volume of oligonucleotide elution (OE), samples were analyzed by immunoblotting to compare the incorporation of overexpressed 3xFlag-dyskerin or 3xFlag-NHP2 into telomerase (Fig. 5, C to F; fig. S17). Immunoblotting was also performed for the crude lysates. Expression, lysis and purification were performed in triplicates for all WT and mutant dyskerin and NHP2.

### Immunoblotting

Fractions of dual-tagged WT telomerase were resolved on a 3-8% Tris-Acetate NuPAGE gel (Invitrogen, Cat# EA0375PK2) in Tris-Acetate buffer and transferred onto a nitrocellulose membrane. The membrane was incubated for 1 hour at room temperature with 5% non-fat milk in PBS supplemented with 0.1% Tween-20 (PBST) before an overnight incubation at 4 °C with anti-Flag (1:1000, Proteintech, Cat# 66008-4-Ug, lot 10027647, 1:1000). Following the washing, the membrane was incubated with goat anti-mouse Alexa-Fluor 680 secondary antibody (1:5000, Abcam, ab175775, lot GR3273649-2, 1:5000) in PBST for 1 hour. Scanning of the membrane was performed on a Li-COR Odyssey Imager and the quantification was performed using ImageJ.

For the dyskerin and NHP2 mutant telomerase, the lysates and elution fractions from the oligo purification (OE) were resolved on a 4-12% Bis-Tris NuPAGE gel (Invitrogen, Cat# NP0321BOX), then transferred onto a nitrocellulose membrane and incubated with 5% non-fat milk in PBST for 1 hour. The membrane was first incubated with a primary antibody overnight at 4 °C (1:1000 rabbit anti-dyskerin, Santa Cruz Biotechnology, Cat# sc-48794, lot E0214, or 1:20000 mouse anti-alpha tubulin, Proteintech, Cat# 66031-1-Ig, lot 10004185; or 1:1000 rabbit anti-NHP2, Proteintech, Cat# 15128-1-AP, lot 00059015).

For the dual-tagged ΔPAL dimer and catalytically inactive telomerase samples, 4-12% Bis-Tris NuPAGE gels were used to resolve lysates, Flag elution and Strep-Tactin elution fractions. Anti-TERT (1:1000, Abcam, Cat# 32020, lot 1041820-24) was used as a primary antibody for the dual-tagged ΔPAL telomerase dimer experiments. During this study, the TERT antibody was discontinued by Abcam. For the catalytically inactive telomerase samples, we used rabbit anti-TERT from Proteintech (1:1000, Cat# 27586-1-AP, lot 00132796), which gives lower signals than the Abcam TERT antibody, and rabbit TCAB1 antibody (1:1000, Proteintech, Cat# 14761-1-AP, lot 60343).

Membranes were washed three times and incubated with a secondary antibody (1:5000 goat anti-rabbit Alexa-Fluor 680, Abcam, Cat# ab175773, lot GR222353-8; or 1:5000 goat anti-mouse Alexa-Fluor 680, Abcam, Cat# ab175775, lot GR3273649-2) before being washed again and imaged on a Li-COR Odyssey Imager. All immunoblots were performed in triplicates, and quantification was done in ImageJ.

### Northern blotting

Total RNA was extracted from the crude lysates of WT hTR and hTR mutants, co-expressed with ZZ-TEV-3xFlag-TERT and ZZ-TEV-twin-Strep-SUMO*-TERT, using TRIzol (ThermoFisher, Cat# 15596026). The purified RNA samples were resolved on a 5% formamide polyacrylamide (19:1) TBE gel and then transferred onto a Hybond N+ nylon membrane (GE Healthcare, Cat# RPN303). The membrane was cross-linked using a Stratalinker UV crosslinker for 2 minutes. Following a 20 minute incubation with Church’s buffer (0.5 M phosphate buffer pH 7.2, 1% BSA, 1 mM EDTA, and 7% SDS) supplemented with 15% formamide at 50 °C, the membrane was incubated with ^32^P-end labelled DNA primers probes specific for the CR4/5 and PK/t domains of hTR overnight at 50 °C. The membrane was washed twice with 4x SSC and 0.1% SDS, and twice with 2x SSC and 0.1% SDS. The dried membrane was exposed to a phosphorimager screen overnight and imaged on an Amersham Typhoon Biomolecular Imager (Cytiva).

### Lentivirus plasmid transfection in HEK293T cells

HEK293T cells (ATCC, Cat# CRL-3216, RRID:CVCL_0063) were cultured in DMEM with GlutaMax (ThermoFisher), supplemented with 10% FBS, 100 μg/mL streptomycin, and 100 unit/mL penicillin. To generate lentivirus, HEK293T cells were transfected with the packaging plasmid (psPAX2), the envelope plasmid (pMD2.G), and transgene constructs expressing hTR mutants, which were cloned into the DUET011 backbone. The WT transgene construct was a kind gift from K. Collins and X. Zhang (University of California, Berkeley) (31). Following a 24-hour incubation, the media was replaced, and cells were allowed to grow for additional 24 hours. After this incubation period, the supernatant containing viral particles was filtered using a 0.22 μm filter, aliquoted, and stored at -70 °C until further use.

### Lentivirus transduction of HCT116 hTR knockout (KO) cells

HCT116 hTR KO cells were a kind gift from K. Collins and X. Zhang (University of California, Berkeley). To transduce HCT116 hTR KO cells, 2 mL of lentivirus was thawed and combined with 8 mL of pre-warmed DMEM with GlutaMax (GibcoTM, Cat# 10566016) supplemented with 10% FBS, 100 μg/mL streptomycin, and 100 unit/mL penicillin. The lentivirus/DMEM mixture was supplemented with 6 μg/mL of polybrene to enhance transduction efficiency and was then added to the HCT116 hTR KO cells. After 24 hours, the media was replaced. The media was replaced again 72 hours post-transduction, and the cells were selected with hygromycin B at 300 μg/mL for 7 days. Following the selection period, the cells were passaged using DMEM with GlutaMax (GibcoTM, Cat# 10566016) supplemented with 10% FBS, 100 μg/mL streptomycin, and 100 unit/mL penicillin.

### Extraction of genomic DNA from HCT116 cells

HCT116 cell pellets (∼50 μL) were suspended in 500 μL of RIPA buffer (SERVA, Cat# 39244.1), to which 6.25 μL of RNase A (ThermoFisher, Cat #12091021) (20 mg/mL) was added, followed by an incubation at 37 °C for 30 minutes. After this incubation period, 10 μL of proteinase K (20 mg/mL) was introduced to the reaction, which was then incubated overnight at 50 °C. The reaction mixture was extracted with phenol:chloroform:isoamyl alcohol (Thermo Scientific, Cat# 17908) and precipitated using isopropanol. The resulting DNA pellet was washed twice with 75% ethanol and air-dried before resuspension in TE buffer (10 mM Tris HCl pH 7.4 and 1 mM EDTA pH 7.4) with constant rotation overnight.

### Telomere restriction fragment assay

Between three to ten μg of purified genomic DNA was digested with FastDigest AluI (ThermoFisher, Cat# FD0014) and MboI (ThermoFisher, Cat# FD0814) restriction enzymes at 37 °C overnight. The digested DNA fragments were separated on a 0.7% agarose gel in 1x TAE buffer for approximately 23 hours at 35 V. The gel was vacuum dried for 1 hour at 50 °C. The dried gel was denatured using denaturation buffer (0.5 M NaOH, 1.5 M NaCl) for 30 minutes at 50 °C. After denaturation, the gel was washed with 4x SSC buffer supplemented with 0.1% SDS for 10 minutes and then blocked with Church’s buffer for 30 minutes at 50 °C. Following the blocking step, ^32^P-end-labeled telomeric repeat probes ((TTAGGG)_4_, (CCCTAA)_4_) and a 1 kb plus DNA ladder (ThermoFisher, Cat #10780718) probe were added. The probes were hybridized overnight at 50 °C. The gel was washed 3 times with 4x SSC and 0.1% SDS at 40 °C. The gel was then exposed to a phosphorimager screen for at least 24 hours before being imaged using an Amersham Typhoon Biomolecular Imager (Cytiva).

### Negative staining EM sample preparation

Telomerase sample of interest was prepared without crosslinker and applied (3 μL per grid) on 400 mesh copper grids (Electron Microscopy Sciences, Cat# G400-Cu) with a homemade amorphous carbon film on nitrocellulose. Prior to sample application, negative stain grids were glow discharged using a Sputter coater discharger (model Edwards S150B). Sample was incubated on the grid for 90 seconds before undergoing 1 minute incubation with 2% w/v uranyl formate (UF) by gentle agitation. Automatic data collection was done using Tecnai F20 transmission electron microscope (200 kV) with a Falcon 3 direct electron detector in linear mode. Collection was performed in EPU software and at a physical pixel size of 2.02 Å per pixel, with a total electron dose of 41.7 electrons per Å^2^ over a total exposure time of 2 seconds. We collected 4,664 micrographs for the dual-tagged WT telomerase dimer (fig. S5, C to F); 1,419 micrographs for the dual-tagged ΔPAL dimer (fig. S10, G to I); 1,257 micrographs for the single-tagged P4.2 switch mutant (fig. S16, A and B); and 1,102 micrographs for the single-tagged P4.2 comp. mutant (fig. S16, C and D).

### Negative stain EM data processing

Data processing was performed in RELION 5.0 (54, 55). CTFFIND4.1 was used to estimate contrast transfer function (CTF) parameters of the micrographs (56). Data for each complex were processed separately as detailed below.

#### The dual-tagged WT telomerase dimer

A total of 149,089 particles were first picked with Laplacian-of-Gaussian (LoG) blob detection in RELION (57). The particles were binned by 4, extracted with a box of 60^2^ pixel and subjected to multiple rounds of 2D classification. We then unbinned a subset of 20,105 particles for generating 2D references to improve particle picking. Four 2D references from 863 particles were selected as 2D references for reference-based picking. The second round of picking yielded 50,123 particles, which were binned by 4, extracted with a 60^2^ pixel box and subjected to 25 rounds of 3D classification with 8 classes and a regularization parameter *T* of 4. We used the cryo-EM reconstruction of the full dimer as an initial model for this 3D classification. Six of the classes resembled the dimer particles observed in cryo-EM data and were thus pooled for 2D classification. Representative 2D class averages are shown in fig. S5E.

#### The dual-tagged ΔPAL dimer

To avoid model bias, we picked particles using 2D references of the monomeric telomerase RNP. A total of 110,700 particles were picked, binned by 4, extracted with a 60^2^ pixel box and subjected to several rounds of 2D classification. Representative 2D class averages are shown in fig. S10H. Although the particles were picked with 2D references of the telomerase monomer, the majority resembled the dimeric particles.

#### The P4.2 switch mutant

To generate 2D references for picking, we picked 207,603 particles using LoG blob detection in RELION. Particles were binned by 4, and extracted with a 60^2^ pixel box. After several rounds of 2D classification, we unbinned a subset of 4,516 particles for 2D classification. Four 2D references from 1,493 particles were selected as 2D references for reference-based picking. A total of 230,944 particles were re-picked using these 2D references, binned by 4, extracted with a 60^2^ pixel box and subjected to several rounds of 2D classification. 2D classes of monomeric telomerase RNP from 14,547 particles are shown in fig. S16B.

#### The P4.2 comp. mutant

A total of 278,349 particles were picked using the 2D references from the P4.2 switch mutant, binned by 4 and extracted with a 60^2^ pixel box. After several rounds of 2D classification, we obtained 2D class averages of monomeric telomerase RNP from 94,006 particles, that are shown in fig. S16D.

### DMS chemical probing and data analysis

RNA structures in telomerase complexes reconstituted with WT hTR, and P4.2 switch and comp. hTR mutants were obtained using DMS-MaPseq (58). For DMS probing, the RNA was allowed to react with DMS at a final concentration of 1% for 5 minutes at 37 °C. Dithiothreitol (DTT) was added to a final concentration of 0.5 M to quench the reaction. To remove the protein components after DMS probing, we added an equal volume of triazole and chloroform, mixed well, and centrifuged the sample for 15 minutes. The supernatant was mixed with an equal volume of 100% ethanol. RNA Clean & Concentrator Kits (Zymo Research, Cat #R1016) were used for RNA purification following the manufacturer’s protocol. Reverse transcription was done using SuperScript™ II Reverse Transcriptase (ThermoFisher, Cat #18064014) at 42 °C for 90 minutes (59). To remove the SuperScript™ II enzyme, an equal volume of phenol:chloroform:isoamyl alcohol (25:24:1) was added to the reaction mixture, followed by centrifugation for 15 minutes. The aqueous phase was collected and subjected to further purification using a MicroSpin S-200 HR column (Cytiva, Cat #27512001) in accordance with the manufacturer’s instructions.

PCR reactions with 10 cycles were performed with the reverse primer (5’-GCGGGGACTCGCTCCGTTCCTCTT-3’) and the forward primer (5’-GGGTTGCGGAGGGTGGGCCTG-3’) using KOD Xtreme Hot Start DNA Polymerase (Novagen, Cat #71975). Two independent biological replicates for each sample were generated. The purified DNA samples were subjected to Nanopore library construction by following the manufacturer’s instructions for the native barcoding kit (NBD-24). Raw reads from DMS probing data were base called and demultiplexed using Dorado (https://github.com/nanoporetech/dorado). The resulting reads were mapped using minimap2 (60) with the following parameters: minimap2 -t 8 -ax splice --secondary=no -G 12000. The mutation rate at each nucleotide was calculated by dividing the number of mutations by the number of reads at that location to generate the overall DMS reactivity profiles (fig. S15E). DMS reactivity profiles were then normalized by scaling each reactivity value by the median of the top 10% most reactive positions (61).

### Cryo-EM sample preparation and data collection

Purified telomerase-DNA-TPT sample was crosslinked with bis(sulfosuccinimidyl)suberate (BS3) crosslinker (ThermoFisher, Cat# A39266) on ice for 1 hour at 0.5 mM final BS3 concentration. The reaction was then stopped with quench buffer (200 mM Tris pH 8.0, 150 mM NaCl, 2 mM MgCl_2_, 0.05% IGEPAL CA-630 and 1 mM DTT) and buffer-exchanged into cryo-EM buffer (20 mM HEPES NaOH pH 8.0, 150 mM NaCl, 2 mM MgCl_2_, 0.05% IGEPAL CA-630, 1.0% trehalose and 1 mM DTT) before cryo-EM grid preparation. Crosslinked telomerase-DNA-TPT complex was applied (3 μL per grid) onto a C-flat-T-50 4/2 grids (Protochips, Cat# CF-4/2-4Cu-T-50). The grids were pre-coated inhouse with a 5-6 nm thick layer of homemade continuous carbon film and glow-discharged using a Sputter coater discharger (model Edwards S150B). Following a 2 minute incubation, the grids were blotted for 5 to 6 seconds at 4 °C, and 100% humidity and vitrified in liquid ethane using an FEI Vitrobot MK IV. Data collection was performed on ThermoFisher Titan Krios transmission electron microscope operated at 300 kV and equipped with a Gatan K3 direct electron detector camera and a GIF Quantum energy filter using 20 eV slit width. EPU software (ThermoFisher) was used for automatic collection in counting mode. The physical pixel size of 1.059 Å was used with a total electron dose of approximately 48 electrons per Å^2^ over a total exposure time of 2.5 seconds, and a defocus range of -0.8 to 2.4 μm. Doses were fractionated into 48 movie frames. A total of 66,992 movies were collected in four separate sessions.

### Cryo-EM data processing

Data processing was performed using RELION 4.0, RELION 5.0 and CryoSPARC v4.1.2 (fig. S2) (54, 57, 62). Movie frames were gain-corrected and drift-corrected, dose-weighted and summed into single micrographs using motion-correction program implemented within RELION 4.0 (57). All 3D classification after the signal subtraction steps (except for those without alignments) and refinements in RELION were performed with the Blush algorithm (55). CTF parameters were estimated for the motion-corrected micrographs using CTFFIND-4.1 (56). A total of 66,992 movies were corrected as described above (dataset 1 (23,681 micrographs), dataset 2 (22,330 micrographs), dataset 3 (10,832 micrographs), dataset 4 (10,149 micrographs)).

3D refinements were performed using independent data half-sets. Reported resolutions are estimated at the gold-standard Fourier shell correlation (FSC) = 0.143 criterion between the two half-maps. FSCs were calculated using a soft mask. We also corrected for the modulation transfer function of the detector and applied a B-factor sharpening, as determined by RELION or a user-defined value.

### Cryo-EM data processing of the telomerase dimer

For dataset 1, particle picking was performed using a reference-based autopicking. We first used 2D references generated from previous work (23) and then generated new references via multiple rounds of 2D classification. Particles were picked again using the new 2D references. Picked particles from dataset 1 were binned by 8 and extracted using a box size of 56^2^ pixel. Two rounds of 2D and 3D classifications were done together with the dataset 2 using a consensus monomeric telomerase map as a reference (map not deposited) (23). During the classification, a subset of telomerase dimer particles was obtained and refined to yield a 11.8 Å reconstruction, which was used as an initial dimer reference for subsequent classification.

All binned particles from dataset 1 were subjected to an initial round of 3D classification using a dimer map as a reference. We then performed a second round of 3D classification using an angular sampling of 15° for 25 iterations and 7.5° for another 25 iterations, regularization parameter *T* of 4 and a dimer map as a reference. The best dimer classes were selected for a reference-free 2D classification, from which a total of 598,022 particles were selected, unbinned and imported into CryoSPARC.

For dataset 2, particle picking was done using a Topaz trained model (63) implemented within RELION 4.0. Selected particles with figure-of-merit (FOM) cut-off values of -1.5 were binned by 8 and extracted using a box size of 56^2^ pixel. Particles from dataset 1 and dataset 2 were subjected to two rounds of 2D and 3D classifications to obtain a telomerase dimer map as described above. After obtaining the dimer reference, all binned particles from dataset 2 underwent two rounds of 3D classification with a dimer map as a reference. The best 3D classes of telomerase dimer were selected for a reference-free 2D classification to further remove bad particles. A total of 824,473 particles were selected, unbinned and imported into CryoSPARC.

For dataset 3 and dataset 4, particle picking was performed using a reference-based autopicking with 2D references of the monomeric telomerase obtained from dataset 1. The picked particles were binned by 8 and extracted using a box size of 56^2^ pixel. The binned particles underwent a first round of 3D classification to remove junk particles, following a second round of 3D classification using an angular sampling of 7.5° and regularization parameter *T* of 4. The second round of 3D classification used a dimer map as a reference as described for dataset 1 and dataset 2. The best dimer 3D classes were selected for a reference-free 2D classification, from which a total of 239,908 (dataset 3) and a total of 208,882 (dataset 4) particles were selected, unbinned and imported into CryoSPARC.

In CryoSPARC, a subset of particles (193,181 particles) from datasets 1, 2, 3 and 4 was used to generate six *Ab-Initio* reconstruction maps with C2 symmetry. The whole particle set was then classified into six volumes using the heterogenous refinement algorithm with C2 symmetry and maps from the prior *Ab-Initio* reconstruction step as initial models (62). The class with the best features containing 505,039 particles was subjected to the non-uniform refinement (64) to yield a reconstruction of telomerase dimer at 6.2 Å resolution. The particles were exported as a particle stack using PyEM (65) for further processing in RELION 4.0 and RELION 5.0. To investigate the heterogeneity of the full telomerase dimer, we performed 3D variability analysis (3DVA) on the 6.2 Å dimer map in CryoSPARC (66). The 3DVA results showing the global heterogeneity are presented in Movie S1.

The exported particle stack was refined in RELION to give a reconstruction at 7.1 Å resolution without any symmetry applied. The map was aligned to the C2 symmetry axis, symmetrized in RELION, and used as a reference for a 3D refinement with C2 symmetry. This yielded a reconstruction of the full telomerase dimer at 4.4 Å resolution. This reconstruction was well-aligned on the H/ACA RNPs but poorly aligned on the catalytic cores. Thus, we use this refinement for the subsequent signal subtraction procedures but not for model fitting or deposition to the EMDB. Particles from this refinement were subjected to C2 symmetry expansion.

### Cryo-EM data processing of the individual H/ACA RNP protomer

To improve the resolution of the individual H/ACA RNP lobes and regions responsible for dimerization, we used the 4.4 Å dimer reconstruction to perform signal subtraction with recentering on the symmetry expanded particles. The box size of the signal subtracted particles was downsized to 280^2^ pixel to speed up computation. The signal subtracted particles were refined and then classified into 4 classes using a local classification with 5° local angular search steps. One class with 322,295 particles had well-defined high-resolution features and was further refined to 3.0 Å resolution. To resolve the dimerization interface between the 5’ H/ACA heterotetramer of one H/ACA RNP and the P4/5 linker of hTR of the second H/ACA RNP, a mask that included only these regions was used for alignment-free focused 3D classification with a regularization parameter *T* of 10. A subset of 260,466 particles, which showed the best resolved P4/5 linker region, was refined to 3.0 Å resolution. We then performed CTF refinement (beam tilt, trefoil, and 4^th^ order aberrations, anisotropic magnification, per-particle defocus and per-micrograph astigmatism) (67), followed by a 3D refinement. The resulting particles yielded a final 3.0 Å resolution map of an individual H/ACA RNP with the bound P4/P5 linker from the other protomer. Although the resolution of the map after CTF refinement did not improve, the quality of the map was improved. This reconstruction was used for model building, refinement and validation of telomerase H/ACA RNP protomer.

### Cryo-EM data processing of the H/ACA RNP dimer

To analyze the dimerization interface between the two H/ACA RNPs, we performed signal subtraction with recentering on the non-symmetry expanded particles. We generated a mask around the two H/ACA RNPs to remove the signal from both catalytic cores. The box size of the signal subtracted particles was downsized to 280^2^ pixel to speed up computation. The signal-subtracted particles were refined to 4.0 Å resolution with C2 symmetry using a C2 symmetry axis-aligned and symmetrized dimer reference. We then performed a 3D classification with local searches and a C2 symmetry relaxation. A subset of 130,095 particles was used for 3D refinement (4.1 Å resolution), followed by alignment-free 3D classification with regularization parameter *T* of 8 and a mask around the 3’ GAR1-dimerization interface. A subset of 88,419 particles, which showed the most complete density for both 3’ GAR1 subunits, was refined to 3.9 Å resolution. This reconstruction was used for model building, refinement and validation of the telomerase H/ACA RNP dimer.

### Cryo-EM data processing of telomerase catalytic cores

To analyze the catalytic core, we used the 4.4 Å dimer reconstruction to perform signal subtraction around one telomerase catalytic core with recentering on the symmetry expanded particles. The box size of the signal subtracted particles was downsized to 280^2^ pixel to speed up computation. The signal subtracted particles were refined and then classified into 4 classes using 25 iterations with angular sampling of 1.8° and regularization parameter *T* of 4. The best class with 273,757 particles had well-defined features and was further refined to 3.4 Å resolution. We then performed CTF refinement (beam tilt, trefoil, and 4^th^ order aberrations, anisotropic magnification, per-particle defocus and per-micrograph astigmatism) (67), followed by a 3D refinement. This yielded a final map of individual telomerase catalytic core at 3.3 Å resolution. This reconstruction was used for model building, refinement and validation of telomerase catalytic core monomer (C2).

Additionally, we wanted to determine if the DNA is bound in both catalytic cores of the telomerase dimer. To resolve the two catalytic cores independently, signal subtraction was performed around each catalytic core in a 7.1 Å dimer map, with recentering on the non-symmetry expanded particles. Subsequently, a 3D classification into 4 classes using 25 iterations with angular sampling of 3.7° was carried out, resulting in one class with high-resolution features. For catalytic core from protomer 1 (catalytic core 1), we selected a subset of 138,966 particles. For catalytic core from protomer 2 (catalytic core 2), a subset of 133,259 particles was selected. We refined these two subsets, followed by CTF refinement (beam tilt, trefoil, and 4^th^ order aberrations, anisotropic magnification, per-particle defocus and per micrograph astigmatism), and final 3D refinement. This approach yielded reconstructions at 3.8 Å resolution for catalytic core 1 and at 3.6 Å resolution for catalytic core 2.

Finally, to confirm the presence of DNA in both catalytic cores within a single telomerase dimer particle, we examined the particle subsets used to obtain the maps of telomerase catalytic cores 1 and 2. We searched for dimer particles present in both subsets using https://github.com/sami-chaaban/starparser. We then performed local 3D refinement on the shared particles using 1.8° angular sampling. This approach yielded maps of telomerase catalytic cores 1 and 2 at 4.1 Å and 3.9 Å resolution, respectively, with both reconstructions showing DNA density in the active site.

### Model building and refinement

To facilitate model building, all maps were converted into MTZ format using REFMAC5.8 (68, 69) to allow map blurring and sharpening in COOT (70). The published models of the catalytic core bound to TPP1 (PDB 7QXA) and H/ACA RNP (PDB 8OUE) (23, 27) were used as initial models for model building. These models were fitted into the corresponding maps by rigid-body fitting in COOT. Manual adjustments of the models were performed in COOT to improve the map fit. The P4.2/5 and P1 linkers of hTR were first manually built in COOT using the 3.9 Å H/ACA RNP dimer map. The resulting model was used as a starting model for DRRAFTER (26). After DRRAFTER, the RNA geometry was further improved using ERRASER2.0 (71) and manual adjustments in COOT.

Model refinement was performed in reciprocal space using Servalcat and REFMAC5.8 (68, 72) with protein secondary structure restraints and nucleic acid restraints calculated using PROSMART and LIBG, respectively (69, 73). Phenix 1.20 was used to calculate model-versus-map FSCs and EMRinger scores (74). Geometries were assessed using the MolProbity server (http://molprobity.biochem.duke.edu/) (75). Tables S1 and S2 provide a summary of the refined models.

The refined models of the H/ACA RNP dimer and the catalytic cores were rigid-body fitted into the 6.2 Å consensus dimer map. The resulting models were used as an input for DRRAFTER modeling of the full hTR within the dimer. Due to the limited resolution of the map, no model refinement was performed. Instead, the top 10 DRRAFTER models of hTR and fitted models of the protein subunits of both the catalytic core and the H/ACA RNP are provided in a Pymol session (Data S1). The G-quadruplex structure in the 5’ region of hTR was modelled by DRRAFTER; and the ensemble of the structures with the lowest energy are included in a Pymol session (Data S3).

### DRRAFTER modeling of RNA in telomerase dimer

DRRAFTER was used for the modeling of hTR regions with higher disorder (26). In each case, 2,000–5,000 models were obtained, and a set of the lowest energy models were selected to represent the ensemble of conformations. The secondary structure was enforced, with ideal A-form helices used as template as well as the parallel conformation of the G-quadruplex for residues 1–17 of hTR (76). For the H/ACA RNP dimer region, the cryo-EM map around the RNA of interest (residues 1–31, 195–247 and 322–370) was isolated by creating a 12 Å zoning around the RNA and removing segments associated with modeled protein using Segger in ChimeraX (77). Within the H/ACA RNP dimer, only one of the RNA chains was modeled, assuming the other is a symmetry copy. Four sets of ensemble models of the H/ACA RNP dimer region were created. One was created using 15 Å restraints from the initial placed positions, and another with 30 Å restraints. Little difference was observed between these two ensembles, suggesting confidence in the global placement of helices. Furthermore, observing density between P4.1 stem of hTR and the proposed G-quadruplex (GQ) density, we attempted to model that density in a variety of methods. First, we did not enforce any base-pairing in the region, resulting in an unstructured RNA chain modeled into the density. We named this model “unordered”. Second, to obtain models with more energetically favorable RNA fold in-line with literature (72), we enforced base-pairing between nucleotides in that region (Data S3). We used a published G-quadruplex model (PDB 1RAU) as a template (78). Two additional model ensembles were created, one forcing a base-pairing between U5 and G345 of hTR, and another between C7 and G345 of hTR (Data S3). For the full telomerase dimer map, a similar method was employed with first isolating the cryo-EM map around the RNA to be modeled (residues 1–40, 185–210, 219–250 and 320–355). Secondary structure was enforced, and template helices and quadruplex were used as above, but there were no restraints placed on initial positions. Additionally, both RNA chains were modeled separately. The lowest energy models of each RNA chain were selected and combined to create an ensemble of full dimer models (Data S1).

### Q-score calculations for the 5’ leader sequence of hTR

From all sets of model ensembles, the best 5’ GQ model was selected based on the highest Q-score (model number 6 with 15 Å restraints). Additionally, the unordered model for the same region was generated without enforcing base-pairing as described above. The best 5’ GQ model (ordered 5’GQ), the unordered model, and the 5’ hairpin (PDB 7V9A) (10) were compared to determine the most suitable conformation of the 5’ hTR leader sequence (residues 1–17) based on map-vervus-model Q-scores using the 3.9 Å H/ACA RNP dimer map. The backbone and overall Q-scores were calculated in UCSF Chimera and ChimeraX (77, 79, 80). In ChimeraX, a Q-score plugin (courtesy of Tristan Croll) was used to visualize the backbone Q-scores for individual residues (fig. S20) (79). Q-scores for unordered model, ordered 5’ GQ, and 5’ hairpin generated in Chimera are also included in Data S2.

### Map and model visualization

Maps were visualized in both Chimera and ChimeraX. Illustrations were prepared using Chimera, ChimeraX (77), Pymol (www.pymol.org) and Adobe Illustrator.

## Supplementary Material

Supp Figs

Supp Movie

## Data Availability

Cryo-EM maps are deposited with the Electron Microscopy Database under accession numbers: EMD-52976 for the full dimer, EMD-52983 for the H/ACA RNP protomer, EMD-52984 for the H/ACA RNP dimer, EMD-52978 for the catalytic core obtained by symmetry expansion, EMD-52979 for the catalytic core 1, EMD-52980 for the catalytic core 2, EMD-52981 for the catalytic core 1 using the common subset of particles, and EMD-52982 for the catalytic core 2 using the common subset of particles. PDB coordinates are deposited with the Protein Data Bank under accession number PDB ID 9QB2 for the H/ACA RNP protomer, 9QB3 for the H/ACA RNP dimer, 9QAX for the catalytic core obtained by symmetry expansion, 9QAY for the catalytic core 1, and 9QAZ for the catalytic core 2. For the full dimer, the corresponding fitted models and the RNA models built by DRRAFTER (not refined) are included as a Pymol session in Data S1. Q-scores of different models for the 5’ leader sequence of hTR are included in Data S2. The G-quadruplex model built by DRRAFTER is included in as a Pymol session in the Data S3. The raw nanopore sequence data of the DMS RNA structure probing data have been deposited in the European Nucleotide Archive (ENA) at EMBL-EBI under accession number PRJEB88555 (https://www.ebi.ac.uk/ena/browser/view/PRJEB88555). Materials are available from T.H.D.N. under a material transfer agreement with the MRC Laboratory of Molecular Biology. Correspondence should be addressed to T.H.D.N.

## One-Sentence Summary

Structure of human telomerase dimer provides insights into its composition and function in telomere maintenance.

## References

[1] Levy MZ, Allsopp RC, Futcher AB, Greider CW, Harley CB (1992). Telomere end-replication problem and cell aging. J Mol Biol.

[2] Wu RA, Upton HE, Vogan JM, Collins K (2017). Telomerase Mechanism of Telomere Synthesis. Annu Rev Biochem.

[3] Blackburn EH, Collins K, Gesteland RF, Atkins JF, Cech TR (2010). RNA Worlds.

[4] Kim NW (1994). Specific association of human telomerase activity with immortal cells and cancer. Science.

[5] Shay JW, Bacchetti S (1997). A survey of telomerase activity in human cancer. Eur J Cancer.

[6] Sarek G, Marzec P, Margalef P, Boulton SJ (2015). Molecular basis of telomere dysfunction in human genetic diseases. Nat Struct Mol Biol.

[7] Armanios M (2022). The Role of Telomeres in Human Disease. Annu Rev Genomics Hum Genet.

[8] Nguyen THD (2018). Cryo-EM structure of substrate-bound human telomerase holoenzyme. Nature.

[9] Ghanim GE (2021). Structure of human telomerase holoenzyme with bound telomeric DNA. Nature.

[10] Wan F (2021). Zipper head mechanism of telomere synthesis by human telomerase. Cell Res.

[11] Egan ED, Collins K (2012). Biogenesis of telomerase ribonucleoproteins. RNA.

[12] Venteicher AS (2009). A human telomerase holoenzyme protein required for Cajal body localization and telomere synthesis. Science.

[13] Tycowski KT, Shu MD, Kukoyi A, Steitz JA (2009). A conserved WD40 protein binds the Cajal body localization signal of scaRNP particles. Mol Cell.

[14] Schnapp G, Rodi H-P, Rettig WJ, Schnapp A, Damm K (1998). One-step affinity purification protocol for human telomerase. Nucleic Acids Res.

[15] Wenz C (2001). Human telomerase contains two cooperating telomerase RNA molecules. EMBO J.

[16] Cohen SB (2007). Protein composition of catalytically active human telomerase from immortal cells. Science.

[17] Sauerwald A (2013). Structure of active dimeric human telomerase. Nat Struct Mol Biol.

[18] Alves D (2008). Single-molecule analysis of human telomerase monomer. Nat Chem Biol.

[19] Wu RA, Dagdas YS, Yilmaz ST, Yildiz A, Collins K (2015). Single-molecule imaging of telomerase reverse transcriptase in human telomerase holoenzyme and minimal RNP complexes. eLife.

[20] Errington TM, Fu D, Wong JM, Collins K (2008). Disease-associated human telomerase RNA variants show loss of function for telomere synthesis without dominant-negative interference. Mol Cell Biol.

[21] Egan ED, Collins K (2010). Specificity and stoichiometry of subunit interactions in the human telomerase holoenzyme assembled in vio. Mol Cell Biol.

[22] Liu B (2022). Structure of active human telomerase with telomere shelterin protein TPP1. Nature.

[23] Sekne Z, Ghanim GE, Roon A-MMv, Nguyen THD (2022). Structural basis of human telomerase recruitment by TPP1-POT1. Science.

[24] Dixon AS (2016). NanoLuc Complementation Reporter Optimized for Accurate Measurement of Protein Interactions in Cells. ACS Chem Biol.

[25] Beattie TL, Zhou W, Robinson MO, Harrington L (2001). Functional multimerization of the human telomerase reverse transcriptase. Mol Cell Biol.

[26] Kappel K (2018). De novo computational RNA modeling into cryo-EM maps of large ribonucleoprotein complexes. Nature Methods.

[27] Ghanim GE, Sekne Z, Balch S, van Roon A-MM, Nguyen THD (2024). 2.7 Å cryo-EM structure of human telomerase H/ACA ribonucleoprotein. Nat Comms.

[28] Sayed ME (2019). Catalysis-dependent inactivation of human telomerase and its reactivation by intracellular telomerase-activating factors (iTAFs). J Biol Chem.

[29] Mitchell JR, Cheng J, Collins K (1999). A box H/ACA small nucleolar RNA-like domain at the human telomerase RNA 3’ end. Mol Cell Biol.

[30] Kiss T, Fayet-Lebaron E, Jády BE (2010). Box H/ACA small ribonucleoproteins. Mol Cell.

[31] Vogan JM (2016). Minimized human telomerase maintains telomeres and resolves endogenous roles of H/ACA proteins, TCAB1, and Cajal bodies. eLife.

[32] Egan ED, Collins K (2012). An enhanced H/ACA RNP assembly mechanism for human telomerase RNA. Mol Cell Biol.

[33] Trahan C, Martel C, Dragon F (2010). Effects of dyskeratosis congenita mutations in dyskerin, NHP2 and NOP10 on assembly of H/ACA pre-RNPs. Hum Mol Genet.

[34] Fu D, Collins K (2003). Distinct biogenesis pathways for human telomerase RNA and H/ACA small nucleolar RNAs. Mol Cell.

[35] Chong PA, Vernon RM, Forman-Kay JD (2018). RGG/RG Motif Regions in RNA Binding and Phase Separation. J Mol Biol.

[36] Gros J, Guédin A, Mergny JL, Lacroix L (2008). G-Quadruplex Formation Interferes with P1 Helix Formation in the RNA Component of Telomerase hTERC. Chem Bio Chem.

[37] Li X (2007). Structure, Interactions and Effects on Activity of the 5’-terminal Region of Human telomerase RNA. J Biochem.

[38] Sexton AN, Collins K (2011). The 5’ guanosine tracts of human telomerase RNA are recognized by the G-quadruplex binding domain of the RNA helicase DHX36 and function to increase RNA accumulation. Mol Cell Biol.

[39] Lattmann S, Stadler MB, Vaughn JP, Akman SA, Nagamine Y (2011). The DEAH-box RNA helicase RHAU binds an intramolecular RNA G-quadruplex in TERC and associates with telomerase holoenzyme. Nucleic Acids Res.

[40] Chen JL, Blasco MA, Greider CW (2000). Secondary structure of vertebrate telomerase RNA. Cell.

[41] Booy EP (2012). The RNA helicase RHAU (DHX36) unwinds a G4-quadruplex in human telomerase RNA and promotes the formation of the P1 helix template boundary. Nucleic Acids Res.

[42] Lu Z (2024). Identification of G-quadruplex-interacting proteins in living cells using an artificial G4-targeting biotin ligase. Nucleic Acids Research.

[43] Wapling J, Moore KL, Sonza S, Mak J, Tachedjian G (2005). Mutations that abrogate human immunodeficiency virus type 1 reverse transcriptase dimerization affect maturation of the reverse transcriptase heterodimer. J Virol.

[44] Restle T, Müller B, Goody RS (1990). Dimerization of human immunodeficiency virus type 1 reverse transcriptase. A target for chemotherapeutic intervention. Journal of Biological Chemistry.

[45] Fletcher RS (1996). Single-Step Purification of Recombinant Wild-Type and Mutant HIV-1 Reverse Transcriptase. Protein Expression and Purification.

[46] Tseng C-K, Wang H-F, Schroeder MR, Baumann P (2018). The H/ACA complex disrupts triplex in hTR precursor to permit processing by RRP6 and PARN. Nature Commun.

[47] Zyner KG (2019). Genetic interactions of G-quadruplexes in humans. Elife.

[48] Hendriks IA (2014). Uncovering global SUMOylation signaling networks in a site-specific manner. Nature Structural & Molecular Biology.

[49] Whitehead SE (2002). Determinants of the interaction of the spinal muscular atrophy disease protein SMN with the dimethylarginine-modified box H/ACA small nucleolar ribonucleoprotein GAR1. J Biol Chem.

[50] Schmidt JC, Zaug AJ, Cech TR (2016). Live Cell Imaging Reveals the Dynamics of Telomerase Recruitment to Telomeres. Cell.

[51] Jiang J (2013). The architecture of *Tetrahymena* telomerase holoenzyme. Nature.

[52] Bajon E, Laterreur N, Wellinger RJ (2015). A single templating RNA in yeast telomerase. Cell Rep.

[53] Podlevsky JD, Bley CJ, Omana RV, Qi X, Chen JJ (2008). The telomerase database. Nucleic Acids Res.

[54] Kimanius D, Dong L, Sharov G, Nakane T, Scheres SHW (2021). New tools for automated cryo-EM single-particle analysis in RELION-4.0. Biochem J.

[55] Kimanius D (2024). Data-driven regularization lowers the size barrier of cryo-EM structure determination. Nature Methods.

[56] Rohou A, Grigorieff N (2015). CTFFIND4: Fast and accurate defocus estimation from electron micrographs. J Struct Biol.

[57] Zivanov J (2018). New tools for automated high-resolution cryo-EM structure determination in RELION-3. eLife.

[58] Zubradt M (2017). DMS-MaPseq for genome-wide or targeted RNA structure probing in vivo. Nature Methods.

[59] Mitchell D, Cotter J, Saleem I, Mustoe AM (2023). Mutation signature filtering enables high-fidelity RNA structure probing at all four nucleobases with DMS. Nucleic Acids Research.

[60] Li H (2018). Minimap2: pairwise alignment for nucleotide sequences. Bioinformatics.

[61] Forino NM (2025). Telomerase RNA structural heterogeneity in living human cells detected by DMS-MaPseq. Nature Communications.

[62] Punjani A, Rubinstein JL, Fleet DJ, Brubaker MA (2017). cryoSPARC: algorithms for rapid unsupervised cryo-EM structure determination. Nat Methods.

[63] Bepler T (2019). Positive-unlabeled convolutional neural networks for particle picking in cryo-electron micrographs. Nat Methods.

[64] Punjani A, Zhang H, Fleet DJ (2020). Non-uniform refinement: adaptive regularization improves single-particle cryo-EM reconstruction. Nat Methods.

[65] Asarnow D, Palovcak E, Cheng Y (2019).

[66] Punjani A, Fleet DJ (2021). 3D variability analysis: Resolving continuous flexibility and discrete heterogeneity from single particle cryo-EM. J Struct Biol.

[67] Zivanov J, Nakane T, Scheres SHW (2020). Estimation of high-order aberrations and anisotropic magnification from cryo-EM data sets in RELION-3.1. IUCrJ.

[68] Murshudov GN (2011). REFMAC5 for the refinement of macromolecular crystal structures. Acta Crystallogr D Biol Crystallogr.

[69] Brown A (2015). Tools for macromolecular model building and refinement into electron cryo-microscopy reconstructions. Acta Crystallogr D Biol Crystallogr.

[70] Casañal A, Lohkamp B, Emsley P (2020). Current developments in Coot for macromolecular model building of Electron Cryo-microscopy and Crystallographic Data. Protein Sci.

[71] Chou F-C, Echols N, Terwilliger TC, Das R (2016). RNA Structure Refinement Using the ERRASER-Phenix Pipeline. Methods Mol Biol.

[72] Yamashita K, Palmer CM, Burnley T, Murshudov GN (2021). Cryo-EM single-particle structure refinement and map calculation using Servalcat. Acta Crystallogr D Struct Biol.

[73] Nicholls RA, Fischer M, McNicholas S, Murshudov GN (2014). Conformation-independent structural comparison of macromolecules with ProSMART. Acta Crystallogr D Biol Crystallogr.

[74] Liebschner D (2019). Macromolecular structure determination using X-rays, neutrons and electrons: recent developments in Phenix. Acta Crystallogr D Biol Crystallogr.

[75] Williams CJ (2018). MolProbity: More and better reference data for improved all-atom structure validation. Protein Sci.

[76] Martadinata H, Phan AT (2014). Formation of a stacked dimeric G-quadruplex containing bulges by the 5’-terminal region of human telomerase RNA (hTERC). Biochemistry.

[77] Goddard TD (2018). UCSF ChimeraX: Meeting modern challenges in visualization and analysis. Protein Sci.

[78] Cheong C, Moore PB (1992). Solution structure of an unusually stable RNA tetraplex containing G- and U-quartet structures. Biochemistry.

[79] Pintilie G (2020). Measurement of atom resolvability in cryo-EM maps with Q-scores. Nat Methods.

[80] Pettersen EF (2004). UCSF Chimera--a visualization system for exploratory research and analysis. J Comput Chem.

