# Supplementary material for: Cryo-EM structure of human telomerase dimer reveals H/ACA RNP-mediated dimerization
Source: Science. Author manuscript; Available in PMC 2025 Sep 18. (PMC7618144; doi:10.1126/science.adr5817)
Supplement: Supp Figs [file EMS205719-supplement-Supp_Figs.pdf]

Science
AAAS

Supplementary Materials for

Cryo-EM structure of human telomerase dimer reveals H/ACA RNP-mediated dimerization

Sebastian Balch†, Zala Sekne†, Elsa Franco-Echevarría†, Patryk Ludzia, Rachael C Kretsch, Wenqing Sun, Haopeng Yu, George E Ghanim, Sigurdur Thorkelsson, Yiliang Ding, Rhiju Das, Thi Hoang Duong Nguyen*

† These authors contributed equally to this work.
*Corresponding author: knguyen@mrc-lmb.cam.ac.uk

The PDF file includes:

Figs. S1 to S20
Tables S1 to S3
Caption for Movie S1
Captions for Data S1 to S3
References (*81*-*83*)

Other Supplementary Materials for this manuscript include the following:

Movie S1
Data S1 to S3

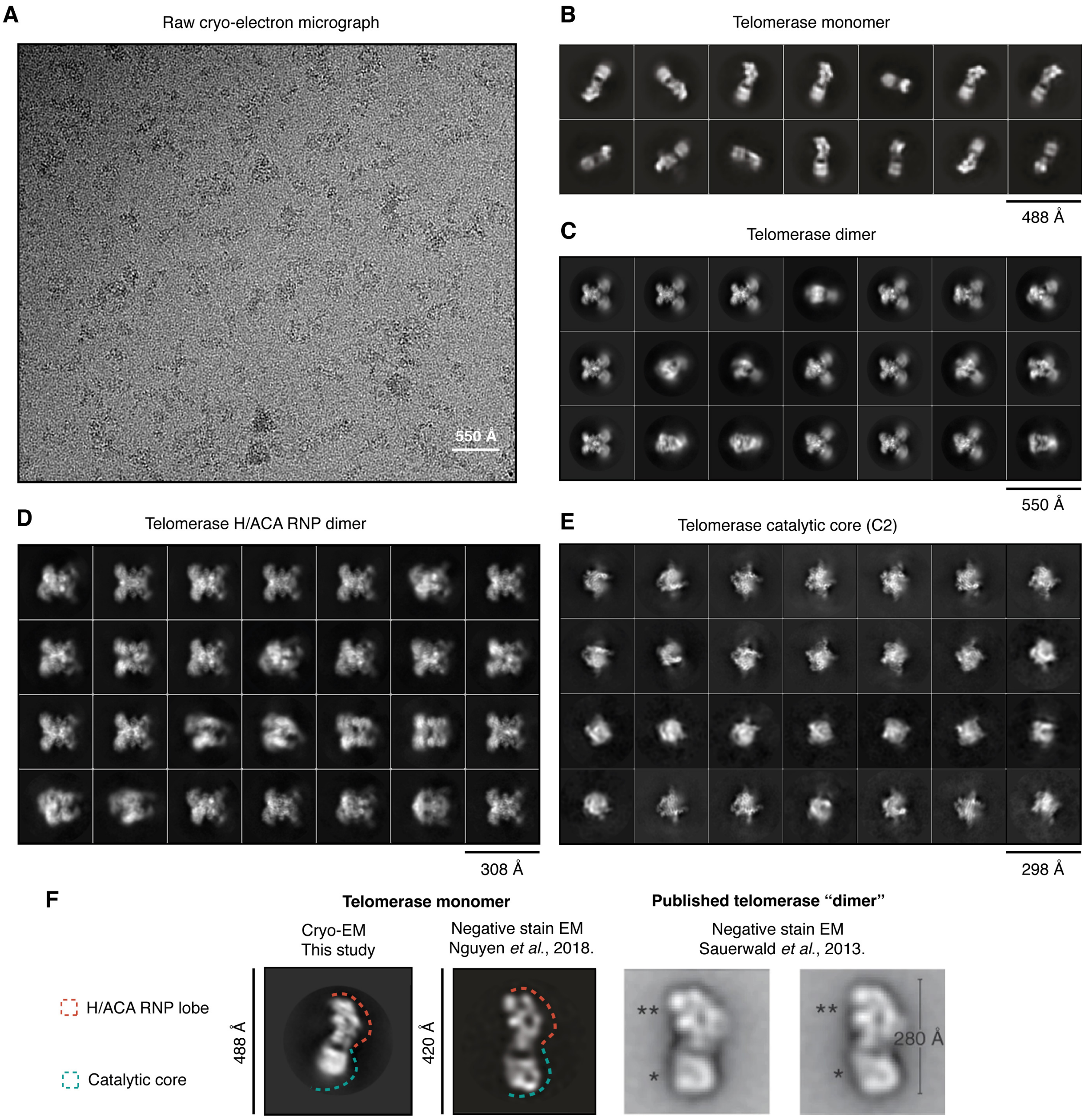

Fig. S1. Representative cryo-EM data.

(A) Representative cryo-EM micrograph. **(B to E)** Cryo-EM 2D class averages of the full monomeric telomerase (B), full dimeric telomerase (C), telomerase H/ACA RNP dimer (D), and catalytic core (E) from the dimeric telomerase, respectively. **(F)** Comparison of the cryo-EM and negative stain EM 2D class averages of the human telomerase monomer from this study and a previous study *(8)* with those of the published negative stain EM structure of the proposed dimer of TERT and hTR *(17)*.

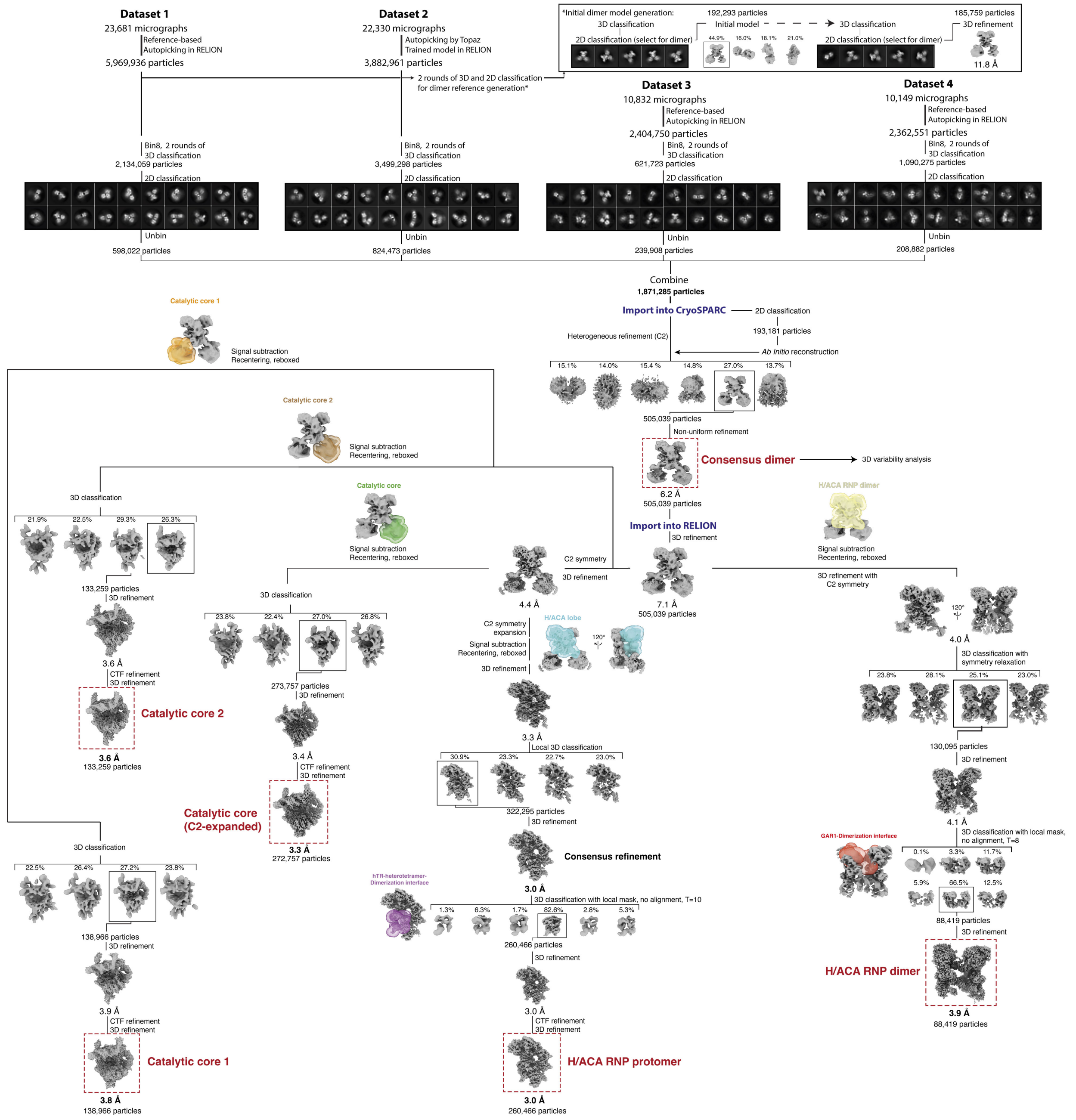

Fig. S2. Cryo-EM data processing.

Image processing strategies to obtain the maps presented in this study. Red boxes and labels indicate maps discussed in the main text.

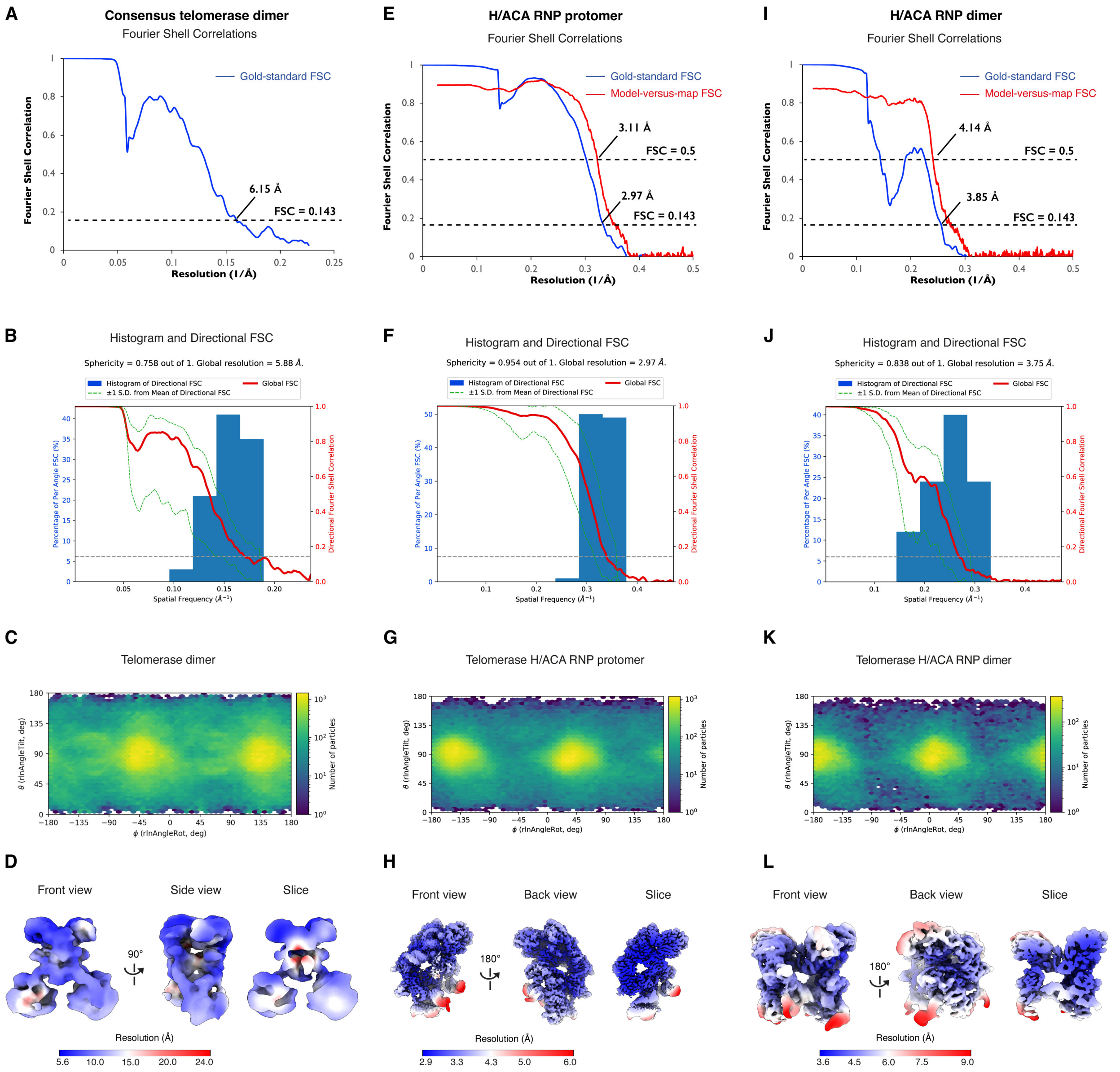

Fig. S3. Overall and local resolution estimation for the full telomerase dimer, the H/ACA RNP protomer and the H/ACA RNP dimer.

(A, E, I) Model-versus-map (red) and gold-standard (blue) FSC plots for the full telomerase dimer ((A) - only gold-standard FSC plot), telomerase H/ACA RNP protomer (E) and telomerase H/ACA RNP dimer (I). The resolution was estimated at FSC = 0.5 (model-versus-map) and FSC = 0.143 (gold-standard). **(B, F, J)** Directional FSC plots and sphericity values are presented for the full telomerase dimer (B), telomerase H/ACA RNP protomer (F) and telomerase H/ACA RNP dimer (J) (*81*). Directional FSC plots were generated using a 3D-FSC server (https://3dfsc.salk.edu). **(C, G, K)** 2D histograms depict the Euler angles of particles used for reconstructions of the full telomerase dimer (C), telomerase H/ACA RNP protomer (G) and telomerase H/ACA RNP dimer (K). Histograms were plotted using a Python script (https://githubhelp.com/Guillawme/angdist). **(D, H, L)** Local resolution of the full telomerase dimer (D), telomerase H/ACA RNP protomer (H)

and telomerase H/ACA RNP dimer (L) maps. RELION 5.0 was used to generate local resolution estimation.

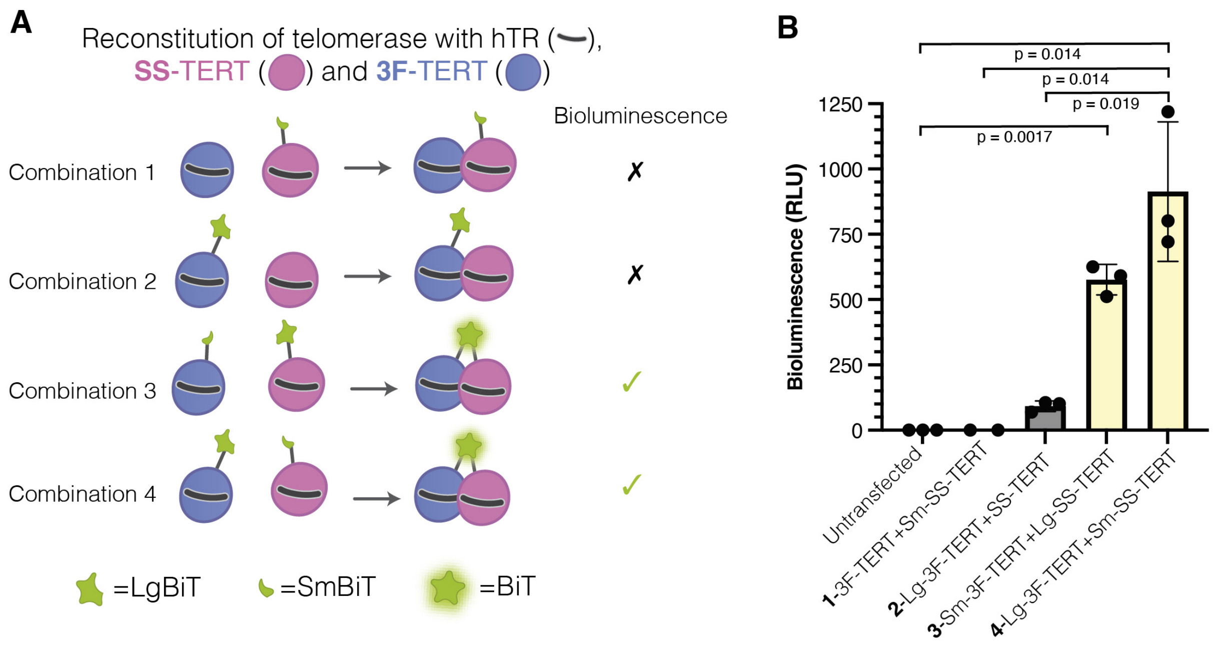

Fig. S4. Split nano-luciferase assays.

(A) Schematic of the split nano-luciferase assays. Four combinations of TERT constructs were co-transfected with hTR into HEK293T cells as shown. Bioluminescence is obtained when the LgBiT and SmBiT fragments of nano-luciferase are in close proximity. **(B)** Analysis of the bioluminescence from the nano-luciferase assays for the different combinations of TERT constructs co-transfected with hTR as shown in (A). Experiments were done in triplicates. Error bars represent the standard error of the mean (SEM), and significant p values are also reported.

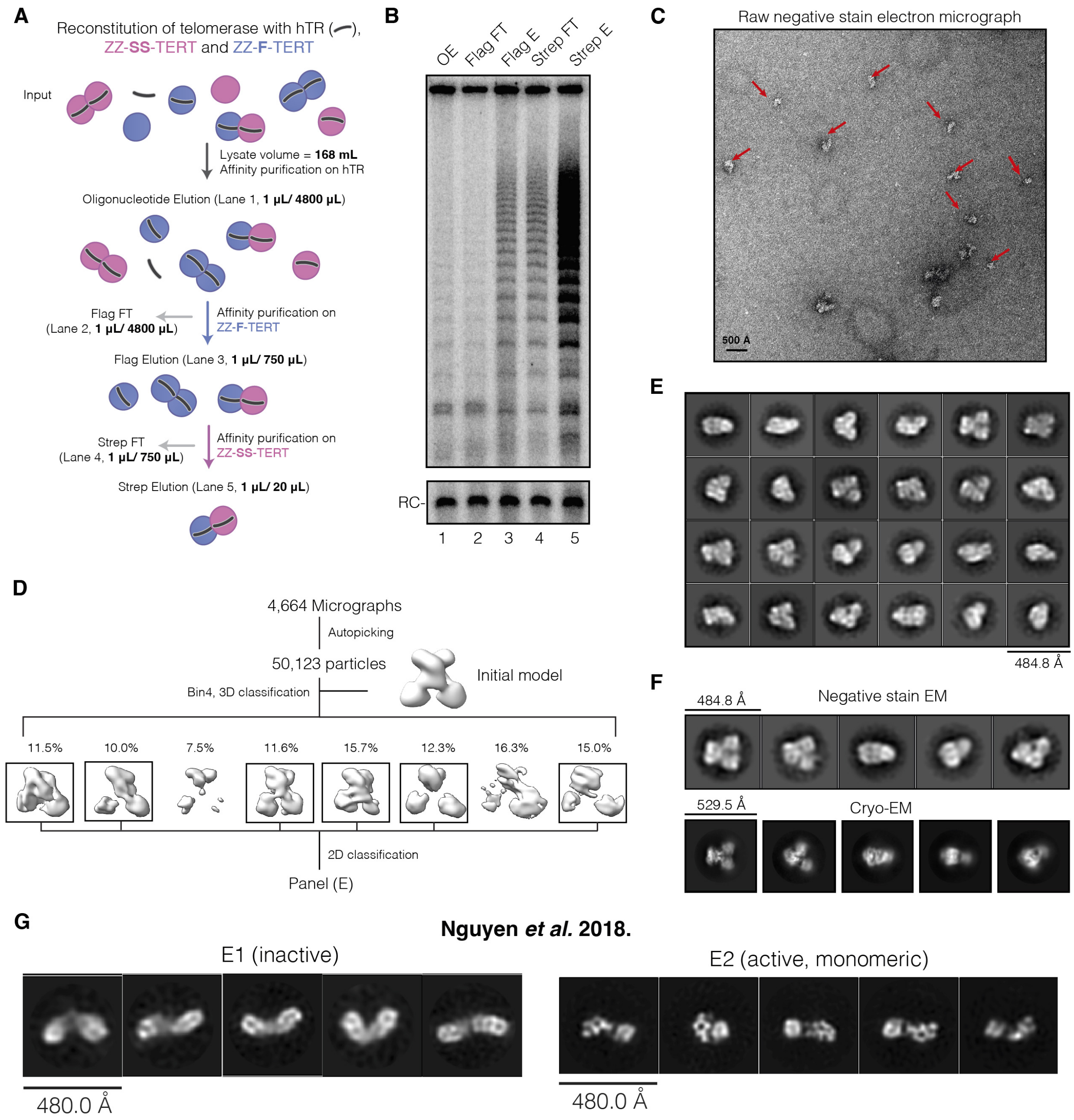

Fig. S5. Biochemical reconstitution and characterization of the human telomerase holoenzyme dimer.

(A) Schematic of the biochemical purification to isolate dual-tagged dimeric telomerase. Samples were collected for telomerase activity assays shown in (B). 1 µL of sample at different steps of the purification was collected for telomerase activity assays. The total volume of telomerase samples at these different steps is also indicated. **(B)** Telomerase activity assays of the samples collected in (A). OE, oligonucleotide elution; Flag FT, Flag flow-through; Flag E, Flag elution; Strep FT, Strep-Tactin flow-through; Strep E, Strep-Tactin elution. **(C)** Representative negative stain EM image of telomerase with both Flag and Strep tags resulting from the purification strategy shown in (A). **(D)** Negative stain EM data processing strategy for the purified dual-tagged telomerase sample shown

in (C). **(E)** 2D class averages of telomerase complexes resulting from Strep E shown in (A). **(F)** Comparison between representative negative stain 2D class averages of the purified dual-tagged telomerase particles and the cryo-EM 2D class averages of the telomerase dimer. **(G)** Representative negative stain EM 2D class averages of two fractions of telomerase with different activities from a previous study (*8*). These fractions resulted from biotin elution of telomerase from magnetic Strep-Tactin resin. The first elution fraction (E1) is inactive and has a different structural morphology to the second elution fraction (E2), which is active and has monomeric TERT/hTR composition and additional holoenzyme factors.

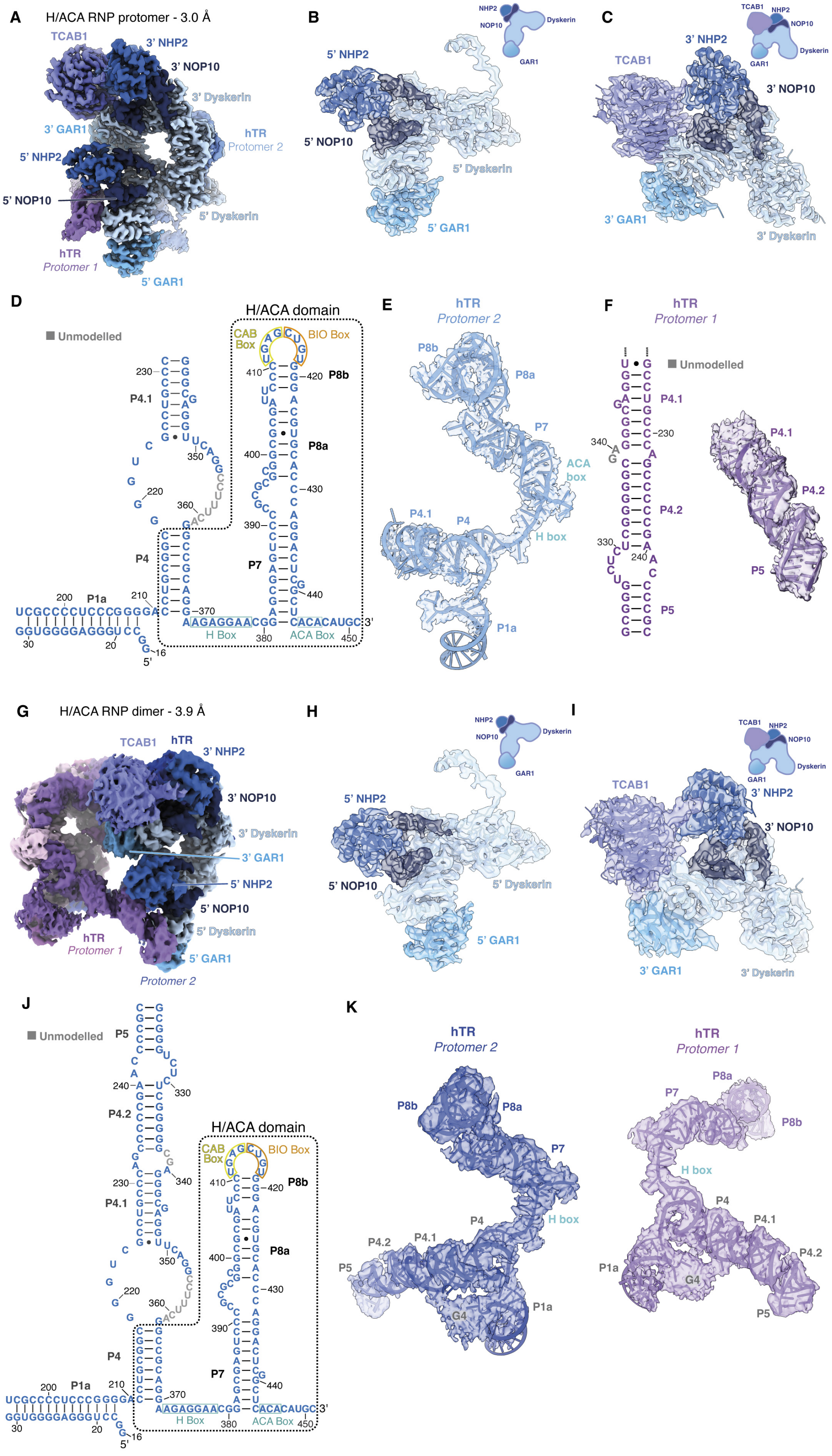
A
H/ACA RNP protomer - 3.0 Å
TCAB1
3' NHP2
3' NOP10
3' Dyskerin
3' GAR1
hTR
Protomer 2
5' NHP2
5' NOP10
5' Dyskerin
hTR
Protomer 1
5' GAR1
B
NHP2
NOP10
Dyskerin
GAR1
5' NHP2
5' NOP10
5' Dyskerin
5' GAR1
C
TCAB1
NHP2
NOP10
Dyskerin
GAR1
TCAB1
3' NHP2
3' NOP10
3' GAR1
3' Dyskerin
D
H/ACA domain
Unmodelled
CAB Box
BIO Box
P8b
P8a
P7
P4.1
P4
P1a
UCGCCCCUCCCGGGGA
GGUGGGGAGGGUCC
H Box
ACA Box
AGAGGAA
CACACAUGC
3'
5'
E
hTR
Protomer 2
P8b
P8a
P7
ACA box
P4.1
P4
H box
P1a
F
hTR
Protomer 1
Unmodelled
P4.1
P4.2
P5
G
H/ACA RNP dimer - 3.9 Å
TCAB1
hTR
3' NHP2
3' NOP10
3' Dyskerin
3' GAR1
5' NHP2
5' NOP10
5' Dyskerin
5' GAR1
hTR
Protomer 1
Protomer 2
H
NHP2
NOP10
Dyskerin
GAR1
5' NHP2
5' NOP10
5' Dyskerin
5' GAR1
I
TCAB1
NHP2
NOP10
Dyskerin
GAR1
TCAB1
3' NHP2
3' NOP10
3' GAR1
3' Dyskerin
J
Unmodelled
P5
P4.2
P4.1
H/ACA domain
CAB Box
BIO Box
P8b
P8a
P7
P4
P1a
UCGCCCCUCCCGGGGA
GGUGGGGAGGGUCC
H Box
ACA Box
AGAGGAA
CACACAUGC
3'
5'
K
hTR
Protomer 2
P8b
P8a
P7
H box
P4
P4.1
P4.2
P5
G4
P1a
hTR
Protomer 1
P8a
P7
P8b
H box
P4
P4.1
P1a
G4
P4.2
P5

Fig. S6. Representative cryo-EM density of the 3.0 Å H/ACA RNP protomer and the 3.9 Å H/ACA RNP dimer map.

(A) 3.0 Å cryo-EM density of the H/ACA RNP lobe of a single protomer in the telomerase dimer. **(B)** Model fitted into the map of the 5' H/ACA heterotetramer of the H/ACA RNP protomer. **(C)** Model fitted into the map of the 3' H/ACA heterotetramer and TCAB1 of the H/ACA RNP protomer. **(D)** Schematic of hTR modelled in the H/ACA RNP protomer. The H/ACA domain of hTR is labelled in with a dashed box. **(E)** Model of hTR from protomer 2 fitted into the cryo-EM density of the H/ACA RNP protomer. **(F)** Schematic and model-map fit of the P4/P5 linker of hTR from the protomer 1 that interacts with H/ACA proteins of protomer 2. **(G)** 3.9 Å cryo-EM density map of the H/ACA RNP dimer. Only protomer 2 is labelled for simplicity. **(H)** Model of the 5' H/ACA heterotetramer of protomer 2 fitted into the 3.9 Å H/ACA RNP dimer map. **(I)** Model of the 3' H/ACA heterotetramer and TCAB1 of protomer 2 fitted into the 3.9 Å H/ACA RNP dimer map. **(J)** Schematic of hTR modelled into the H/ACA RNP dimer. **(K)** Models of hTR from both protomers of the H/ACA RNP dimer fitted into the 3.9 Å H/ACA RNP dimer map. G4, G quadruplex.

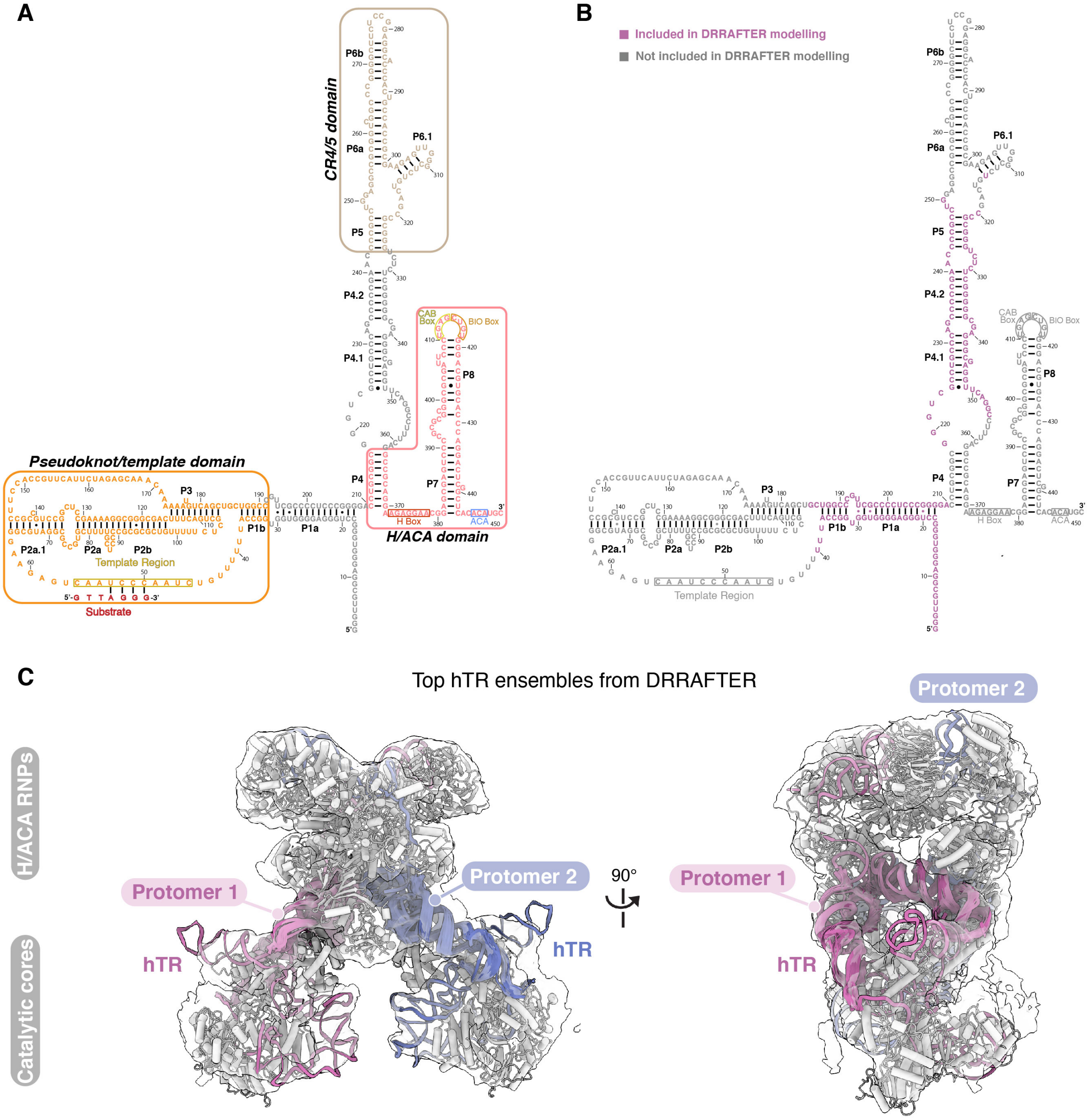

Fig. S7. DRRAFTER modeling of telomerase RNA in the full telomerase dimer.
(A) Sequence and secondary structure of hTR. The pseudoknot/template, CR4/5 and H/ACA domains are colored as indicated. **(B)** Sequence and secondary structure of hTR with regions included in DRRAFTER modeling colored in magenta. **(C)** The top DRRAFTER models of hTR in the full telomerase dimer using the 6.2 Å resolution map. DRRAFTER was performed on hTR of both protomers. Also see Data S1.

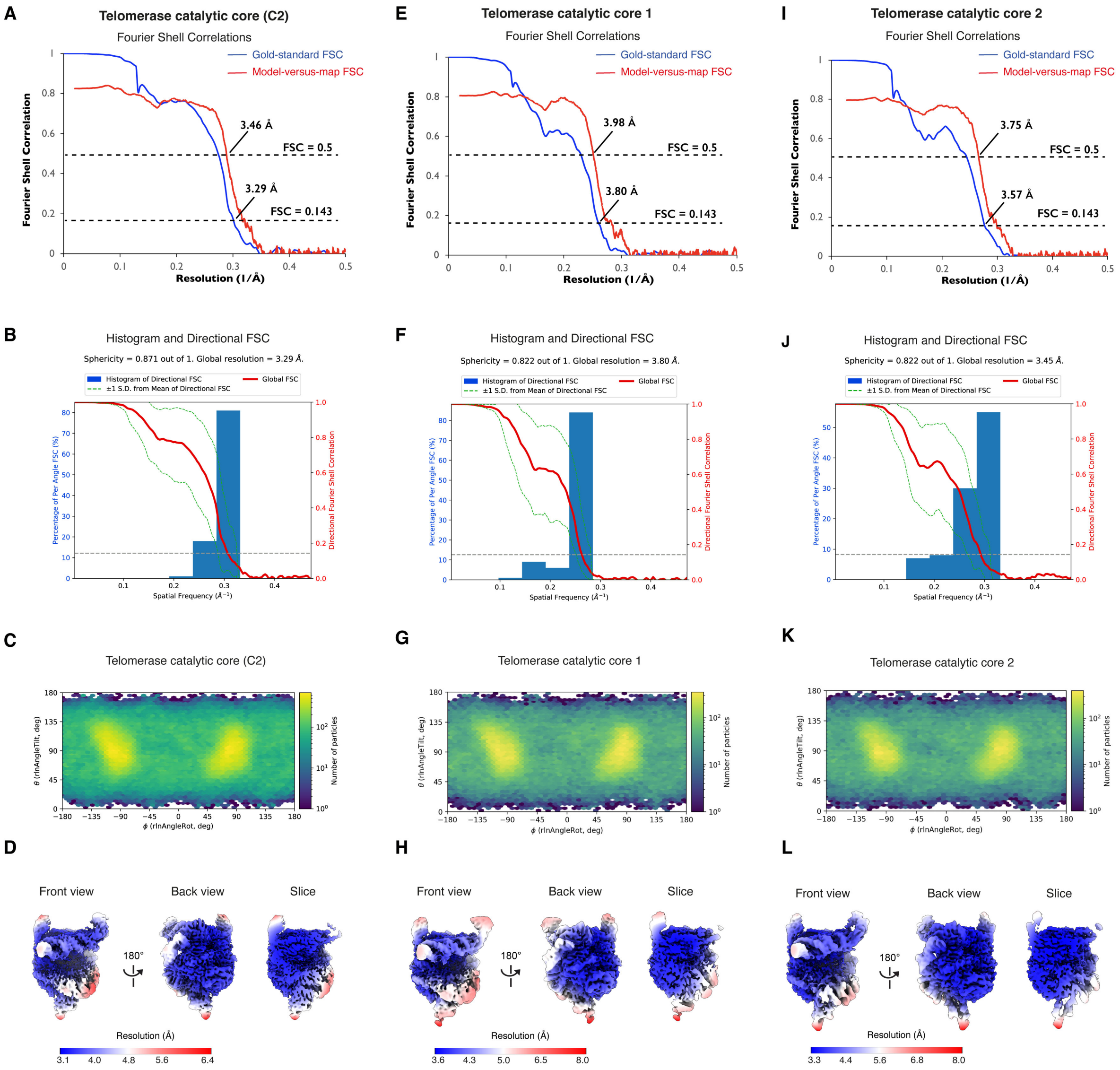

Fig. S8. Overall and local resolution estimation for the telomerase catalytic cores.
(A, E, I) Model-versus-map (red) and gold-standard (blue) FSC plots for the telomerase catalytic core (from the C2 symmetry expanded particle stack (C2)) (A), telomerase catalytic core 1 (E) and telomerase catalytic core 2 (I) for the full telomerase dimer. The resolution was estimated at FSC = 0.5 (model-versus-map) and FSC = 0.143 (gold-standard). **(B, F, J)** Directional FSC plots and sphericity values are presented for the telomerase catalytic core (C2) (B), telomerase catalytic core 1 (F) and telomerase catalytic core 2 (J) (*81*). Directional FSC plots were generated using a 3D-FSC server (https://3dfsc.salk.edu). **(C, G, K)** 2D histograms depict the Euler angles of particles used for reconstructions of the telomerase catalytic core (C2) (C), telomerase catalytic core 1 (G) and telomerase catalytic core 2 (K). Histograms were plotted using a Python script (https://githubhelp.com/Guillawme/angdist). **(D, H, L)** Local resolution of the telomerase catalytic core (C2) (D), telomerase catalytic core 1 (H) and telomerase catalytic core 2 (L) maps. RELION 5.0 was used to generate local resolution estimation.

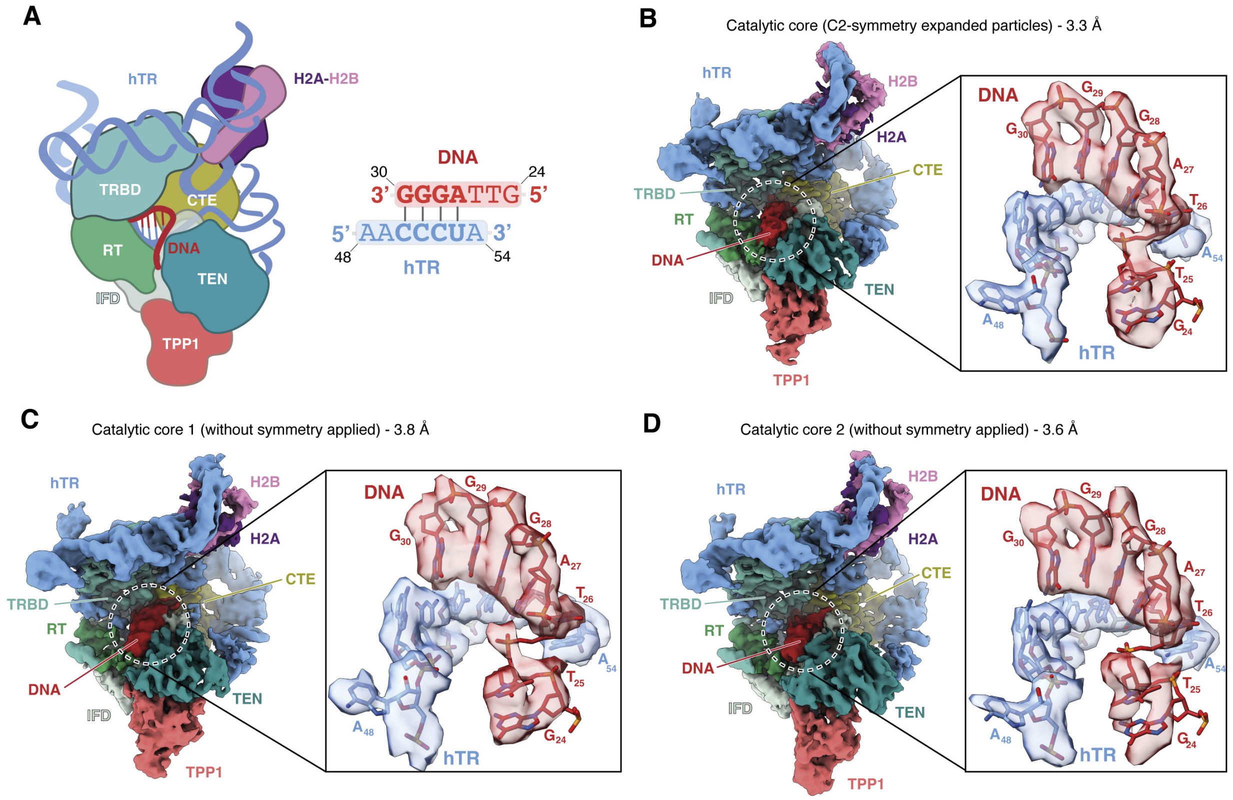

Fig. S9. Representative cryo-EM densities of the telomerase catalytic cores.
(A) Schematic of the human telomerase catalytic core with the shelterin subunit TPP1. A schematic of the RNA-DNA duplex in the active site is also shown. Human TERT subunit is divided into domains by colors. TEN, telomerase essential N-terminal; TRBD, telomerase RNA binding domain; RT, reverse transcriptase; IFD, insertion in the fingers domain; CTE, C-terminal extension; hTR, human telomerase RNA. **(B)** Cryo-EM density of the telomerase catalytic core obtained from the C2 symmetry expanded particle stack at 3.3 Å resolution. Subunits are colored and labelled as in (A). **(C)** Cryo-EM density of the catalytic core 1 of the human telomerase dimer at 3.8 Å resolution. **(D)** Cryo-EM density of the catalytic core 2 of the human telomerase dimer at 3.6 Å resolution. Insets in (B) to (D) show close-up views of the RNA-DNA duplex with the model fitted to the corresponding semi-transparent cryo-EM map.

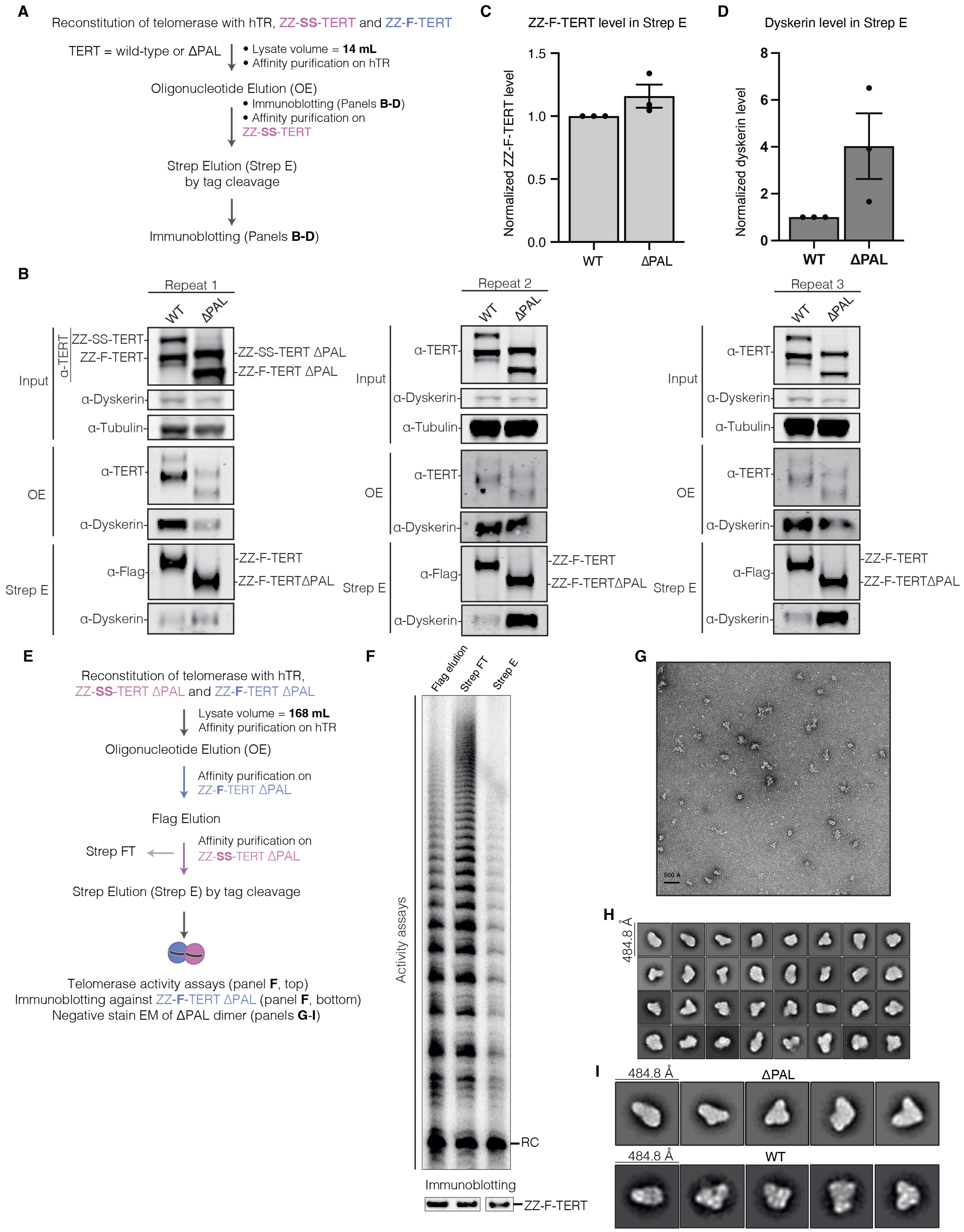

Fig. S10. Deleting the PAL of TERT does not affect dimer formation.

(**A**) Schematic of the two-step purification strategy to examine the ability of the TERT ΔPAL mutant to form the telomerase dimer. A 1:1 mixture of ZZ-SS-TERT and ZZ-F-TERT was co-

transfected with hTR, followed by O-purification and Strep-Tactin pulldown. The Flag signal in Strep E was used as a readout for dimer formation. The same experiment was also performed for wild-type (WT) TERT for comparison. **(B)** Triplicates of immunoblotting experiments of the input lysates (input), OE and Strep E from the purification of WT and ΔPAL TERT as shown in (A). **(C)** Bar graph showing the levels of ZZ-F-TERT in the Strep E fractions of the WT and ΔPAL purification. The signal of ZZ-F-TERT in the ΔPAL mutant was normalized against the WT signal. Error bars represent the standard error of the mean (SEM) (n = 3). **(D)** Bar graph showing the levels of dyskerin in the Strep E fractions for the WT and ΔPAL purification. The signal of dyskerin in the ΔPAL mutant was normalized against the WT signal. Error bars represent the standard error of the mean (SEM) (n = 3). **(E)** Schematic of the three-step purification strategy to isolate the ΔPAL mutant dimer for activity assays, immunoblotting and negative stain EM. **(F)** Telomerase activity assay (top panel) and immunoblotting (bottom panel) of samples from the Flag elution, the Strep flow-through (Strep FT) and the Strep E resulting from the purification of the ΔPAL mutant dimer as shown in (E). RC, recovery control. **(G)** Representative negative stain EM image of the purified ΔPAL mutant dimer. **(H)** Negative stain 2D class averages of the ΔPAL mutant dimer purified as shown in (E). **(I)** Comparison of representative negative stain 2D class averages of the purified ΔPAL mutant dimer (top) and the WT dimer (bottom).

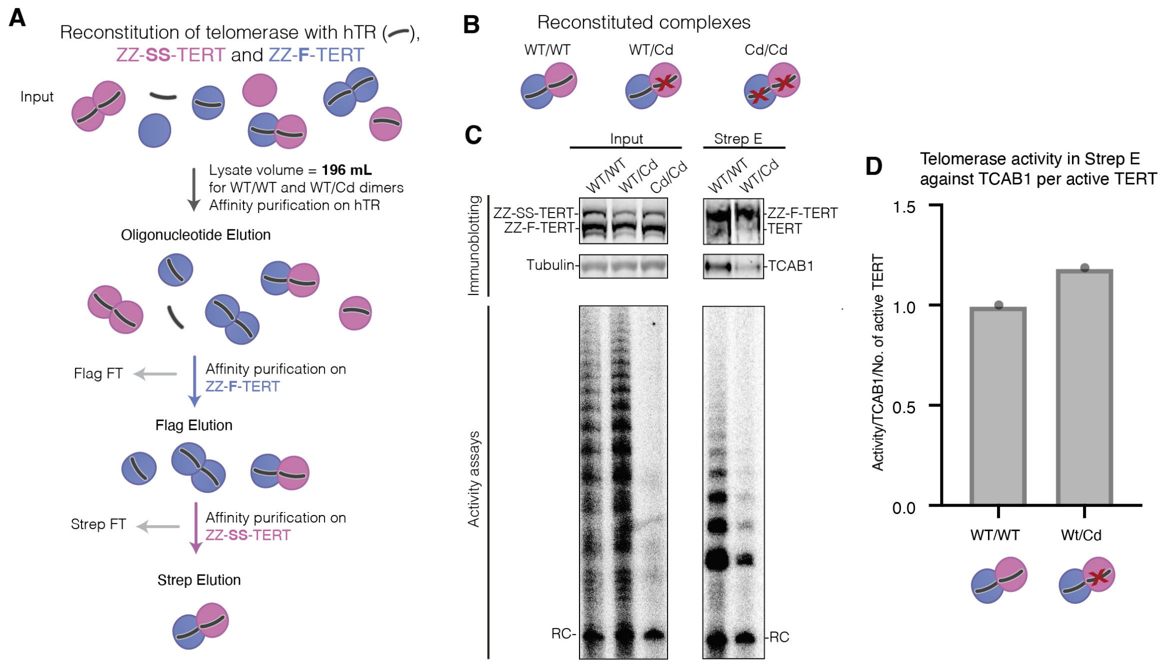

Fig. S11. Functional independence of the two catalytic cores within the telomerase dimer. **(A)** Schematic of the three-step purification strategy to isolate the dual-tagged telomerase with different TERT constructs. **(B)** Schematic of the three reconstituted telomerase complexes: one with both WT TERT (WT/WT), one with one WT TERT and one catalytically dead (Cd) TERT (WT/Cd), and one with both Cd TERT (Cd/Cd). We used the strategy shown in (A) to purify the WT/WT and WT/Cd dimers. The Cd/Cd reconstitution was only used as a negative control and not purified. **(C)** Immunoblotting (top panels) and telomerase activity assay (bottom panels) of the input lysates for the WT/WT, WT/Cd and Cd/Cd telomerase reconstitutions and the WT/WT and WT/Cd dimers purified as shown in (A). RC, recovery control. **(D)** Bar graph showing quantification of telomerase activity of the purified WT/WT and WT/Cd dimers. Telomerase activity was first normalized against the RC and then against TCAB1 per active TERT molecule (2 for WT/WT and 1 for WT/Cd). The normalized activity of the WT/Cd dimer is then normalized against the WT/WT dimer. Untagged TERT and ZZ-F-TERT should be present at an equal molar ratio using the three-step purification strategy shown in (A). However, the Proteintech TERT antibody gave weaker signals for the untagged TERT. TERT and TCAB1 are present at a 1:1 stoichiometry in telomerase. Thus, we used TCAB1 signal instead of TERT for quantification.

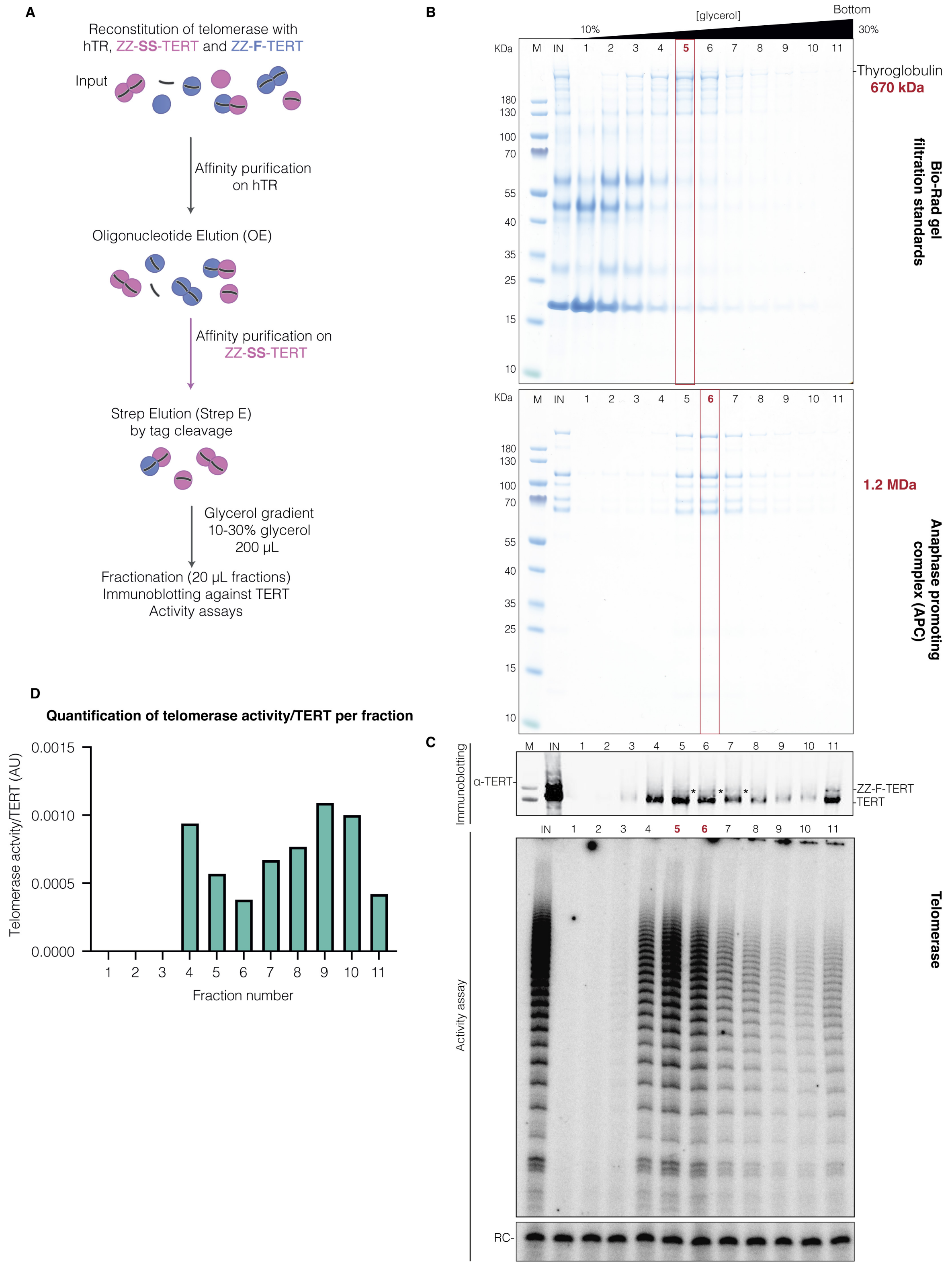

Fig. S12. Glycerol gradient centrifugation suggests that dimer formation did not enhance telomerase activity.

(A) Schematic of the biochemical strategy to separate the monomeric and dimeric telomerase complexes by glycerol gradient. Telomerase was reconstituted using ZZ-SS-TERT, ZZ-F-TERT and hTR, followed by O-purification and Strep-Tactin pulldown. The resulting Strep E sample would contain a mixture of monomeric (major) and dimeric (minor) telomerase complexes, which were separated by a 10-30% glycerol gradient. **(B)** Coomassie-stained SDS-PAGE of the input (IN) and fractions from 10-30% glycerol gradients of Bio-Rad gel filtration standards (top) and the anaphase promoting complex (APC) (bottom) as size markers. Thyroglobulin (670 KDa), with a size similar to the telomerase monomer, peaks at fraction 5. The APC complex (1.2 MDa), with a size similar to the telomerase dimer, peaks at fraction 6. The molecular weight markers are labelled as M. **(C)** Immunoblotting (top) and telomerase activity assay (bottom) of the input and fractions from a 10-30% glycerol gradient for Strep E sample from the two-step purification as shown in (A). Based on the size markers shown in (B), fractions 5 and 6 correspond to where the telomerase monomer and dimer peak, respectively. The signal of the ZZ-F-TERT in the fractions is an indicator of the dimeric complexes. Due to the low abundance of the dimer, these ZZ-F-TERT signal is weak and thus marked with asterisks on the right side of the bands. **(D)** Bar graph showing the quantification of telomerase activity per TERT across the fractions from the glycerol gradient of Strep E. Due to the overlap of the monomer and dimer peaks, we quantified the telomerase activity against TERT signals. If there was cooperativity between the two catalytic cores, we would observe more activity per TERT in the dimer peak fraction. However, we did not observe this trend, suggesting that dimer formation does not lead to increase in activity. These experiments could not be performed in triplicates due to the discontinuation of a commercial TERT antibody used for the immunoblotting experiment.

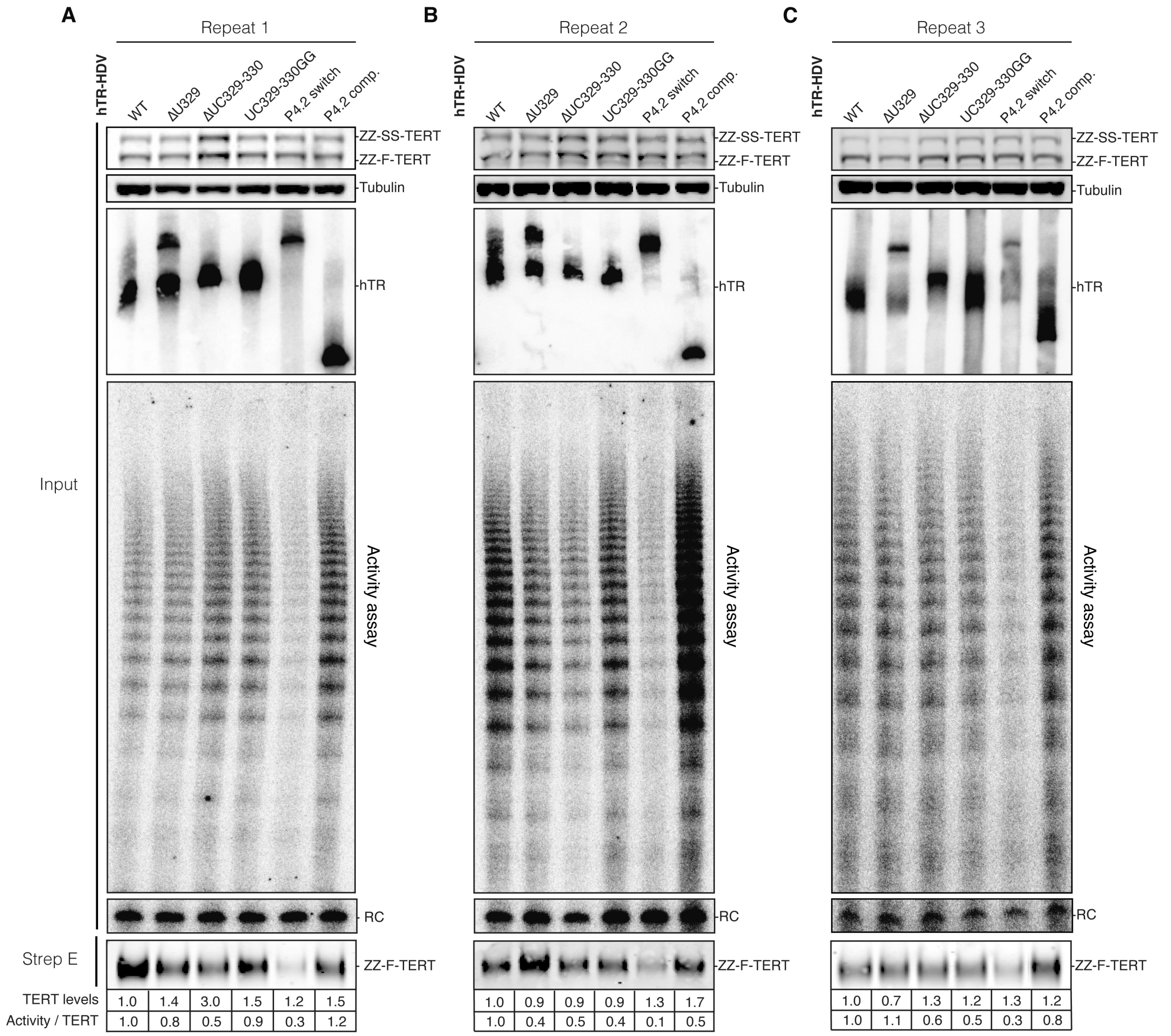

Fig. S13. Mutations in the P4.2/P5 linker region of hTR that disrupt dimer formation reduce telomerase activity.

(A to C) Triplicates of experiments characterizing the effects of disrupting the P4/5 linker regions of hTR. In this experiment, WT and mutant hTR constructs were reconstituted with ZZ-SS-TERT and ZZ-F-TERT. Telomerase was purified as shown in Fig. 1D; and the signal of ZZ-F-TERT in the Strep E was probed by immunoblotting as a readout for dimer formation. Also see Fig. 4B for quantification.

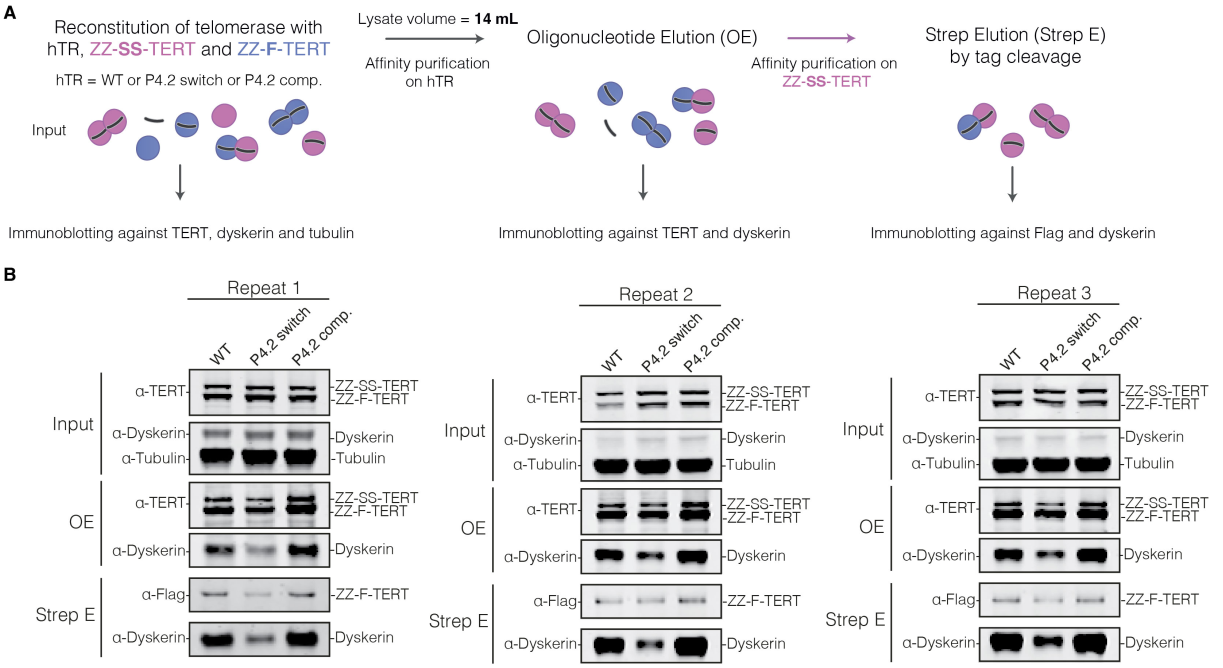

Fig. S14. Disrupting the P4.2 stem of hTR severely reduces binding of dyskerin.
(A) Schematic of the biochemical strategy to dissect the effects of disrupting the P4.2 stem of hTR for the data shown in Fig. 4D. Each of hTR construct, either WT or P4.2 switch or P4.2 comp. mutant, was reconstituted with a mixture of ZZ-SS-TERT and ZZ-F-TERT. Telomerase was purified via the O-purification and Strep-Tactin pulldown. Samples were analyzed by immunoblotting at each step of the purification. **(B)** Triplicates of the immunoblotting experiments of the input lysates (input), OE and Strep E from the purification strategy shown in (A). See Fig. 4, E to H for quantification.

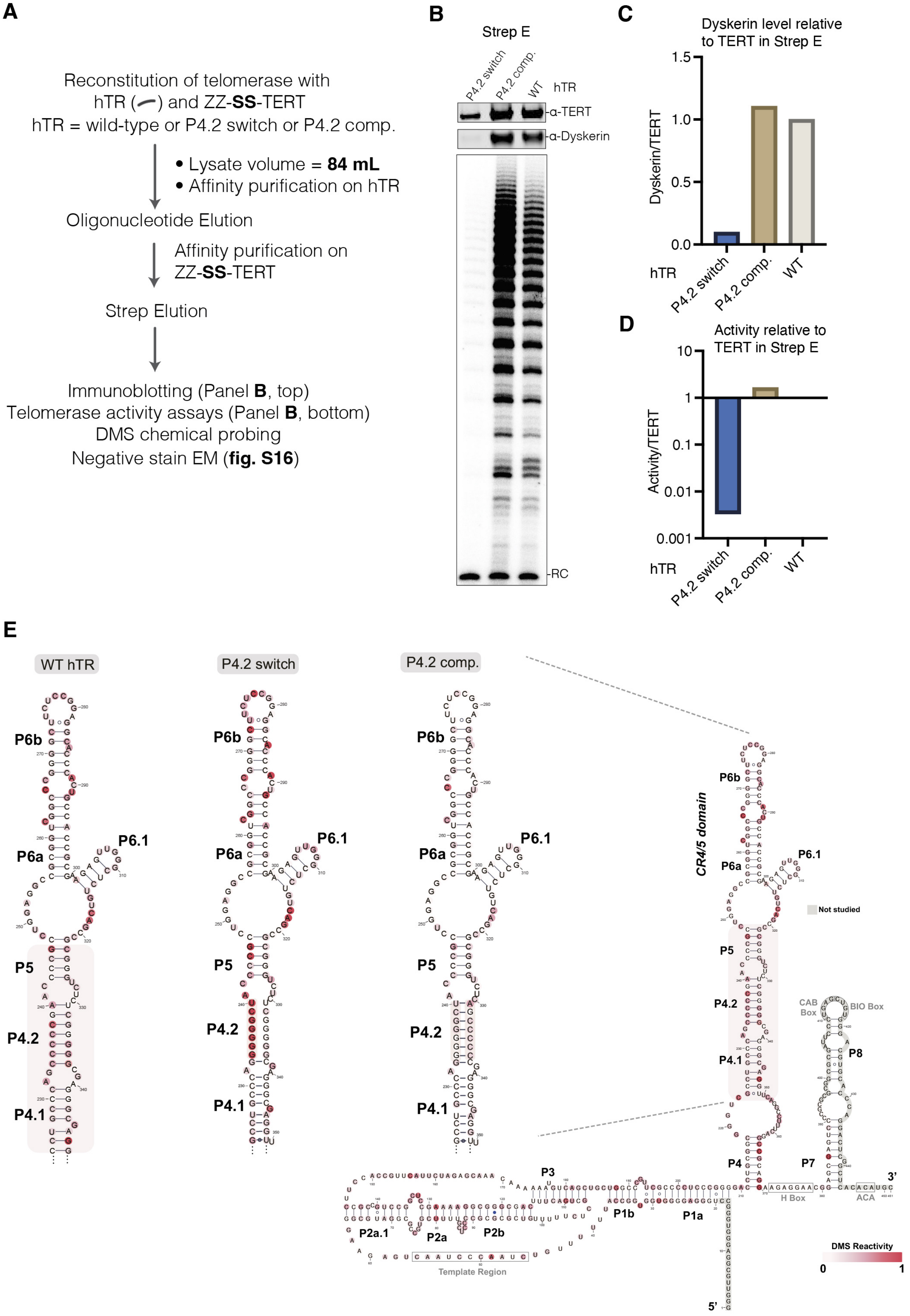
A
Reconstitution of telomerase with
hTR ( ) and ZZ-SS-TERT
hTR = wild-type or P4.2 switch or P4.2 comp.
• Lysate volume = 84 mL
• Affinity purification on hTR
Oligonucleotide Elution
Affinity purification on
ZZ-SS-TERT
Strep Elution
Immunoblotting (Panel B, top)
Telomerase activity assays (Panel B, bottom)
DMS chemical probing
Negative stain EM (fig. S16)
B
Strep E
P4.2 switch
P4.2 comp.
WT
hTR
α-TERT
α-Dyskerin
RC
C
Dyskerin level relative
to TERT in Strep E
Dyskerin/TERT
1.5
1.0
0.5
0.0
hTR
P4.2 switch
P4.2 comp.
WT
D
Activity relative to
TERT in Strep E
Activity/TERT
10
1
0.1
0.01
0.001
hTR
P4.2 switch
P4.2 comp.
WT
E
WT hTR
P4.2 switch
P4.2 comp.
P6b
P6a
P6.1
P5
P4.2
P4.1
CR4/5 domain
Not studied
P4
CAB Box
BIO Box
P8
P7
H Box
ACA
3'
5'
P3
P1b
P1a
P2a.1
P2a
P2b
Template Region
DMS Reactivity
0
1

Fig. S15. Characterization of telomerase reconstituted with P4.2 switch and P4.2 comp. hTR mutants.

(A) Schematic of the strategy to characterize telomerase reconstituted with either WT, P4.2 switch and P4.2 comp. constructs of hTR. WT hTR and the two P4.2 hTR mutants were reconstituted with ZZ-SS-TERT, followed by O-purification and Strep-Tactin pulldown to obtain purified bulk telomerase. The Strep E sample was subjected to immunoblotting, telomerase activity assay, DMS chemical probing and negative staining EM (fig. S16). **(B)** Immunoblotting (top) and telomerase activity assay of the purified telomerase with WT hTR and the P4.2 switch and P4.2 comp. hTR mutants. RC, recovery control. **(C)** Bar graph showing the level of dyskerin relative to TERT in the Strep E fractions of telomerase reconstituted with either WT hTR, the P4.2 switch or P4.2 comp. hTR mutant. Levels were normalized against the telomerase sample with WT hTR. **(D)** Bar graph showing the level of telomerase activity per TERT for the Strep E fractions of telomerase reconstituted with WT hTR, the P4.2 switch or P4.2 comp. hTR mutant. Levels were normalized against the telomerase sample with WT hTR. The Y-axis is shown in $\log_{10}$-scale due to the low level of telomerase activity in the P4.2 switch mutant. **(E)** DMS chemical probing of human telomerase RNA in the purified telomerase complexes reconstituted with the WT, the P4.2 switch and the P4.2 comp. mutant constructs of hTR. Samples were purified as shown in (A). The overall RNA secondary structure of the WT hTR in the purified complex with DMS reactivities is shown. The RNA secondary structures across the P4.1 and P4.2 stems and the CR4/5 region with DMS reactivities in the WT hTR and the P4.2 switch and P4.2 comp. mutants are shown in the close-up views. RNA regions that are used for the two primer-binding sites and that are close to the 5’ and 3’ ends cannot be assessed; these regions are shaded with a light grey box.

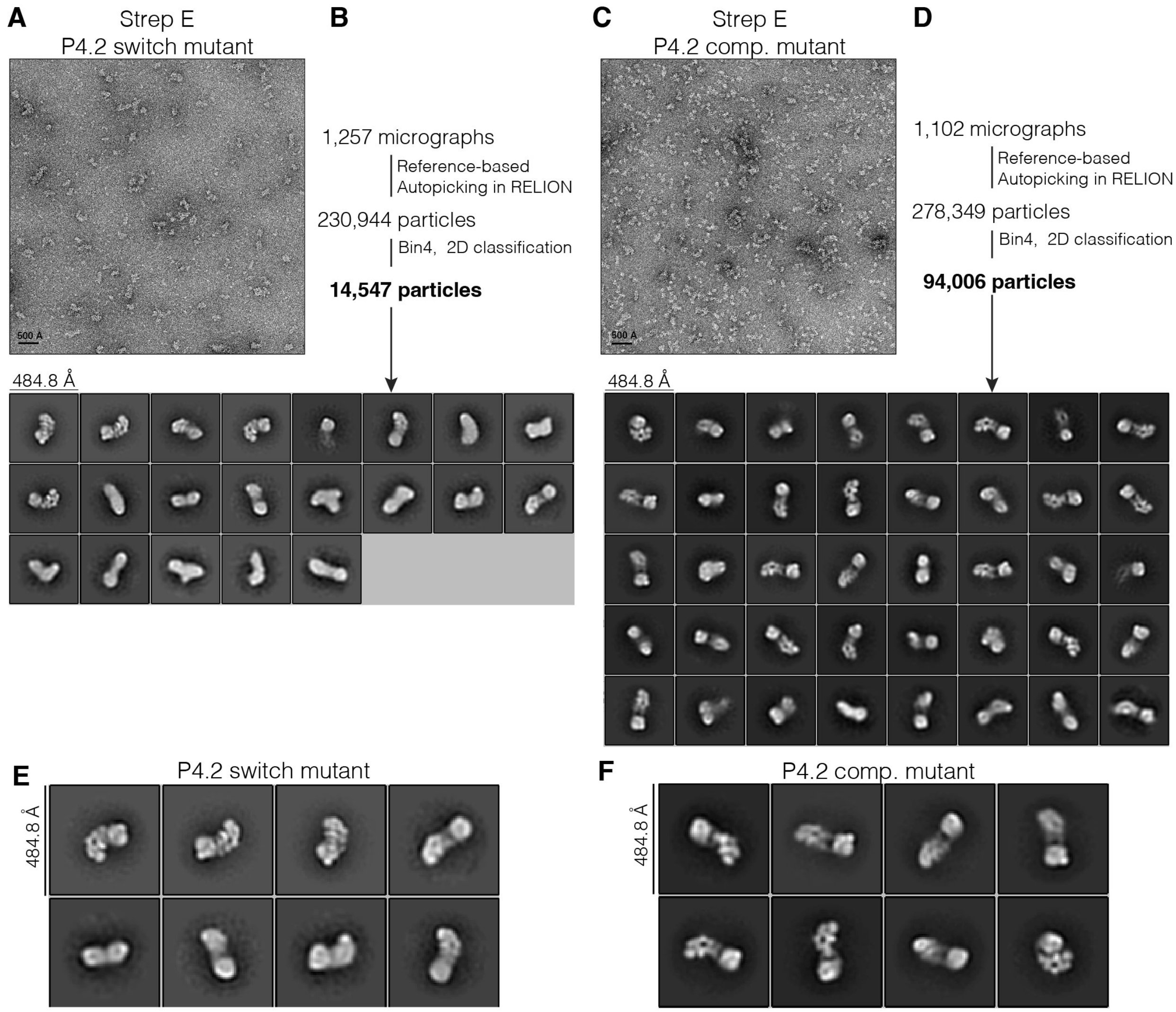

Fig. S16. Visualization of the purified hTR P4.2 switch and P4.2 comp. telomerase mutants by negative stain EM.
(A) Representative negative stain EM image of the telomerase P4.2 switch hTR mutant purified using the schematic shown in fig. S15A. **(B)** Data processing strategy to obtain the 2D class averages for the telomerase P4.2 switch hTR mutant. **(C)** Representative negative stain EM image of the telomerase P4.2 comp. hTR mutant purified using the schematic shown in fig. S15A. **(D)** Data processing strategy to obtain the 2D class averages for the telomerase P4.2 comp. hTR mutant. **(E and F)** Representative negative stain EM 2D class averages for the P4.2 switch and P4.2 comp. hTR mutants, respectively, for comparison.

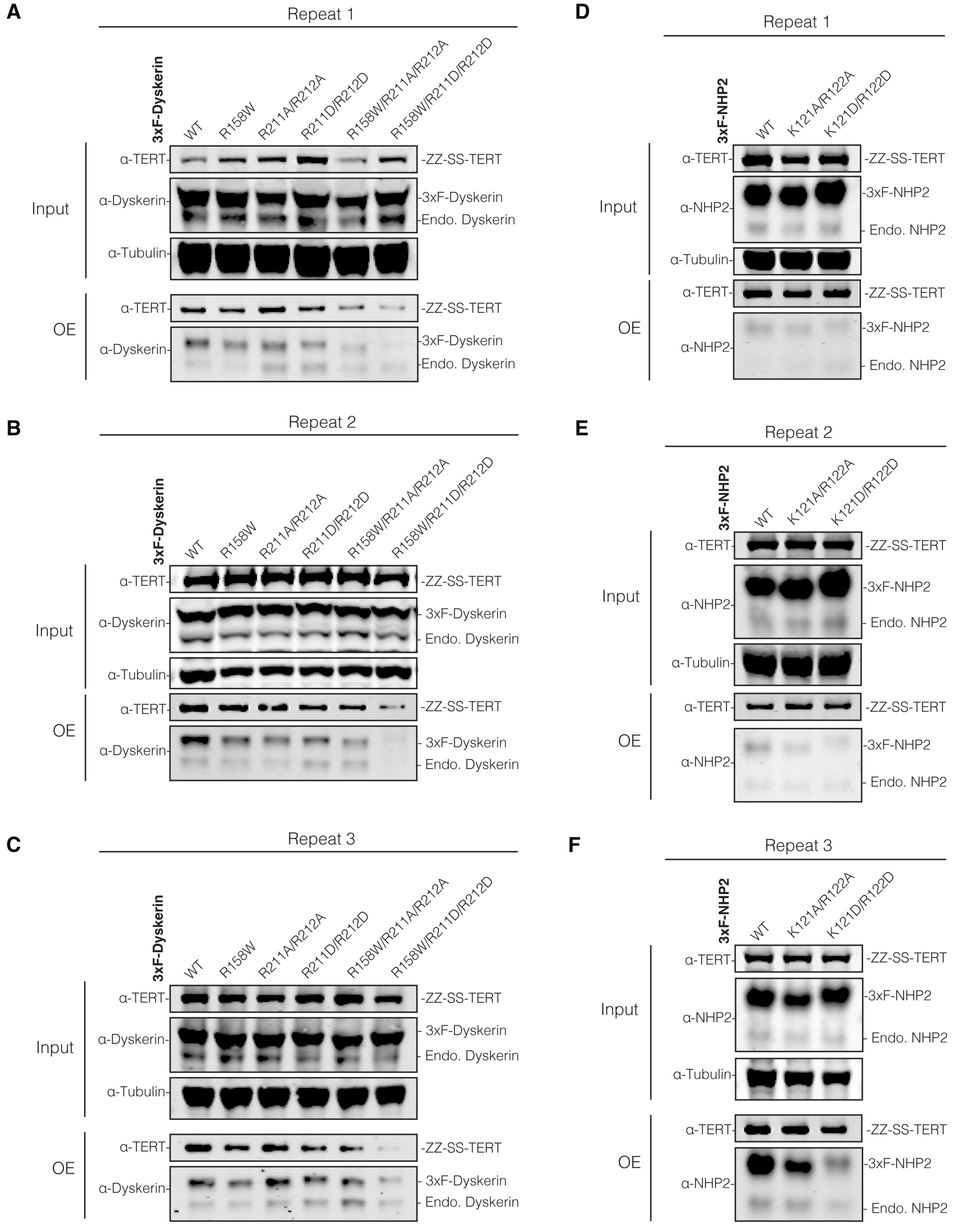

Fig. S17. Mutations of dyskerin and NHP2 that disrupt dimerization result in defects in telomerase incorporation.

(A to C) Triplicates of experiments in which residues crucial for dimerization in dyskerin were mutated and reconstituted with TERT and hTR. **(D to F)** Triplicates of experiments in which residues crucial for dimerization in NHP2 were mutated and reconstituted with TERT and hTR. In

all experiments, telomerase was purified via hTR using O-purification. The amounts of the overexpressed WT and mutant dyskerin or NHP2 relative to the corresponding endogenous protein were compared to test for incorporation defects. Also see Fig. 5, E and F for quantification.

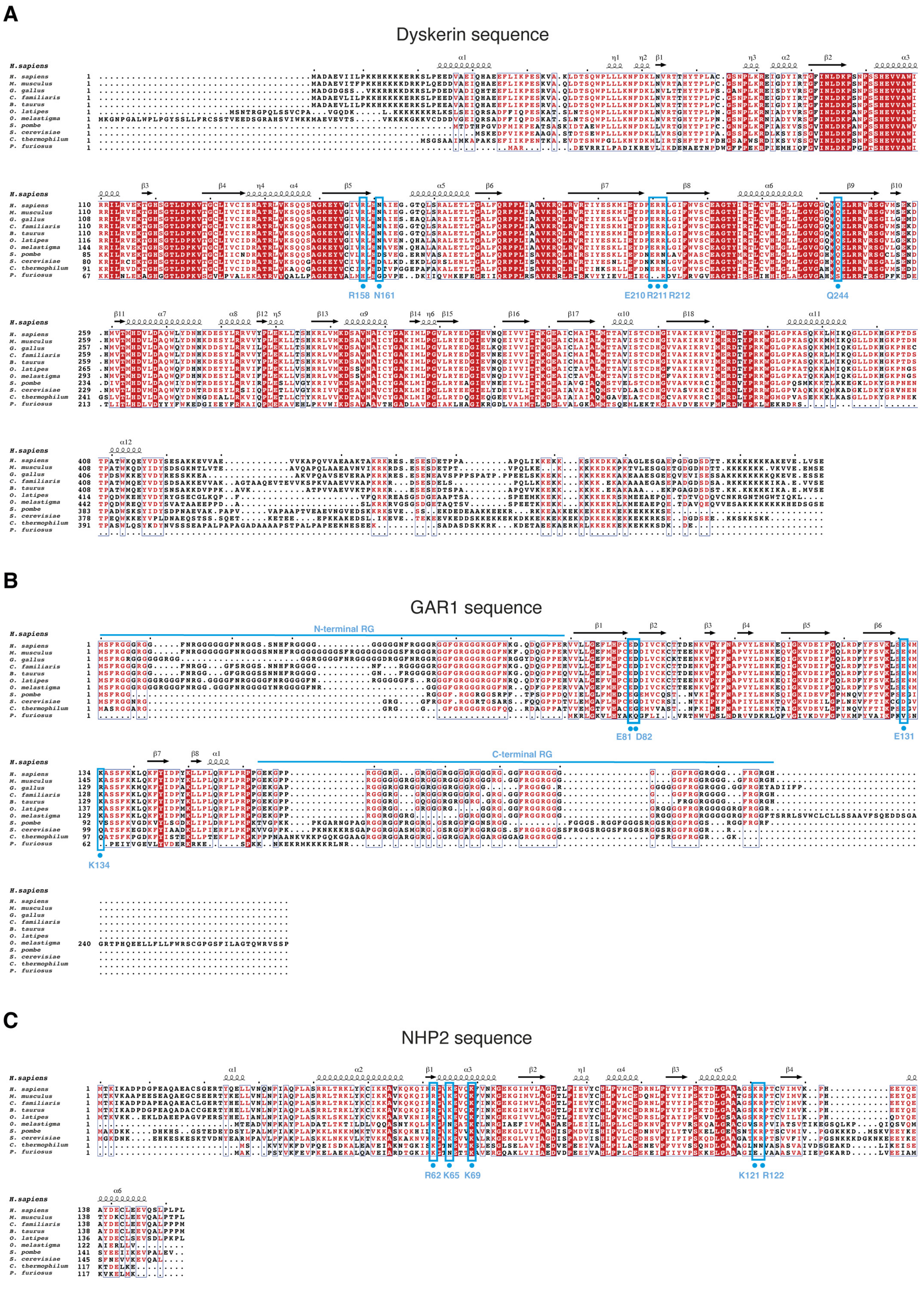
A
Dyskerin sequence
R158 N161
E210 R211 R212
Q244
B
GAR1 sequence
N-terminal RG
E81 D82
E131
C-terminal RG
K134
C
NHP2 sequence
R62 K65 K69
K121 R122

Fig. S18. Sequence alignments of dyskerin, GAR1 and NHP2.

(A) to **(C)** Sequence alignments of dyskerin (A), GAR1 (B), and NHP2 (C). Sequences of dyskerin and GAR1 from 11 species, and NHP2 from 10 species, were obtained from UniProt and aligned using Clustal Omega (*82*). ESPript 3.0 was utilized for the visualization of aligned sequences (*83*). Residues with 70% sequence conservation are colored, with red boxes indicating absolute conservation. Residues highlighted in the text and figures are labelled with a blue box and a blue dot.

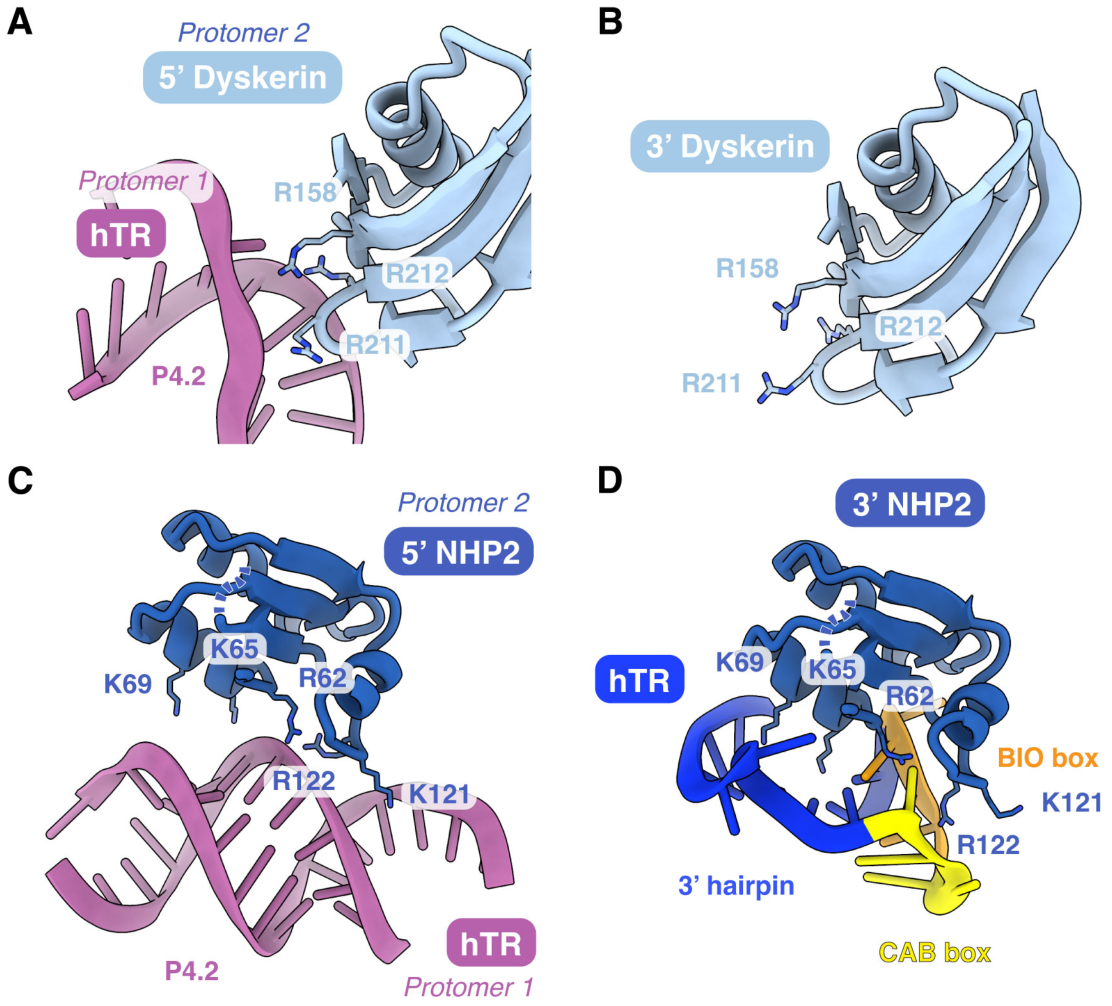

Fig. S19. hTR interaction with NHP2 and dyskerin of the 5' and 3' H/ACA heterotetramers. **(A)** Interaction between hTR of H/ACA RNP protomer 1 and the 5' dyskerin of H/ACA RNP protomer 2. **(B)** The 3' dyskerin surface equivalent to the one shown in (A). This region does not form interactions with the 3' H/ACA hairpin of hTR. **(C)** Interaction between hTR of H/ACA RNP protomer 1 and the 5' NHP2 of H/ACA RNP protomer 2. **(D)** Interaction between the 3' hairpin of hTR and the 3' NHP2, showing the same region of NHP2 as shown in (C).

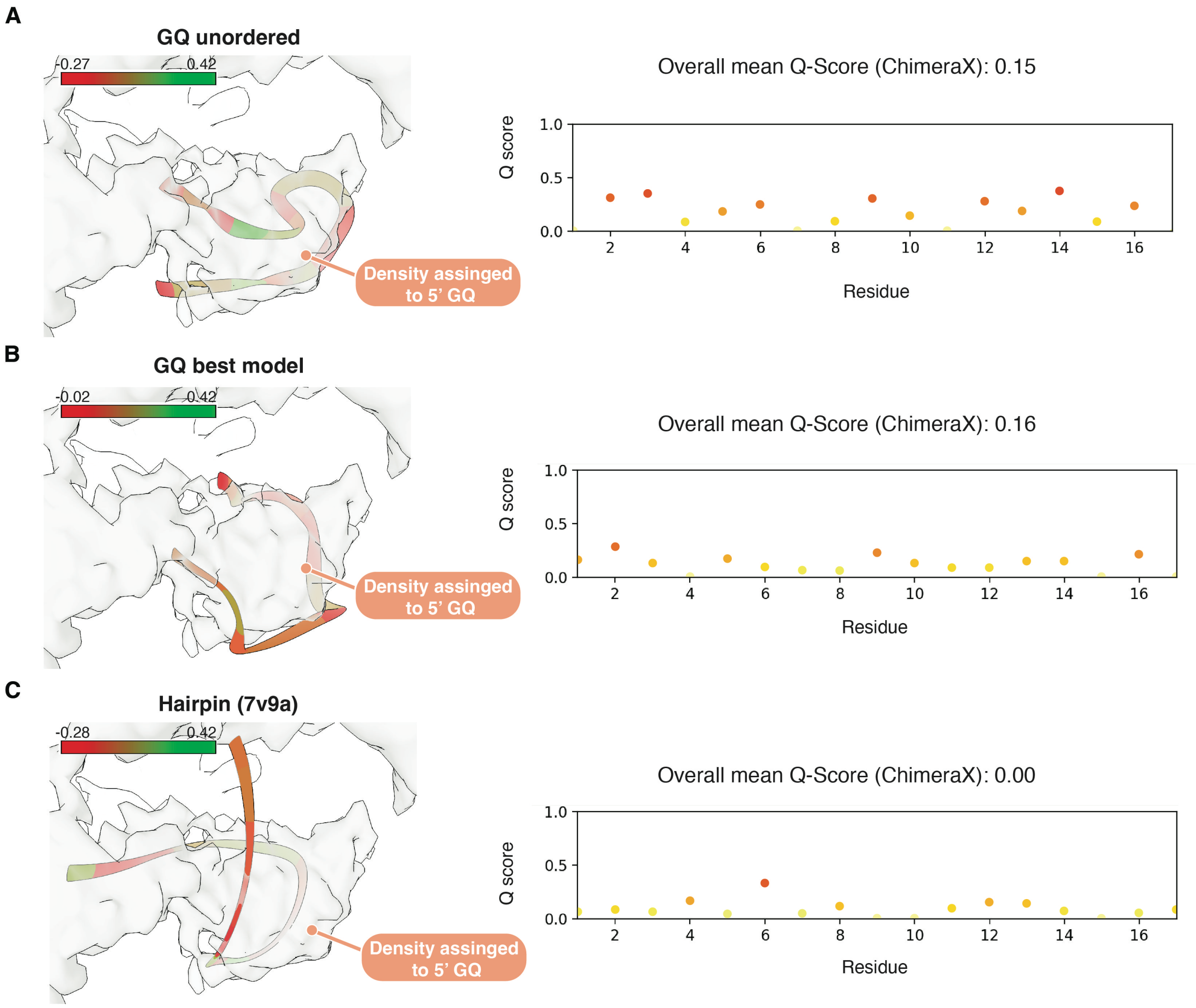

Fig. S20. Q-scores for the 5' leader sequence modelled as unordered 5' GQ, ordered 5' GQ, and 5' hairpin.

(A to C) Unordered model, ordered 5' GQ (best fit), and 5' hairpin (PDB 7V9A), respectively, fitted within the 3.9 Å H/ACA RNP dimer map and colored by the backbone Q-scores generated in UCSF Chimera (*10, 79, 80*). The right panels show the backbone Q-scores for individual residues (nucleotides 1–17 of hTR), generated and visualized in UCSF ChimeraX (*77, 79*).

Table S1. Cryo-EM data collection, refinement and statistics for the consensus map and the H/ACA RNP.

	Consensus dimer	H/ACA RNP protomer	H/ACA RNP dimer
		PDB 9QB2	PDB 9QB3
	EMD-52976	EMD-52983	EMD-52984
**Data collection and Processing**			
Microscope		Titan Krios G3i	
Voltage (keV)		300	
Camera		Gatan K3	
Magnification		81,000; 45,782	
Pixel size at detector (Å/pixel)		1.059	
Total electron exposure (e^{-}/Å^2)		47–50	
Exposure rate (e-/ Å^2/sec)		15.66	
Number of frames		48	
Defocus range (μm)		0.8–2.4	
Automation software		EPU	
Energy filter slit width		20 eV	
Micrographs collected (no.)		66,992	
Total extracted particles (no.)		14,236,535	
**For each reconstruction:**			
Final particles (no.)	505,039	260,466	88,419
Point-group	C1	C1	C2
Estimated error (translations/rotations)		0.554/1.116	0.822/1.233
Resolution (global, Å)	6.2	3.0	3.9
FSC 0.5 (unmasked/masked)	9.3/7.8	4.1/3.3	6.7/4.3
FSC 0.143 (unmasked/masked)	7.6/6.2	3.4/3.0	4.3/3.9
Resolution range (local, Å)	5.6–24.0	2.9–8.2	3.7–10.8
3DFSC Sphericity	0.758	0.954	0.838
Map sharpening *B* factor (Å^2)	NA	-100	-50
Map sharpening methods	RELION5.0	RELION5.0	RELION5.0
**Model composition**			
Protein (residues)	NA	1674	3348
RNA/DNA (nucleotides)	NA	297	372
**Model Refinement**			
Refinement package	NA	Servalcat/REFMAC5.8	Servalcat/REFMAC5.8
- real or reciprocal space	NA	Reciprocal Space	Reciprocal Space
- resolution cutoff	NA	0.5	0.5
Model-Map scores	NA		
- CCvolume/mask	NA	0.75/0.75	0.76/0.78
B factors (Å^2)			
Protein residues (min/max/mean)	NA	34.97/444.10/123.31	39.53/721.76/269.62
RNA/DNA (min/max/mean)	NA	33.03/580.27/303.75	5349.50/636.00/373.95
R.m.s. deviations from ideal values			
Bond lengths (Å) (#>4σ)	NA	0.009 (1)	0.009 (1)
Bond angles (°) (#>4σ)	NA	1.287 (4)	1.297 (17)
**Validation**			
MolProbity score	NA	0.82	0.93
CaBLAM outliers (%)	NA	1.17%	2.6%
Clashscore	NA	1.13	1.73
Poor rotamers (%)	NA	0.27	0.27
C-beta deviations (%)	NA	0	0
EMRinger score	NA	3.79	1.01
Ramachandran plot	NA		
Favored (%)	NA	98.18	98.06
Outliers (%)	NA	0	0

Table S2. Cryo-EM map and model refinement, and validation statistics for the catalytic core

	**Catalytic Core protomer with symmetry expansion** PDB 9QAX EMD-52978	**Catalytic Core Protomer 1** PDB 9QAY EMD-52979	**Catalytic Core Protomer 2** PDB 9QAZ EMD-52980	**Catalytic Core Protomer 1 (shared subset)** EMD-52981	**Catalytic Core Protomer 2 (shared subset)** EMD-52982
**<u>For each reconstruction:</u>**					
Final particles (no.)	272,757	138,966	133,259	41,195	41,195
Point-group	C1	C1	C1	C1	C1
Estimated error (translations/rotations)	0.756/1.502	0.831/1.807	0.772/1.591	0.892/1.95	0.821/1.74
Resolution (global, Å)	3.3	3.8	3.6	4.1	3.9
FSC 0.5 (unmasked/masked)	4.2/3.7	6.8/4.3	6.9/4.0	8.5/6.1	8.6/6.1
FSC 0.143 (unmasked/masked)	3.7/3.3	4.1/3.8	4.0/3.6	4.6/4.1	4.3/3.9
Resolution range (local, Å)	3.1–6.4	3.7–8.0	3.3–8.0	3.9–9.8	3.6–11.9
3DFSC Sphericity	0.871	0.822	0.822	0.760	0.786
Map sharpening *B* factor (Å^2)	-40	-50	-50	-70	-70
Map sharpening methods	RELION5.0	RELION5.0	RELION5.0		
**Model composition**					
Protein (residues)	1247	1247	1247		
RNA/DNA (nucleotides)	260	260	260		
**Model Refinement**					
Refinement package	Servalcat/REFMAC5.8	Servalcat/REFMAC5.8	Servalcat/REFMAC5.8		
- real or reciprocal space	Reciprocal Space	Reciprocal Space	Reciprocal Space		
- resolution cutoff	0.5	0.5	0.5		
Model-Map scores	3.5	4.0	3.7		
- CCvolume/mask	0.76/0.77	0.75/0.76	0.73/0.74		
B factors (Å^2)					
Protein residues (min/max/mean)	37.23/964.00/278.33	39.80/999.00/314.56	38.08/999.00/281.70		
RNA/DNA (min/max/mean)	41.48/999.00/324.40	41.08/999.00/351.00	46.37/999.00/358.74		
R.m.s. deviations from ideal values					
Bond lengths (Å) (#>4σ)	0.009 (0)	0.009 (0)	0.009 (0)		
Bond angles (°) (#>4σ)	1.203 (5)	1.228 (8)	1.232 (6)		
**Validation**					
MolProbity score	1.81	1.81	1.82		
CaBLAM outliers (%)	2.9%	2.73%	2.9%		
Clashscore	6.76	6.94	6.97		
Poor rotamers (%)	0.09	0.09	0.09		
C-beta deviations (%)	0.09	0.17	0.09		
EMRinger score	0.94	0.39	1.03		

Ramachandran plot			
Favored (%)	93.32	93.40	93.32
Outliers (%)	0.41	0.41	0.41

Table S3.

Sequences of DNA primers used for mutagenesis of hTR, TERT, dyskerin and NHP2.

Construct	Mutation	Primers
pcDNA3.1-U3-hTR-HDV	ΔU329	5’CTCGGGGGCGAGGGC3’
		5’GACCCGCGGCTGACAG3’
pcDNA3.1-U3-hTR-HDV	ΔUC329–330	5’TCGGGGGCGAGGGCG3’
		5’GACCCGCGGCTGACAGAG3’
pcDNA3.1-U3-hTR-HDV	UC329–330GG	5’GCCGCGGGTCGGTCGGGGGCGA3’
		5’TGACAGAGCCCAACTCTTCG3’
pcDNA3.1-U3-hTR-HDV	P4.2 switch	5’GGCTACCCCGCCTGGAGGC3’
		5’CCCCTGGGCAGGCGACCC3’
pcDNA3.1-U3-hTR-HDV	P4.2 comp.	5’CCCCCGAGGGCGAGGTTCAG3’
		5’GCTGAGACCCGCGGCTGAC3’
pcDNA3.1-3xFlag-dyskerin	R158W	5’GGGGATTGTCTGGCTGCACAA3’
		5’ACATACTCTTTGCCTGCACTC3’
pcDNA3.1-3xFlag-dyskerin	R211A & R212A	5’CGATCCTGAAGCGGCTTTAGGAATCTTTTGGG3’
		5’TATTCAATCATTTTGCTCTCG3’
pcDNA3.1-3xFlag-dyskerin	R211D & R212D	5’CGATCCTGAAGATGACTTAGGAATCTTTTGGGTG3’
		5’TATTCAATCATTTTGCTCTCG3’
pcDNA3.1-3xFlag-NHP2	K121A & R122A	5’CGCAGGCTCCGCGGCTCCCACCTGTGTGATAATG3’
		5’GCTGCACCCAGGTCCG3’
pcDNA3.1-3xFlag-NHP2	K121D & R122D	5’CGCAGGCTCCGACGATCCCACCTGTGTGATAATGG TCAAG3’
		5’GCTGCACCCAGGTCCG3’
pcDNA3.1-SmBiT-ZZ-TEV-twin-Strep-SUMO*-TERT	WT	5’GCTGGCTAGCGCTGCCACCATGGTCACCGGCTACC GG3’
		5’CGCGTTTTGTTGTTCTTTGTTGAATTTGTTGTCCAC TCCAGAACTTCCACCTCC3’
pcDNA3.1-LgBiT-ZZ-TEV-twin-Strep-SUMO*-TERT	WT	5’GCTGGCTAGCGCTGCCACCATGGTGTTCACCCTGG AAGATTTC3’
		5’CGCGTTTTGTTGTTCTTTGTTGAATTTGTTGTCCAC GGATCCACTTCCACCTCC3’
pcDNA3.1-SmBiT-ZZ-TEV-3xFlag-TERT	WT	5’GCTGGCTAGCGCTGCCACCATGGTCACCGGCTACC GG3’
		5’CGCGTTTTGTTGTTCTTTGTTGAATTTGTTGTCCAC TCCAGAACTTCCACCTCC3’
pcDNA3.1-LgBiT-ZZ-TEV-3xFlag-TERT	WT	5’GCTGGCTAGCGCTGCCACCATGGTGTTCACCCTGG AAGATTTC3’
		5’CGCGTTTTGTTGTTCTTTGTTGAATTTGTTGTCCAC GGATCCACTTCCACCTCC3’
pcDNA3.1-ZZ-TEV-twin-Strep-SUMO*-TERT	D868A & D869A	5’CTCCTGCGTTTGGTGGCGGCTTTCTTGTTGGTGAC ACC3’
		5’GGTGTCACCAACAAGAAAGCCGCCACCAAACGCA GGAG3’
pcDNA3.1-ZZ-TEV-3xFlag-TERT	D868A & D869A	5’CTCCTGCGTTTGGTGGCGGCTTTCTTGTTGGTGAC ACC3’
		5’GGTGTCACCAACAAGAAAGCCGCCACCAAACGCA GGAG3’
pcDNA3.1-ZZ-TEV-twin-Strep-SUMO*-TERT	D712A	5’CTTTGTCAAGGTGGCGGTGACGGGCGCG3’
		5’CGCGCCCGTCACCGCCACCTTGACAAAG3’
pcDNA3.1-ZZ-TEV-3xFlag-TERT	D712A	5’CTTTGTCAAGGTGGCGGTGACGGGCGCG3’
		5’CGCGCCCGTCACCGCCACCTTGACAAAG3’
pcDNA3.1-ZZ-TEV-twin-Strep-SUMO*-TERT	Δ200–325 (PAL)	5’GCCGAGACCAAGCAC3’
		5’GCAGACCCTACGCTT3’
pcDNA3.1-ZZ-TEV-3xFlag-TERT	Δ200–325 (PAL)	5’GCCGAGACCAAGCAC3’
		5’GCAGACCCTACGCTT3’

Movie S1.

3D variability analysis of the full telomerase dimer performed in CryoSPARC.

Data S1. Pymol session containing the DRRAFTER models of hTR.

Fitted models of the catalytic cores and the H/ACA RNPs in the consensus dimer map are included in the session.

Data S2. Q-scores for different models of the 5' leader sequence of hTR.

Calculated Q-scores for different models of the 5' leader sequence of hTR (nucleotides 1–17). See also fig. S20.

Data S3. Pymol session containing the DRRAFTER models of the 5' G-quadruplex of hTR.

These models were performed using the 3.9 Å reconstruction of the H/ACA RNP dimer. The refined model of the H/ACA RNP dimer is also included in the session as a reference.

References

81. S. Aiyer, C. Zhang, P. R. Baldwin, D. Lyumkis, Evaluating Local and Directional Resolution of Cryo-EM Density Maps. *Methods Mol Biol* **2215**, 161-187 (2021).
82. F. Sievers *et al.*, Fast, scalable generation of high-quality protein multiple sequence alignments using Clustal Omega. *Mol. Syst. Biol.* **7**, 539 (2011).
83. X. Robert, P. Gouet, Deciphering key features in protein structures with the new ENDscript server. *Nucleic Acids Res.* **42**, W320-W324 (2014).
